# Use and Safety of Immunotherapeutic Management of *N*-Methyl-d-Aspartate Receptor Antibody Encephalitis: A Meta-analysis

**DOI:** 10.1001/jamaneurol.2021.3188

**Published:** 2021-09-20

**Authors:** Margherita Nosadini, Michael Eyre, Erika Molteni, Terrence Thomas, Sarosh R. Irani, Josep Dalmau, Russell C. Dale, Ming Lim, and the International NMDAR Antibody Encephalitis Consensus Group, Banu Anlar, Thaís Armangue, Susanne Benseler, Tania Cellucci, Kumaran Deiva, William Gallentine, Grace Gombolay, Mark P. Gorman, Yael Hacohen, Yuwu Jiang, Byung Chan Lim, Eyal Muscal, Alvin Ndondo, Rinze Neuteboom, Kevin Rostásy, Hiroshi Sakuma, Stefano Sartori, Suvasini Sharma, Silvia Noemi Tenembaum, Heather Ann Van Mater, Elizabeth Wells, Ronny Wickstrom, Anusha K. Yeshokumar

**Affiliations:** 1 Paediatric Neurology and Neurophysiology Unit, Department of Women’s and Children’s Health, University Hospital of Padova, Padova, Italy; 2 Neuroimmunology Group, Paediatric Research Institute “Città della Speranza,” Padova, Italy; 3 School of Biomedical Engineering and Imaging Sciences, King’s College London, London, United Kingdom; 4 Children’s Neurosciences, Evelina London Children’s Hospital at Guy’s and St Thomas’ NHS Foundation Trust, London, United Kingdom; 5 Centre for Medical Engineering, King’s College London, London, United Kingdom; 6 Department of Paediatrics, Neurology Service, KK Women’s and Children’s Hospital, Singapore; 7 Oxford Autoimmune Neurology Group, Nuffield Department of Clinical Neurosciences, University of Oxford, Oxford, United Kingdom; 8 Department of Neurology, Oxford University Hospitals NHS Foundation Trust, Oxford, United Kingdom; 9 Neuroimmunology Program, Institut d’Investigacions Biomèdiques August Pi i Sunyer (IDIBAPS), Hospital Clínic, University of Barcelona, Barcelona, Spain; 10 Department of Neurology, University of Pennsylvania, Philadelphia; 11 Institució Catalana de Recerca i Estudis Avançats (ICREA), Barcelona, Spain; 12 Kids Neuroscience Centre, The Children’s Hospital at Westmead, Faculty of Medicine and Health, University of Sydney, Westmead, Australia; 13 Department of Women and Children’s Health, School of Life Course Sciences (SoLCS), King’s College London, London, United Kingdom; 14 Hacettepe University, Ankara, Turkey; 15 Sant Joan de Déu (SJD) Children’s Hospital, University of Barcelona, Barcelona, Spain; 16 Alberta Children’s Hospital Research Institute, Cumming School of Medicine, University of Calgary, Calgary, Alberta, Canada; 17 McMaster University, Hamilton, Ontario, Canada; 18 Assistance Publique-Hôpitaux de Paris, University Hospitals Paris Saclay, Bicêtre Hospital, Paris, France; 19 French Reference Network of Rare Inflammatory Brain and Spinal Diseases, Paris, France; 20 European Reference Network-RITA, Paris, France; 21 Stanford University and Lucile Packard Children’s Hospital, Palo Alto, California; 22 Emory University School of Medicine and Children’s Healthcare of Atlanta, Atlanta, Georgia; 23 Boston Children’s Hospital, Harvard Medical School, Boston, Massachusetts; 24 Queen Square MS Centre, UCL Institute of Neurology, University College London, London, United Kingdom; 25 Department of Paediatric Neurology, Great Ormond Street Hospital for Children, London, United Kingdom; 26 Peking University First Hospital, Beijing, China; 27 Pediatric Clinical Neuroscience Center, Seoul National University Children’s Hospital, Seoul National University College of Medicine, Seoul, Republic of Korea; 28 Section Rheumatology, Texas Children’s Hospital, Baylor College of Medicine, Houston; 29 Child Health, Red Cross War Memorial Children’s Hospital, University of Cape Town, Cape Town, South Africa; 30 Faculty of Health Sciences, University of Cape Town Neuroscience Institute, Cape Town, South Africa; 31 Erasmus Medical Center, Rotterdam, the Netherlands; 32 Children’s Hospital Datteln, University Witten/Herdecke, Witten, Germany; 33 Tokyo Metropolitan Institute of Medical Science, Tokyo, Japan; 34 University Hospital of Padova, Padova, Italy; 35 Lady Hardinge Medical College and Associated Kalawati Saran Children’s Hospital, New Delhi, India; 36 National Pediatric Hospital Dr J. Garrahan, Buenos Aires, Argentina; 37 Duke University, Durham, North Carolina; 38 Children’s National Medical Center, Washington, DC; 39 Karolinska University Hospital, Stockholm, Sweden; 40 Icahn School of Medicine at Mount Sinai, New York, New York

## Abstract

**Importance:**

Overall, immunotherapy has been shown to improve outcomes and reduce relapses in individuals with *N*-methyl-d-aspartate receptor (NMDAR) antibody encephalitis (NMDARE); however, the superiority of specific treatments and combinations remains unclear.

**Objective:**

To map the use and safety of immunotherapies in individuals with NMDARE, identify early predictors of poor functional outcome and relapse, evaluate changes in immunotherapy use and disease outcome over the 14 years since first reports of NMDARE, and assess the Anti-NMDAR Encephalitis One-Year Functional Status (NEOS) score.

**Data Sources:**

Systematic search in PubMed from inception to January 1, 2019.

**Study Selection:**

Published articles including patients with NMDARE with positive NMDAR antibodies and available individual immunotherapy data.

**Data Extraction and Synthesis:**

Individual patient data on immunotherapies, clinical characteristics at presentation, disease course, and final functional outcome (modified Rankin Scale [mRS] score) were entered into multivariable logistic regression models.

**Main Outcomes and Measures:**

The planned study outcomes were functional outcome at 12 months from disease onset (good, mRS score of 0 to 2; poor, mRS score greater than 2) and monophasic course (absence of relapse at 24 months or later from onset).

**Results:**

Data from 1550 patients from 652 articles were evaluated. Of these, 1105 of 1508 (73.3%) were female and 707 of 1526 (46.3%) were 18 years or younger at disease onset. Factors at first event that were significantly associated with good functional outcome included adolescent age and first-line treatment with therapeutic apheresis, corticosteroids plus intravenous immunoglobulin (IVIG), or corticosteroids plus IVIG plus therapeutic apheresis. Factors significantly associated with poor functional outcome were age younger than 2 years or age of 65 years or older at onset, intensive care unit admission, extreme delta brush pattern on electroencephalography, lack of immunotherapy within the first 30 days of onset, and maintenance IVIG use for 6 months or more. Factors significantly associated with nonrelapsing disease were rituximab use or maintenance IVIG use for 6 months or more. Adolescent age at onset was significantly associated with relapsing disease. Rituximab use increased from 13.5% (52 of 384; 2007 to 2013) to 28.3% (311 of 1100; 2013 to 2019) (*P* < .001), concurrent with a falling relapse rate over the same period (22% [12 of 55] in 2008 and earlier; 10.9% [35 of 322] in 2017 and later; *P* = .006). Modified NEOS score (including 4 of 5 original NEOS items) was associated with probability of poor functional status at 1 year (20.1% [40 of 199] for a score of 0 to 1 points; 43.8% [77 of 176] for a score of 3 to 4 points; *P* = .05).

**Conclusions and Relevance:**

Factors influencing functional outcomes and relapse are different and need to be considered independently in development of evidence-based optimal management guidelines of patients with NMDARE.

## Key Points

**Question:**

What are the most effective treatments for *N*-methyl-d-aspartate receptor (NMDAR) antibody encephalitis?

**Findings:**

In this meta-analysis of individual patient data including 1550 cases, treatment factors at first event that were significantly associated with good functional outcome 12 months from disease onset included first-line treatment with therapeutic apheresis alone, corticosteroids in combination with intravenous immunoglobulin (IVIG), or corticosteroids in combination with IVIG and therapeutic apheresis, while lack of immunotherapy within 30 days of disease onset was significantly associated with poor outcome. Rituximab and long-term IVIG use were significantly associated with nonrelapsing disease course.

**Meaning:**

Separate treatment factors are associated with functional outcomes and relapsing disease biology in those with NMDAR antibody encephalitis.

## Teaser

This meta-analysis evaluates the use and safety of immunotherapies among patients with *N*-methyl-d-aspartate receptor antibody encephalitis.

## Introduction

*N*-methyl-d-aspartate receptor (NMDAR) antibody encephalitis (NMDARE) is the most common autoimmune encephalitis, predominantly affecting children and young adults. Management is challenging owing to the frequently severe disease in the acute phase, often requiring prolonged hospitalization, the variable and unpredictable outcomes, and the possibility of relapses.^1^ Some patients recover fully, but many experience long-term cognitive and psychiatric problems, with significant effects on education, employment, and quality of life.^2^ Overall, immunotherapy improves functional outcome^1,3,4,5^ and reduces relapses,^1,6,7^ but many therapeutic questions remain incompletely answered, including the effects of specific immunotherapies on outcome and risk of relapses and the role of second-line and maintenance long-term immunotherapies.

While a number of reviews have been published,^8,9,10,11,12,13^ to our knowledge, no definite guiding data or treatment guidelines are available. Treatment strategies are heterogeneous, especially regarding second-line and maintenance immunotherapy.^14,15^ Here, we have performed a systematic literature review and evidence synthesis of all published patients with NMDARE with available individualized immunotherapy data, toward 4 main aims: (1) mapping the use and safety of immunotherapies; (2) identifying early predictors of poor outcome and risk of relapse; (3) evaluating changes in immunotherapy use and disease outcome over the 14 years since first reports of NMDARE; and (4) providing an assessment of the Anti-NMDAR Encephalitis One-Year Functional Status (NEOS) score.^16^

## Methods

A systematic search in PubMed was conducted from inception to January 1, 2019 (M.N.), with the following search keys: (*anti–N-methyl-d-aspartate receptor encephalitis*) OR (*N-methyl-d-aspartate antibody encephalitis*) OR (*anti-NMDAR encephalitis*) OR (*anti-NMDA receptor encephalitis*) OR (*NMDA receptor encephalitis*) OR (*anti-N-methyl-d-aspartate receptor antibody encephalitis*). Publications were eligible if they (1) included patients with NMDARE with positive NMDAR antibodies in serum and/or cerebrospinal fluid (CSF) and (2) provided individual patient data on immunotherapy. Patients with NMDARE preceded by central nervous system infection (ie, herpes simplex virus encephalitis) and large cohorts where individual patient details were not available^1^ were omitted. This study followed the Preferred Reporting Items for Systematic Reviews and Meta-analyses (PRISMA) reporting guideline.

### Study Definitions

Abnormal investigation findings were defined as presence of diffuse slow, disorganized, or epileptic activity or extreme delta brush on electroencephalography (EEG)^17^; presence of CSF pleocytosis of 5 cells/uL or more or intrathecal oligoclonal bands^16,17^; or presence of increased T2 or fluid-attenuated inversion recovery parenchymal signal intensity or contrast enhancement on brain magnetic resonance imaging (MRI). Neurological severity in the acute phase and outcome at last follow-up were assessed via the modified Rankin Scale (mRS) score.^18^ When not reported in the original article, mRS score was retrospectively assigned following review of adequate clinical data provided. Good outcome was defined as a final mRS score of 0 to 2 assigned within 12 months of disease onset (inferring mRS score of 0 to 2 at 12 months) and poor outcome as a final mRS score of 3 to 5 assigned after 12 months from disease onset (inferring mRS score of 3 to 5 at 12 months) or mRS score of 6 (death from NMDARE) at any time. Patients with a final mRS score of 0 to 2 assigned after 12 months or a final mRS score of 3 to 5 assigned before 12 months were excluded, as mRS score at 12 months could not be inferred. Monophasic course was the absence of relapse 24 months or more from disease onset.

First-line immunotherapy included corticosteroids, intravenous immunoglobulin (IVIG), and/or therapeutic apheresis. Second-line immunotherapy included rituximab and/or cyclophosphamide. Long-term (6 months or more) maintenance immunotherapy included monthly pulses or daily oral corticosteroids, monthly IVIG, rituximab redosing, and/or steroid-sparing agents (oral mycophenolate mofetil, azathioprine, or methotrexate). Early treatment was defined as initiation of immunotherapy within 30 days from first recognizable disease feature.^16,19,20^ Adverse events to immunotherapy were categorized according to the National Institutes of Health Common Terminology Criteria for Adverse Events (CTCAE) version 5.0, with a focus on severe events (grade 3 to 5). Changes in immunotherapy use and disease outcome with time were primarily analyzed over 2 epochs: before and after 2013 (Titulaer et al^1^; eMethods 1 in the Supplement). Additional post hoc analyses over 6 epochs (with cutoffs at 2-year intervals) were performed for rituximab use, relapse rate, and functional outcome only.

### NEOS Score Validation

The NEOS score is a tool for prediction of 1-year functional status in patients with NMDARE determined from 5 patient characteristics, each scoring 1 point.^16^ One of the 5 (no clinical improvement after 4 weeks of treatment) was not usually specified in published reports, so we instead applied a modified NEOS (mNEOS) score including 4 items, scoring 1 point each: intensive care unit (ICU) admission, no treatment within 30 days of symptom onset, abnormal findings on brain MRI, and CSF white blood cell count greater than 20 cells/uL. mNEOS scores were calculated for patients with available data in both the imputed and nonimputed data sets, with good and poor 1-year functional status operationalized per the study definitions above. The strength of association between mNEOS score and 1-year functional status was assessed using the Cuzick-Wilcoxon nonparametric test for trend^21^; patients with scores of 0 to 1 and 3 to 4 were pooled together to avoid small groups.^16^

### Statistical Analysis

To prepare the data set for multivariable logistic regression, missing values in 35 predictor variables were imputed using hot-deck imputation with univariate-estimated weighting (eMethods 2 in the Supplement).^22^ Target variables (functional outcome, relapsing disease course) were not imputed. After imputation, worst mRS score in the acute phase was binarized, age was recoded as 4 categorical variables, and 5 variables were removed, resulting in a final set of 33 predictor variables (eTable 1 in the Supplement). Patients with complete data in one or both target variables were entered into the respective logistic regression models, implemented with Statsmodels version 0.12.2 in Python version 3.6 (Python Software Foundation). Two-tailed *P* values less than .05 were regarded as significant. Three approaches were used. (1) Multivariable regression, in which the full available data set was presented in a single model. (2) Bootstrapped multivariable regression, in which random subsets (folds) of the data set were iteratively presented, to assess the stability and variability of the results. Cross-validation was run with 50-50 train-test split in bootstrap (n = 10 000) using class-balanced pseudorandomization (eMethods 2 in the Supplement). Odds ratios (ORs), 95% CIs, and *P* values were derived from the distributions of the regression coefficients over the 10 000 folds. (3) Bootstrapping with cross-validation, in which 20% of cases were held out of training (40%) and testing (40%), to show generalization of the methods to new data and provide a fair assessment of the prediction accuracy that can be reached on unseen patients.

## Results

### Overview of Descriptive Data

**Figure 1.**
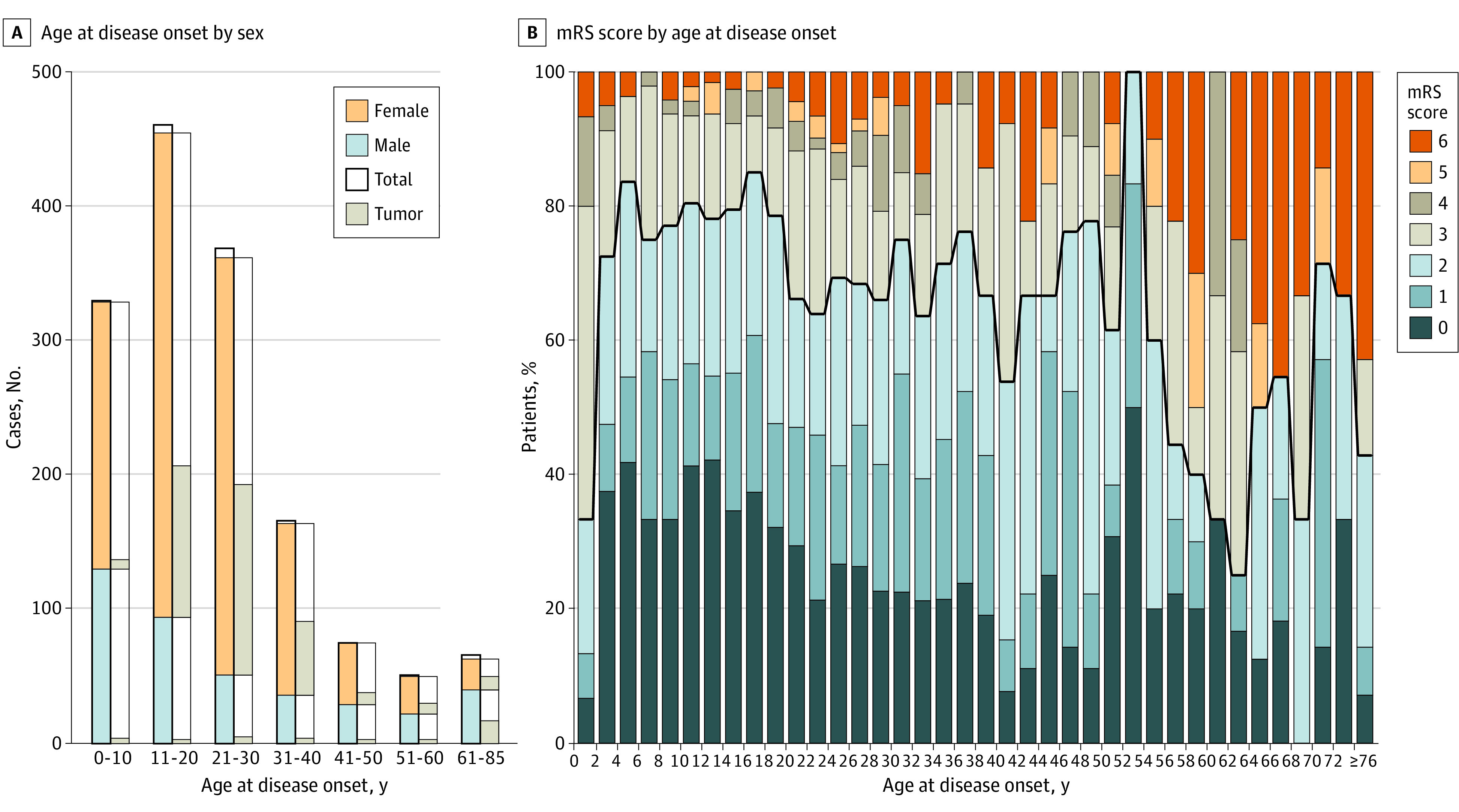
Associations Between Age at Onset and Sex and Modified Rankin Scale (mRS) score A, Patients with reported age at disease onset are displayed (n = 1517). Of these, sex was reported for 1497 of 1517 patients and tumor status for 1491 of 1517 patients. A total of 1160 of 1517 patients (76.5%) had an age at onset of 30 years or younger. Female sex was most pronounced among teenagers and young adults (age, 13 to 30 years; 634 of 763 [83.1%]), less pronounced in children 12 years and younger (237 of 383 [61.9%]), and reversed in adults 50 years and older (55 of 118 [46.6%]). Overall, 389 of 1524 patients (25.6%) had a tumor (ovarian teratoma or other ovarian tumor in 324 of 1524 [21.3%]), and tumors were more frequent in adults (290 of 802 [36.2%]) than children (93 of 698 [13.3%]; χ^2^ = 102.4; *P* < .001), and in female patients (347 of 1097 [31.6%]) than male patients (33 of 395 [8.4%]; χ^2^ = 82.9; *P* < .001). B, Outcome (mRS score at final follow-up) according to age at disease onset in 1269 patients. Patients with onset in infancy (2 years or younger) or older adulthood (65 years or older) tended to have worse outcome.

The literature search retrieved 1570 articles. Of these, 652 articles met the inclusion criteria, reporting a total of 1550 patients with NMDARE (1105 of 1508 [73.3%] female) (eFigures 1 and 2, eAppendix, and eTable 2 in the Supplement). The median (range) age at disease onset was 20 (0-85) years (data available for 1517 patients), with 707 of 1526 patients (46.3%) 18 years or younger at onset. A total of 389 of 1524 patients (25.6%) had a tumor (ovarian teratoma or other ovarian tumor in 324 of 1524 [21.3%]) (Figure 1A). At the nadir of the initial NMDARE presentation, mRS score was 5 in 652 of 1113 patients (58.6%) and 4 in 309 of 1113 patients (27.8%); 488 of 964 (50.6%) required ICU admission.

Immunotherapy was commenced within 30 days from symptom onset in 365 of 728 patients (50.1%) (Table; eTables 3 and 4 in the Supplement). First-line immunotherapy was used in 1395 of 1528 patients (91.3%), most frequently corticosteroids (1205 of 1485 [81.1%]), followed by IVIG (980 of 1476 [66.4%]) and therapeutic apheresis (500 of 1482 [33.7%]). Second-line treatments were used in 486 of 1526 patients (31.8%), after a median (range) of 53 (11-1825) days from symptom onset (data available in 101 of 486 patients); within 30 and 60 days, second-line treatments were used in 22 of 190 patients (11.6%) and in 88 of 159 patients (55.3%), respectively. Only 146 of 1508 patients (9.7%) received maintenance immunotherapy for 6 months or more. Severe adverse events were reported in 43 of 1456 patients receiving immunotherapy (3.0%). A total of 16 of 1456 (1.1%) had a CTCAE grade 3 event, 20 of 1456 (1.4%) had a CTCAE grade 4 event, and 7 of 1456 (0.5%) had a CTCAE grade 5 event (eTable 5 in the Supplement).

**Table.** Descriptive Data on Immunotherapy at First Event and Long-term Outcome

Treatment	No./total No. (%)					*P* value (before vs after 2013)^a^
	Total literature cohort (N = 1550)	Children (≤18 y; n = 707)	Adults (>18 y; n = 819)	Before 2013 (Titulaer et al^1^; n = 387)	After 2013 (Titulaer et al^1^; n = 1163)	
First-line immunotherapy	1395/1528 (91.3)	644/699 (92.1)	728/805 (90.4)	338/386 (87.6)	1057/1142 (92.6)	.003
Corticosteroids^b^	1205/1485 (81.1)	562/678 (82.9)	624/783 (79.7)	298/385 (77.4)	907/1100 (82.5)	.03
IVIG^c^	980/1476 (66.4)	479/678 (70.6)	488/774 (63.0)	232/385 (60.3)	748/1091 (68.6)	.004
TA^d^	500/1482 (33.7)	240/680 (35.3)	245/778 (31.5)	124/385 (32.2)	376/1097 (34.3)	.49
Second-line immunotherapy^e^	486/1526 (31.8)	229/700 (32.7)	245/802 (30.5)	80/384 (20.1)	406/1142 (35.6)	<.001
Rituximab	363/1484 (24.5)	174/680 (25.6)	185/780 (23.7)	52/384 (13.5)	311/1100 (28.3)	<.001
Cyclophosphamide	184/1484 (12.4)	83/680 (12.2)	90/780 (11.5)	42/384 (10.9)	142/1100 (12.9)	.37
Bortezomib^f^	16/1484 (1.1)	9/680 (1.3)	7/780 (0.9)	0/384	16/1100 (1.5)	.02
Tocilizumab^f^	11/1484 (0.7)	2/680 (0.3)	9/780 (1.2)	0/384	11/1100 (1.0)	.08
Intravenous or intrathecal methotrexate^f^	10/1484 (0.7)	6/680 (0.9)	4/780 (0.5)	0/384	10/1100 (0.9)	.07
Other	4/1484 (0.3)	1/680 (0.1)	3/780 (0.4)	0/384	4/1100 (0.4)	.58
Maintenance immunotherapy ≥6 mo	146/1508 (9.7)	76/691 (11.0)	70/793 (8.8)	32/385 (8.3)	114/1123 (10.2)	.32
Mycophenolate mofetil	43/1508 (2.9)	17/700 (2.4)	26/803 (3.2)	5/385 (1.3)	38/1142 (3.3)	.048
Azathioprine	39/1508 (2.6)	23/699 (3.3)	16/805 (2.0)	12/385 (3.1)	27/1143 (2.4)	.45
IVIG	24/1508 (1.6)	9/698 (1.3)	15/803 (1.9)	6/385 (1.6)	18/1140 (1.6)	>.99
Methotrexate	17/1508 (1.1)	12/700 (1.7)	5/806 (0.6)	0/385	17/1145 (1.5)	.01
Corticosteroids	40/1508 (2.7)	23/700 (3.3)	17/806 (2.1)	11/385 (2.9)	29/1145 (2.5)	.71
Rituximab redosing	7/1508 (0.5)	2/698 (0.3)	5/805 (0.6)	1/384 (0.3)	6/1143 (0.5)	.69
Other	4/1508 (0.3)	2/697 (0.3)	2/803 (0.2)	1/384 (0.3)	3/1140 (0.3)	>.99
Time to first immunotherapy ≤30 d	365/728 (50.1)	191/366 (52.2)	173/356 (48.6)	97/178 (54.5)	268/550 (48.7)	.20
Overall immunotherapy combinations						
First-line immunotherapy only	826/1526 (54.1)	375/699 (53.6)	440/803 (54.8)	236/384 (61.5)	590/1142 (51.7)	<.001
First-line + second-line immunotherapy only	421/1526 (27.6)	193/699 (27.6)	216/803 (26.9)	68/384 (17.7)	353/1142 (30.9)	<.001
First-line + second-line + maintenance immunotherapy	60/1526 (3.9)	33/699 (4.7)	27/803 (3.4)	11/384 (2.9)	49/1142 (4.3)	.29
First-line + maintenance immunotherapy only	86/1526 (5.6)	43/699 (6.2)	43/803 (5.4)	21/384 (5.5)	65/1142 (5.7)	>.99
Second-line immunotherapy only	5/1526 (0.3)	3/699 (0.4)	2/803 (0.2)	1/384 (0.3)	4/1142 (0.4)	>.99
No immunotherapy	128/1526 (8.4)	52/699 (7.4)	75/803 (9.3)	47/384 (12.2)	81/1142 (7.1)	.003
First-line immunotherapy combination						
Corticosteroids + IVIG	561/1478 (38.0)	274/677 (40.5)	281/777 (36.2)	126/385 (32.7)	435/1093 (39.8)	.01
Corticosteroids + IVIG + TA	301/1478 (20.4)	153/677 (22.6)	143/777 (18.4)	73/385 (19.0)	228/1093 (20.9)	.46
Corticosteroids only	199/1478 (13.5)	76/677 (11.2)	122/777 (15.7)	67/385 (17.4)	132/1093 (12.1)	.01
Corticosteroids + TA	142/1478 (9.6)	59/677 (8.7)	76/777 (9.8)	32/385 (8.3)	110/1093 (10.1)	.37
IVIG only	86/1478 (5.8)	33/677 (4.9)	52/777 (6.7)	20/385 (5.2)	66/1093 (6.0)	.61
IVIG + TA	35/1478 (2.4)	19/677 (2.8)	15/777 (1.9)	13/385 (3.4)	22/1093 (2.0)	.17
TA only	22/1478 (1.5)	9/677 (1.3)	11/777 (1.4)	6/385 (1.6)	16/1093 (1.5)	.81
No first-line immunotherapy	132/1478 (8.9)	54/677 (8.0)	77/777 (9.9)	48/385 (12.5)	84/1093 (7.7)	.007
Second-line immunotherapy combination						
Rituximab only	244/1484 (16.4)	118/680 (17.4)	125/780 (16.0)	38/384 (9.9)	206/1100 (18.7)	<.001
Rituximab + cyclophosphamide	92/1484 (6.2)	43/680 (6.3)	46/780 (5.9)	14/384 (3.6)	78/1100 (7.1)	.01
Cyclophosphamide only	79/1484 (5.3)	33/680 (4.9)	38/780 (4.9)	28/384 (7.3)	51/1100 (4.6)	.06
Other	30/1484 (2.0)	15/680 (2.2)	15/780 (1.9)	0/384	30/1100 (2.7)	<.001
No second-line immunotherapy	1039/1484 (70.0)	471/680 (69.3)	556/780 (71.3)	304/384 (79.2)	735/1100 (66.8)	<.001
Outcome						
Length of follow-up, mo						
Patients	1059/1550 (68.3)	499/707 (70.6)	554/819 (67.6)	279/387 (72.1)	780/1163 (67.1)	NA
Median	12.0	12.0	12.0	12.0	12.0	.63
Mean	20.7	20.1	21.2	22.2	20.2	
Range	0.5-268.0	0-250.0	0-268.0	0-268.0	0.5-250.0	
Proportion with reported relapse^g^	182/1380 (13.2)	85/634 (13.4)	95/722 (13.2)	53/363 (14.6)	129/1017 (12.7)	.37
mRS score at last follow-up^g^						
Patients	1284/1550 (82.8)	589/707 (83.3)	689/819 (84.1)	338/387 (87.3)	946/1163 (81.3)	NA
Median	2	1	2	2	2	.62
Mean	1.8	1.7	1.9	1.8	1.8	
Range	0-6	0-6	0-6	0-6	0-6	
0 to 2	918/1284 (71.5)	440/589 (74.7)	475/689 (68.9)	237/338 (70.1)	681/946 (72.0)	.53
3 to 5	285/1284 (22.2)	117/589 (19.9)	166/689 (24.1)	82/338 (24.3)	203/946 (21.5)	.29
6	81/1284 (6.3)	32/589 (5.4)	48/689 (7.0)	19/338 (5.6)	62/946 (6.6)	.60
Poor functional outcome at 12 mo^h^	187/582 (32.1)	84/291 (28.9)	100/287 (34.8)	48/146 (32.9)	139/436 (31.9)	.84
Relapsing disease course at ≥24 mo^h^	182/410 (44.3)	85/196 (43.4)	95/210 (45.2)	53/112 (47.3)	129/298 (43.3)	.50

Abbreviations: IVIG, intravenous immunoglobulin; mRS, modified Rankin Scale; NA, not applicable; NMDARE, *N*-methyl-d-aspartate receptor antibody encephalitis; TA, therapeutic apheresis.

^a^ The Fisher exact test was used for nominal data and the Mann-Whitney *U* test for continuous or ordinal data.

^b^ Among patients who received corticosteroids with available data on corticosteroid type in the total literature cohort, most frequently used corticosteroids were methylprednisolone (666 of 776 [85.8%]) and prednisolone (294 of 776 [37.9%]). Among patients who received intravenous methylprednisolone, most frequent dose regimens were 1 g/d for a 3-day to 5-day pulse among adults or 30 mg/kg/d (max 1 g/d) for a 3-day to 5-day pulse in children (358 of 378 [94.7%]); 61 of 662 patients (8.7%) treated with intravenous methylprednisolone received 2 or more courses at first event.

^c^ Among patients who received IVIG in the total literature cohort, most frequent dose regimen in an IVIG course was 2 g/kg in 2 to 5 days (273 of 367 [74.4%]). Among patients who received IVIG, 845 of 970 (87.1%) received 1 course and 125 of 970 (12.9%) received 2 or more courses.

^d^ Among patients treated with therapeutic apheresis in the total literature cohort, therapeutic plasma exchange was used in 478 of 500 patients (95.6%) and immune adsorption in 29 of 500 (5.8%); 468 of 496 (94.4%) received 1 apheresis course and 28 of 496 (5.6%) 2 or more courses.

^e^ When comparing patients who did and did not receive second-line treatments at first event, the proportion with mRS scores of 5 at nadir was 271 of 386 (70.2%) and 374 of 717 (52.2%), respectively; the rate of intensive care unit admission was 62.2% (217 of 349) and 43.8% (265 of 605), respectively. Among patients with relapsing disease with available data (169 of 182), 150 (88.8%) had not received second-line immunotherapy at first event. Of these, 110 had available information on immunotherapy at second event; 29 of 110 received second-line immunotherapy at second event; further events occurred in 2 of 28 (7%). A total of 81 of 110 did not receive second-line immunotherapy at second event; further events occurred in 25 of 78 (32%).

^f^ Among second-line treatments, a subgroup received emerging escalation therapies (30 patients at first event, additional 7 after relapse): intravenous or intrathecal methotrexate (14 patients), intravenous or subcutaneous bortezomib (20 patients), or intravenous tocilizumab (11 patients) (eTable 4 in the Supplement). These patients had severe disease at nadir: 23 of 31 (74%) had decreased level of consciousness, 23 of 33 (70%) had dysautonomia, 30 of 37 (81%) had mRS score of 5, and 21 of 30 (70%) required intensive care unit admission; a median (range) of 4 (2-6) other immunotherapies were administered before methotrexate, bortezomib, or tocilizumab.

^g^ Including all patients with available data (at any follow-up duration).

^h^ Including only patients with available data for ascertainment of good vs poor functional outcome at 12 months or monophasic vs relapsing disease course at 24 months.

The median (range) follow-up duration was 12.0 (0.5-268.0) months (data available in 1059 of 1550). A total of 918 of 1284 patients (71.5%) had good outcome (mRS score of 0 to 2), 285 of 1284 (22.2%) survived with poor outcome (mRS score of 3 to 5), and 81 of 1284 (6.3%) died; poor outcomes and increased death rates were prominent in older patients (Figure 1B). Relapses occurred in 182 of 1380 patients (13.2%; median [range] of 2 [2-9] total disease events per relapsing patient; data available in 163 of 182). First relapse occurred at a median (range) of 12.0 (0.5-30.4) months from onset (data available in 99 of 182). In the subgroup of relapsing patients with mRS score assigned at both first event and first relapse (69 of 182 [37.9%]), disease severity was lower at relapse compared with first event (mean [median; range] mRS score: first event, 4.1 [4; 3-5]; first relapse, 3.6 [3; 3-5]; *z* = −3.6; *P* < .001). Descriptive data on outcome and relapses in relation to immunotherapy are provided in the eResults in the Supplement.

### Factors at First Disease Event Associated With Functional Outcome at 12 Months

**Figure 2.**
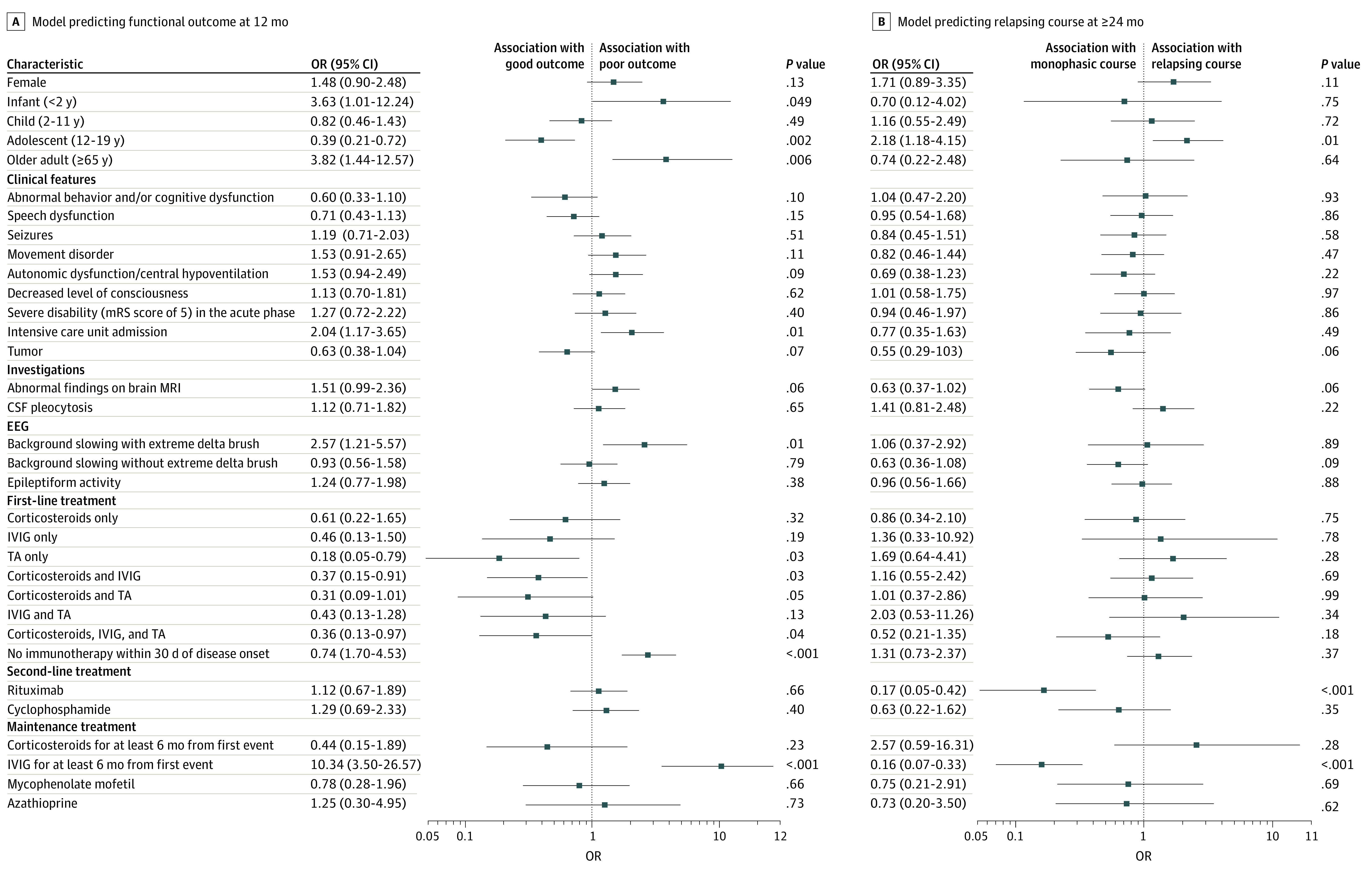
Independent Association of Clinical Characteristics and Treatment Factors With Functional Outcome at 12 Months and Relapsing Disease Course at 24 Months and Later Results from the bootstrapped logistic regression models are displayed. Adjusted odds ratios (ORs), 95% CIs, and *P* values were derived from the distributions of the regression coefficients over 10 000 folds. CSF indicates cerebrospinal fluid; EEG, electroencephalography; IVIG, intravenous immunoglobulin; MRI, magnetic resonance imaging; mRS, modified Rankin Scale; TA, therapeutic apheresis.

A total of 582 patients were included in the model predicting functional outcome at 12 months (187 with poor outcome [mRS score of 3 to 6]). In the bootstrapped logistic regression model (Figure 2A), patient characteristics associated with increased odds of poor outcome were older adult age (65 years or older) at disease onset (OR, 3.82; 95% CI, 1.44-12.57; *P* = .006), infant age (2 years or younger) at disease onset (OR, 3.63; 95% CI, 1.01-12.24; *P* = .049), extreme delta brush pattern on EEG (OR, 2.57; 95% CI, 1.21-5.57; *P* = .01), and ICU admission (OR, 2.04; 95% CI, 1.17-3.65; *P* = .01). Treatment factors associated with poor outcome were use of maintenance IVIG for 6 months or more from first event (OR, 10.34; 95% CI, 3.50-26.57; *P* < .001) and lack of any immunotherapy within 30 days of disease onset (OR, 2.74; 95% CI, 1.70-4.53; *P* < .001). Treatment factors associated with good outcome (ie, decreased OR for poor outcome) were use of therapeutic apheresis alone (OR, 0.18; 95% CI, 0.05-0.79; *P* = .03), use of corticosteroids plus IVIG (OR, 0.37; 95% CI, 0.15-0.91; *P* = .03), and use of corticosteroids plus IVIG plus therapeutic apheresis (OR, 0.36; 95% CI, 0.13-0.97; *P* = .04) at first event. The only patient characteristic associated with good outcome was adolescent age (12 to 19 years) (OR, 0.39; 95% CI, 0.21-0.72; *P* = .002). Results from the nonbootstrapped multivariable regression are provided in eTable 6 in the Supplement. Accuracy for outcome prediction in the cross-validation cohort was 73.3%. In post hoc univariate analysis, receiving earlier initiation of second-line immunotherapy (within 60 days of disease onset; n = 88) was associated with reduced odds of poor outcome compared with later initiation (n = 71) (OR, 0.14; 95% CI, 0.05-0.38; *P* < .001) (eTable 10 in the Supplement).

### Factors at First Disease Event Associated With Relapsing Disease Course

A total of 410 patients were included in the model predicting relapse (182 with relapse). In the bootstrapped logistic regression model (Figure 2B), the only factor significantly associated with increased odds of relapsing disease was adolescent age (OR, 2.18; 95% CI, 1.18-4.15; *P* = .01). Two treatment factors were associated with nonrelapsing disease: use of rituximab as second-line treatment (OR, 0.17; 95% CI, 0.05-0.42; *P* < .001) and use of maintenance IVIG for 6 months or more from first event (OR, 0.16; 95% CI, 0.07-0.33; *P* < .001). Results from the nonbootstrapped multivariable regression are provided in eTable 7 in the Supplement. Accuracy for prediction of relapsing disease course in the cross-validation cohort was 63.4%.

### Changes in Immunotherapy Use and Disease Outcome With Time

**Figure 3.**
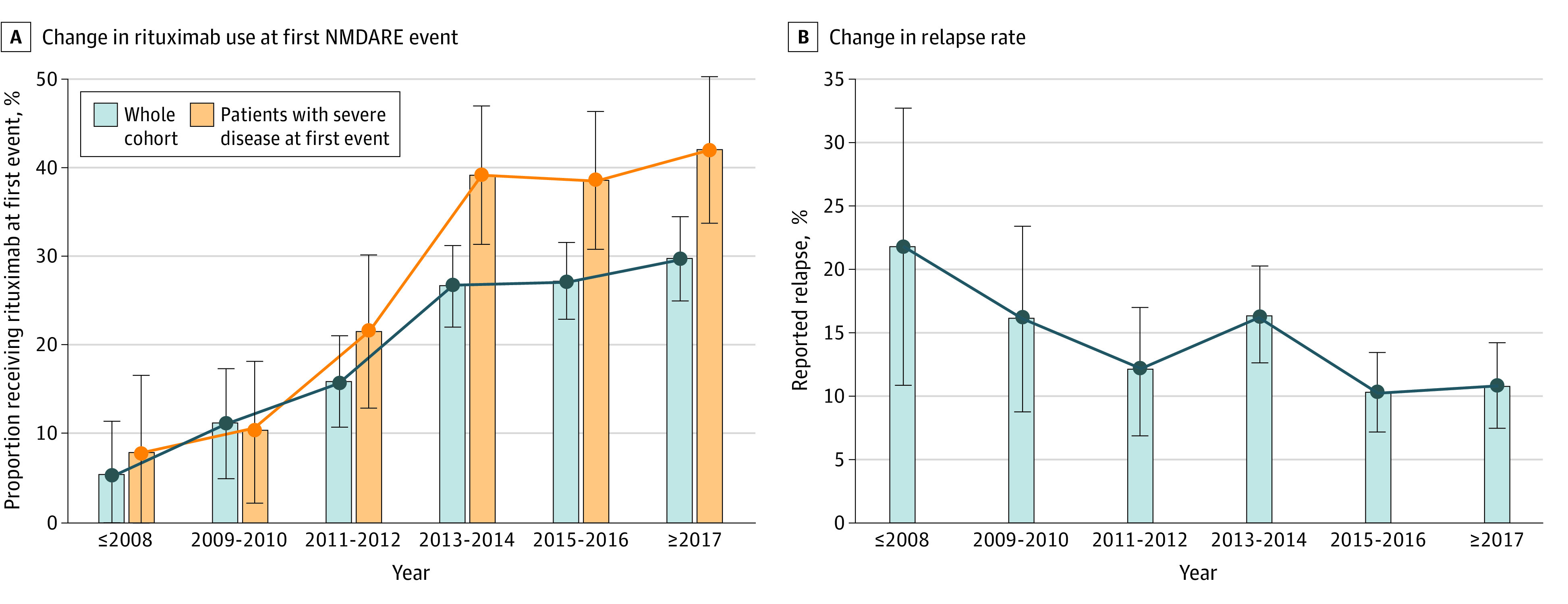
Changes in Rituximab Use at First *N*-Methyl-d-Aspartate Receptor Antibody Encephalitis (NMDARE) Event and Changes in Relapse Rate Over Time Data are displayed over 6 temporal epochs, defined by the year of disease onset, if reported; otherwise, the year of publication was used. A, Proportion of patients receiving rituximab at first event over 6 temporal epochs in the whole cohort (363 of 1484 patients [24.5%]) and in the subset of patients with severe disease (modified Rankin Scale score of 5) at first event (205 of 627 [32.7%]), showing a greater increase in the proportion of rituximab use in patients with severe disease. B, Proportion of patients with reported relapse over 6 temporal epochs (182 of 1380 patients [13.2%]). Error bars represent 95% CIs.

From the early to later epoch (before vs after 2013 [Titulaer et al^1^]), use of first-line immunotherapy increased from 87.6% (338 of 386) to 92.6% (1057 of 1142) (χ^2^ = 9.0; *P* = .003) and use of second-line immunotherapy from 20.1% (80 of 384) to 35.6% (406 of 1142) (χ^2^ = 28.7; *P* < .001). Usage of individual immunotherapies in both epochs (at first event) is detailed in the Table; of note was the increase in rituximab use from 13.5% (52 of 384) to 28.3% (311 of 1100) (χ^2^ = 33.4; *P* < .001), shown over 6 epochs (Figure 3A), and the increase was greater in the subgroup of patients with severe disease (mRS score of 5) at first event. Post hoc analysis revealed a significant association for falling relapse rate over 6 temporal epochs (22% [12 of 55] in 2008 and earlier; 10.9% [35 of 322] in 2017 and later; *z* = 2.72; *P* = .006; Figure 3B); functional outcome did not show any significant association (*z* = 1.76; *P* = .08). The proportion of patients requiring 60 days or more of hospitalization fell from 79.4% (100 of 126) in the early epoch to 59.5% (125 of 210) in the later epoch (*P* < .001) (eTable 2 in the Supplement).

### NEOS Score Validation

**Figure 4.**
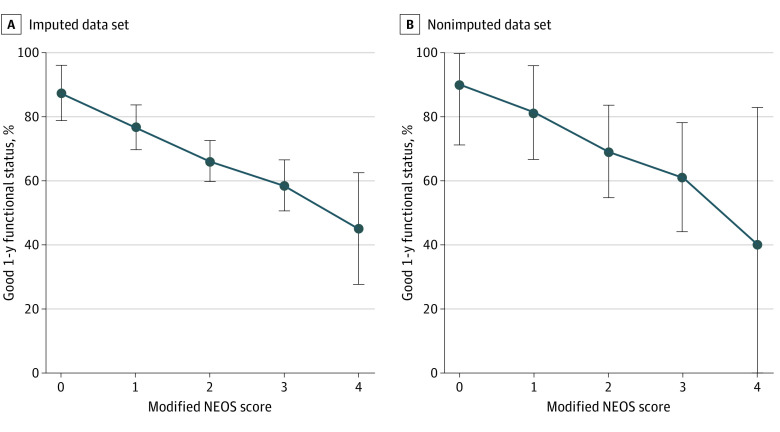
Association Between Modified Anti-NMDAR Encephalitis One-Year Functional Status (NEOS) Score and 1-Year Functional Status Probability of good functional status (modified Rankin Scale score of 0 to 2) at 1 year after disease onset according to the modified NEOS score for all patients with available data in the imputed (n = 582) and nonimputed (n = 112) data sets. Error bars represent 95% CIs.

mNEOS score was assessed in 112 patients from the nonimputed data set (79 with good 1-year functional status) (Figure 4A) and 582 patients from the imputed data set (395 with good 1-year functional status) (Figure 4B). Higher mNEOS score was associated with lower probability of good 1-year functional status in both data sets (nonimputed: score of 0 to 1 points, 31 of 37 [83.8%]; score of 3 to 4 points, 21 of 36 [58.3%]; *z* = −1.96; *P* = .05; imputed: score of 0 to 1 points, 159 of 199 [79.9%]; score of 3 to 4 points, 99 of 176 [56.3%]; *z* = −1.96; *P* = .05).

## Discussion

To our knowledge, this is the most comprehensive evidence synthesis to date focusing on immunotherapy in NMDARE, including individual treatment data from 1550 patients. Factors associated with good functional outcome (mRS score of 0 to 2) were adolescent age and first-line treatment with either therapeutic apheresis alone, corticosteroids with IVIG, or corticosteroids with IVIG and therapeutic apheresis. Factors associated with poor functional outcome (mRS score of 3 to 6) were infant or older adult age, ICU admission, extreme delta brush pattern on EEG, lack of immunotherapy within 30 days of onset, and IVIG treatment for 6 months or more. By contrast, relapsing disease was associated with adolescent age, and monophasic disease was associated with rituximab use or IVIG for 6 months or more.

While our data broadly validate the NEOS score, our different but complimentary approach was to evaluate the association of individual immunotherapies with outcome while controlling for a broad range of demographic and clinical characteristics, including age. Infants (younger than 2 years) and older adults (65 years and older) experienced the worst outcomes of NMDARE, with 3.6-fold and 3.8-fold, respectively, increased odds of poor outcome, while adolescents (aged 12 to 19 years) had 2.6-fold increased odds of good outcome compared with those aged 20 to 65 years at disease onset. This increased vulnerability to insult at the extremes of age may relate to differences in synaptic NMDAR composition in both the developing and aging brain.^23,24^ Among the other clinical characteristics, ICU admission was the only significant independent predictor of poor outcome (2-fold increased odds), consistent with previous studies.^1,16,20^ Abnormal MRI and EEG findings and inflammatory CSF have been variably reported in association with poor outcome of NMDARE.^16,25,26,27,28^ Here, we show that the extreme delta brush pattern, an interictal EEG abnormality highly specific for NMDARE, is independently associated with 2.6-fold increased odds of poor outcome,^29^ as observed, albeit not statistically significant, in the first article describing it.^30^

Although first-line immunotherapy (corticosteroids, IVIG, therapeutic apheresis) is widely used, limited and contrasting evidence exists regarding the best efficacy safety profile of different first-line treatment combinations (eTable 8 in the Supplement).^31,32,33,34,35^ We found that therapeutic apheresis alone (5.6-fold increased odds of good outcome) or first-line treatment options used in combination (2.7-fold increased odds with corticosteroids and IVIG; 2.8-fold increased odds with corticosteroids, IVIG, and therapeutic apheresis) were effective in NMDARE, providing support for a pragmatic approach to selection of first-line therapies guided by adverse effect profile and patient acceptability. Importantly, the only approach to first-line treatment associated with worse outcome was deferral of treatment: lack of immunotherapy within 30 days of disease onset, which occurred in 363 of 728 patients (49.9%) in the total literature review cohort, was associated with 2.7-fold increased odds of poor outcome, consistent with the findings of Titulaer et al^1^ and other studies.^3,5,16,20,25,28,36,37^

Regarding second-line immunotherapies, this evidence synthesis showed a striking association of rituximab administration with monophasic course, with 5.9-fold reduced odds of relapse after 24 months or more follow-up, also confirmed across all the major age groups in univariate analyses (eTable 9 in the Supplement). Our review also showed the emerging use of escalation second-line therapies, such as intravenous/intrathecal methotrexate, subcutaneous/intravenous bortezomib, and intravenous tocilizumab, in a very limited subset of patients (eTable 4 in the Supplement), insufficient for inclusion in our multivariable modeling.^38,39,40,41,42,43,44,45,46,47,48,49,50,51,52,53,54^ Their efficacy and safety in NMDARE warrant further investigation.^55^ We were not able to assess the effect of timing of second-line immunotherapy in our multivariable models; however, post hoc univariate analysis showed that receiving earlier initiation of second-line immunotherapy (within 60 days of disease onset) was associated with 7.1-fold reduced odds of poor outcome compared with later initiation, despite similar disease severity at nadir (eTable 10 in the Supplement).

We found great heterogeneity in the use of and choice of agents for maintenance immunotherapy. In multivariable modeling, IVIG use for 6 months or more was associated with 10.3-fold increased odds of poor functional outcome (poor outcome at last follow-up in 10 of 24 patients [41%] in this group, reported across 17 articles) and yet 6.3-fold decreased odds of relapsing disease. These observations comprise a very small subgroup of cases within the analyzed group. Therefore, considering the potential for publication bias of smaller cohorts favoring more atypical clinical features and immunotherapeutic responsivity, caution should be used in generalizing these findings more widely. A dissociation of functional outcome from relapse risk was also observed in the adolescent group (2.6-fold increased odds of good functional outcome yet 2.2-fold reduced odds of monophasic disease), perhaps reflecting gene-environment interactions operating at this age to promote neuroinflammatory biology.^56^

In our analysis of changes in immunotherapy use with time, the key finding was an increase in the use of rituximab (52 of 384 [13.5%] before 2013 and 311 of 1100 [28.3%] after 2013 [Titulaer et al^1^]), concurrent with a generally falling relapse rate (12 of 55 [22%] in 2008 and earlier and 35 of 322 [10.9%] in 2017 and later) (Figure 3). However, overall functional outcomes did not improve with time. This is consistent with our finding (different from Titulaer et al^1^) that second-line treatments were not significantly associated with functional outcome, but other factors may also be important; patients in the early epoch tended to fit more closely the typical patient with NMDARE, ie, young female adults with ovarian teratomas, whereas in the later epoch, we observed expansion of the recognized phenotype with significantly more men and children, lower tumor prevalence, lower symptom burden, and more atypical presentations, such as demyelination, alongside nonsignificant findings of later hospitalization and less prompt immunotherapy initiation (eTable 2 in the Supplement). These less typical patients might be recognized and treated later and thereby incur worse functional outcomes, despite similar or even less severe disease at nadir. Nevertheless, other metrics do suggest some improvements in management, such as a reduction in the proportion of patients requiring 60 days or more of hospitalization (125 of 210 [59.5%] vs 100 of 126 [79.4%] in the early epoch). Across the whole cohort, immune treatments were well tolerated, with treatment-related severe adverse events (CTCAE grades of 3 to 5) reported in only 3% and tending to occur mostly in bedbound patients in the ICU (eTable 5 in the Supplement),^57^ although adverse events, especially minor ones, were likely greatly underreported and hence not presented in this analysis.

### Limitations

The main limitations of our study include the retrospective nature of the data and inclusion of case reports that are susceptible to biases, such as reporting patients with worse disease or atypical features compared with the general population with NMDARE; this could also be a factor in the observed lack of improvement in functional outcomes across temporal epochs. Since only patients with individually reported treatment data were considered, different to previous reviews,^20,58^ some of the major published cohorts^1^ were not included but were instead used as a comparison (eTable 11 in the Supplement). Although extensively used, the mRS score may be too coarse to capture subtle differences in neuropsychiatric and cognitive symptoms at follow-up.^59^ As mRS score was usually only reported or determined at nadir of the acute illness and final follow-up (rarely at 12 months exactly), our assignments of functional outcome at 12 months were operationalized estimates, justified by the well-documented typical trajectory of recovery in NMDARE.^1,13^ Data collected were inherently limited by heterogeneous availability, hence hot-deck imputation, a robust method for handling missing data in large data sets, was used to enable multivariable analysis.^22^ Although this method generates clinically plausible values (by constraining imputation to values already present in the database), it does not guarantee complete extinction of bias, as implicit assumptions are required in the choice of metric to match donors to recipients. While our multivariable modeling overall accounts for the contribution of each variable to the final predictions, statistical power may be limited when predictor variables are highly correlated (eg, 57.1% of patients receiving cyclophosphamide also received rituximab). We did not design the main model to test for specific interactions between predictors but afterwards evaluated this in a set of additional models and did not find any significant interactions between immunotherapy and severity or demographic factors in their association with functional outcome (eTable 12 in the Supplement).

## Conclusions

This comprehensive and focused literature review on immunotherapy in NMDARE establishes a clear role for early immunotherapy and timely escalation to second-line treatment, particularly with rituximab. Importantly, we found that clinical factors that are associated with functional outcome often are not associated with relapsing disease biology. This should prompt important reevaluation of pragmatic treatment paradigms and physician preference to treat more severely affected patients more aggressively. Additionally, this synthesis of real-world data is an important means to direct relevant clinical and research questions, serving as the basis for the development of evidence-based optimal management and treatment guidelines of NMDARE.

## Notes

Supplement. **eTable 1.** Variables from the literature review data set entered for imputation of missing data. **eTable 2.** Demographic characteristics, clinical data, investigations, disease course, and outcome in the total literature cohort of patients with NMDAR antibody encephalitis. **eTable 3.** First-line immunotherapy at first event of NMDAR antibody encephalitis. **eTable 4.** Demographic characteristics, data in the acute phase of the first event, treatment, disease course, and outcome in the subgroups of patients who received bortezomib, tocilizumab, or intravenous/intrathecal methotrexate. **eTable 5.** Severe immunotherapy-related adverse events. **eTable 6.** Logistic regression model (single iteration) predicting poor functional outcome at 12 months—Statsmodel Logit() report (A) and derived odds ratios (B). **eTable 7.** Logistic regression model (single iteration) predicting relapsing disease course at ≥24 months—Statsmodel Logit() report (A) and derived odds ratios (B). **eTable 8.** Prior published studies comparing effectiveness of immunotherapy combinations. **eTable 9.** Age-stratified associations of rituximab with relapsing disease course. **eTable 10.** Comparison of patients receiving earlier vs later second-line immunotherapy. **eTable 11.** Main factors associated with outcome and disease course in the large cohort by Titulaer et al^1^ and our systematic review. **eTable 12.** Logistic regression models for poor functional outcome at 12 months with interaction terms. **eFigure 1.** PRISMA flow diagram. **eFigure 2.** Number of patients extracted per published report. **eMethods 1.** Additional data on temporal epochs. **eMethods 2.** Statistical appendix. **eResults.** Descriptive data on outcome and relapses in relation to immunotherapy.
eReferences.
**eAppendix.** Reports included in the final data set.
