# Supplementary material for: Use and Safety of Immunotherapeutic Management of N-Methyl-d-Aspartate Receptor Antibody Encephalitis: A Meta-analysis
Source: JAMA Neurol. 2021 Sep 20;78(11):1–12. doi: 10.1001/jamaneurol.2021.3188 (PMC8453367; doi:10.1001/jamaneurol.2021.3188)
Supplement: Supplement. — eTable 1. Variables from the literature review data set entered for imputation of missing data. eTable 2. Demographic characteristics, clinical data, investigations, disease course, and outcome in the total literature cohort of patients with NMDAR antibody encephalitis. eTable 3. First-line immunotherapy at first event of NMDAR antibody encephalitis. eTable 4. Demographic characteristics, data in the acute phase of the first event, treatment, disease course, and outcome in the subgroups of patients who received bortezomib, tocilizumab, or intravenous/intrathecal methotrexate. eTable 5. Severe immunotherapy-related adverse events. eTable 6. Logistic regression model (single iteration) predicting poor functional outcome at 12 months—Statsmodel Logit() report (A) and derived odds ratios (B). eTable 7. Logistic regression model (single iteration) predicting relapsing disease course at ≥24 months—Statsmodel Logit() report (A) and derived odds ratios (B). eTable 8. Prior published studies comparing effectiveness of immunotherapy combinations. eTable 9. Age-stratified associations of rituximab with relapsing disease course. eTable 10. Comparison of patients receiving earlier vs later second-line immunotherapy. eTable 11. Main factors associated with outcome and disease course in the large cohort by Titulaer et al1 and our systematic review. eTable 12. Logistic regression models for poor functional outcome at 12 months with interaction terms. eFigure 1. PRISMA flow diagram. eFigure 2. Number of patients extracted per published report. eMethods 1. Additional data on temporal epochs. eMethods 2. Statistical appendix. eResults. Descriptive data on outcome and relapses in relation to immunotherapy. eReferences. eAppendix. Reports included in the final data set. [file jamaneurol-e213188-s001.pdf]

Supplemental Online Content

Nosadini M, Eyre M, Molteni E, et al; International NMDAR Antibody Encephalitis Consensus Group. Use and safety of immunotherapeutic management of *N*-methyl-D-aspartate receptor antibody encephalitis: a meta-analysis. *JAMA Neurol*. Published online September 20, 2021. doi:10.1001/jamaneurol.2021.3188

eTable 1. Variables from the literature review data set entered for imputation of missing data
eTable 2. Demographic characteristics, clinical data, investigations, disease course, and outcome in the total literature cohort of patients with NMDAR antibody encephalitis
eTable 3. First-line immunotherapy at first event of NMDAR antibody encephalitis
eTable 4. Demographic characteristics, data in the acute phase of the first event, treatment, disease course, and outcome in the subgroups of patients who received bortezomib, tocilizumab, or intravenous/intrathecal methotrexate
eTable 5. Severe immunotherapy-related adverse events
eTable 6. Logistic regression model (single iteration) predicting poor functional outcome at 12 months—Statsmodel Logit() report (A) and derived odds ratios (B)
eTable 7. Logistic regression model (single iteration) predicting relapsing disease course at ≥24 months—Statsmodel Logit() report (A) and derived odds ratios (B)
eTable 8. Prior published studies comparing effectiveness of immunotherapy combinations
eTable 9. Age-stratified associations of rituximab with relapsing disease course
eTable 10. Comparison of patients receiving earlier vs later second-line immunotherapy
eTable 11. Main factors associated with outcome and disease course in the large cohort by Titulaer et al[1] and our systematic review
eTable 12. Logistic regression models for poor functional outcome at 12 months with interaction terms
eFigure 1. PRISMA flow diagram
eFigure 2. Number of patients extracted per published report
eMethods 1. Additional data on temporal epochs
eMethods 2. Statistical appendix
eResults. Descriptive data on outcome and relapses in relation to immunotherapy
eReferences.
eAppendix. Reports included in the final data set

This supplementary material has been provided by the authors to give readers additional information about their work.

© 2021 Nosadini M et al. *JAMA Neurology*.

eTable 1. Variables from the literature review data set entered for imputation of missing data

VARIABLE	PROPORTION OF CASES IMPUTED*	INCLUDED IN REGRESSION MODELS	NOTES
**Demographics**			
Sex	1.80%	Yes	
Age	0.90%	Yes	Recoded as categorical variable after imputaton
**Clinical features**			
Abnormal behaviour and/or cognitive dysfunction	5.62%	Yes	
Speech dysfunction	11.25%	Yes	
Seizures	8.21%	Yes	
Movement disorder	10.01%	Yes	
Autonomic dysfunction/central hypoventilation	10.69%	Yes	
Decreased level of consciousness	11.02%	Yes	
Worst mRS in the acute phase	24.63%	Yes	Binarised after imputation (mRS 5 versus mRS <5)
Intensive care unit admission	33.86%	Yes	
Tumour	0.56%	Yes	
**Investigations**			
Abnormal MRI brain	28.68%	Yes	
CSF pleocytosis (binary)	36.22%	Yes	
CSF WBC count (continuous)	61.53%	No	Included only for NEOS validation
EEG: focal or diffuse slow or disorganised activity	45.44%	Yes	
EEG: extreme delta brush	45.33%	Yes	
EEG: epileptiform activity**	45.33%	Yes	
**First-line treatment**			
Corticosteroids only	6.41%	Yes	
IVIG only	6.41%	Yes	
Therapeutic apheresis only	6.41%	Yes	
Corticosteroids and IVIG	6.41%	Yes	
Corticosteroids and Therapeutic apheresis	6.41%	Yes	
IVIG and Therapeutic apheresis	6.41%	Yes	
Corticosteroids, IVIG and Therapeutic apheresis	6.41%	Yes	
Days from symptom onset to immunotherapy	66.93%	No	Already included in binarised form
No immunotherapy within 30 days of disease onset^	47.69%	Yes	
**Second-line treatment**			
Rituximab	5.40%	Yes	
Cyclophosphamide	5.29%	Yes	
Bortezomib	1.69%	No	Excluded due to low occurrence (9/889 cases, 1.01%)
Tocilizumab	1.69%	No	Excluded due to low occurrence (4/889 cases, 0.45%)
**Maintenance treatment**			
Corticosteroids for at least 6 months from first event	1.46%	Yes	
IVIG for at least 6 months from first event	1.57%	Yes	
Mycophenolate mofetil	1.80%	Yes	
Azathioprine	1.57%	Yes	
Methotrexate	1.46%	No	Excluded due to low occurrence (5/889 cases, 0.56%)

© 2021 Nosadini M et al. *JAMA Neurology*.

**Mean**	**16.07%**		

eTable 1. Variables from the literature review dataset entered for imputation of missing data.
Legend: CSF: cerebrospinal fluid; EEG: electroencephalography; IVIG: intravenous immunoglobulin; MRI: magnetic resonance imaging; mRS: modified Rankin Scale.
*Number of cases with missing data per variable, divided by the total 889 cases included in one or both regression models.
**Defined as the presence of interictal epileptiform discharges, electrical seizures and/or electrical status epilepticus
^Data on time to first immunotherapy was available in 505/1551 patients. 287/505 patients did not receive immunotherapy within 30 days from symptom onset; these also included 128 patients who did not receive any immunotherapy at first event.

© 2021 Nosadini M et al. *JAMA Neurology*.

eTable 2. Demographic characteristics, clinical data, investigations, disease course, and outcome in the total literature cohort of patients with NMDAR antibody encephalitis

	Total literature cohort (*N*=1550)	Pre-Titulaer et al. 2013[1] (*N*=387)	Post-Titulaer et al. 2013[1] (*N*=1163)	*P*
**ARTICLE DATA**				
**Total number of articles and year of publication**	652	228	424	-
Year of publication 2007-2010	60/652 (9.2%)	60/228 (26.3%)	0/424 (0%)	<0.001
Year of publication 2011-2014	254/652 (39.0%)	157/228 (68.9%)	97/424 (22.9%)	<0.001
Year of publication 2015-2018	338/652 (51.8%)	11/228 (4.8%)	327/424 (77.1%)	<0.001
**Full text/Abstract**				
Full text	635/652 (97.4%)	215/228 (94.3%)	420/424 (99.1%)	<0.001
Abstract only	17/652 (2.6%)	13/228 (5.7%)	4/424 (0.9%)	<0.001
**Language (full text)**				
English	608/635 (95.7%)	202/215 (94.0%)	406/420 (96.7%)	0.14
Spanish	19/635 (3.0%)	8/215 (3.7%)	11/420 (2.6%)	0.47
French	7/635 (1.1%)	4/215 (1.9%)	3/420 (0.7%)	0.23
Portuguese	1/635 (0.2%)	1/215 (0.5%)	0/420 (0%)	0.34
**Number of patients per article**	Median 1, mean 2.4, range 1-48 (d.a.: 652/652)	Median 1, mean 1.7, range 1-15 (d.a.: 228/228)	Median 1, mean 2.7, range 1-48 (d.a.: 424/424)	0.22
Articles reporting 1 patient with NMDARE	479/652 (73.5%)	174/228 (76.3%)	305/424 (71.9%)	0.26
Articles reporting 2 patients with NMDARE	64/652 (9.8%)	25/228 (11.0%)	39/424 (9.2%)	0.49
Articles reporting 3-10 patients with NMDARE	84/652 (12.9%)	27/228 (11.8%)	57/424 (13.4%)	0.62
Articles reporting 11-20 patients with NMDARE	17/652 (2.6%)	2/228 (0.9%)	15/424 (3.6%)	0.04
Articles reporting >20 patients with NMDARE	8/652 (1.2%)	0/228 (0%)	8/424 (1.9%)	0.06
**DEMOGRAPHICS**				
**Total number of patients**	1550	387	1163	-
**Proportion of females**	1105/1508 (73.3%)	310/387 (80.1%)	795/1121 (70.9%)	<0.001
**Proportion of children (≤18 years)**	707/1526 (46.3%)	161/387 (41.6%)	546/1139 (47.9%)	0.03
**Age at onset of NMDARE (years)**	Median 20, mean 23.0, range 0-85 (d.a.: 1517/1550)	Median 21, mean 22.5, range 2-84 (d.a.: 386/387)	Median 19, mean 23.1, range 0-85 (d.a.: 1131/1163)	0.27
**CLINICAL DATA AT FIRST EVENT OF NMDARE**				
**Days from symptom onset to hospitalization**	Median 10, mean 30.3, range 0-2190 (d.a.: 452/1550)	Median 7, mean 20.0, range 1-274 (d.a.: 142/387)	Median 10, mean 35.1, range 0-2190 (d.a.: 310/1163)	0.51
≤7 days	213/452 (47.1%)	72/142 (50.7%)	141/310 (45.5%)	0.31
≤10 days	252/452 (55.8%)	85/142 (59.9%)	167/310 (53.9%)	0.26
≤21 days	347/452 (76.8%)	113/142 (79.6%)	234/310 (75.5%)	0.40
**Clinical symptoms in the acute phase: main symptoms**				
Abnormal (psychiatric) behaviour or cognitive dysfunction	1177/1409 (83.5%)	328/366 (89.6%)	849/1043 (81.4%)	<0.001
Speech dysfunction (pressured speech, verbal reduction, mutism)	521/1341 (38.9%)	183/356 (51.4%)	338/985 (34.3%)	<0.001
Seizures	944/1382 (68.3%)	255/365 (69.9%)	689/1017 (67.7%)	0.47
Movement disorder, dyskinesias, or rigidity/abnormal postures	855/1357 (63.0%)	267/359 (74.4%)	588/998 (58.9%)	<0.001
Decreased level of consciousness	744/1343 (55.4%)	257/358 (71.8%)	487/985 (49.4%)	<0.001

© 2021 Nosadini M et al. *JAMA Neurology*.

Autonomic dysfunction or central hypoventilation	583/1351 (43.2%)	222/366 (60.7%)	361/985 (36.6%)	<0.001
**Clinical symptoms in the acute phase: other symptoms**				
Prodromal flu-like symptoms	233/1361 (17.1%)	108/352 (30.7%)	125/1009 (12.4%)	<0.001
Sleep-wake cycle disturbances	223/1359 (16.4%)	91/351 (25.9%)	132/1008 (13.1%)	<0.001
Memory disturbances	234/1376 (17.0%)	70/355 (19.7%)	164/1021 (16.1%)	0.12
**Worst mRS in the acute phase**				
mRS 2	8/1113 (0.7%)	0/320 (0.0%)	8/793 (1.0%)	0.11
mRS 3	144/1113 (12.9%)	27/320 (8.4%)	117/793 (14.8%)	0.004
mRS 4	309/1113 (27.8%)	89/320 (27.8%)	220/793 (27.7%)	1.00
mRS 5	652/1113 (58.6%)	204/320 (63.7%)	448/793 (56.5%)	0.03
**Admission to the intensive care unit**	488/964 (50.6%)	165/300 (55.0%)	323/664 (48.6%)	0.07
**Mechanical ventilation**	302/834 (36.2%)	104/268 (38.8%)	198/566 (35.0%)	0.32
**Length of hospitalisation ≥60 days**	225/336 (67.0%)	100/126 (79.4%)	125/210 (59.5%)	<0.001
**Associated tumour***	389/1524 (25.6%)	132/377 (35.0%)	257/1147 (22.4%)	<0.001
Ovarian teratoma	312/1524 (20.5%)	114/377 (30.2%)	198/1147 (17.3%)	<0.001
Other ovarian tumour	12/1524 (0.8%)	7/377 (1.9%)	5/1147 (0.4%)	0.01
Other tumour (extra-ovarian tumour)	66/1524 (4.3%)	11/377 (2.9%)	54/1147 (4.7%)	0.14
**Preceding/concomitant/subsequent associated demyelinating CNS disease**	54/1517 (3.6%)	7/373 (1.9%)	47/1144 (4.1%)	0.05
**INVESTIGATIONS AT FIRST EVENT OF NMDARE**				
**Abnormal brain MRI**	434/1069 (40.6%)	159/328 (48.5%)	275/741 (37.1%)	<0.001
**Abnormal EEG****	725/855 (84.8%)	244/280 (87.1%)	481/575 (83.7%)	0.19
Focal or diffuse slow or disorganised activity	594/822 (72.3%)	209/280 (74.6%)	385/542 (71.0%)	0.29
Epileptic activity (epileptic discharges)	244/829 (29.4%)	95/281 (33.8%)	149/548 (27.2%)	0.05
Seizures recorded	132/829 (15.9%)	65/281 (23.1%)	67/548 (12.2%)	<0.001
Status epilepticus recorded	43/827 (5.2%)	24/280 (8.6%)	19/547 (3.5%)	0.003
Extreme delta brush	56/827 (6.8%)	1/280 (0.4%)	55/547 (10.1%)	<0.001
Periodic lateralized epileptic discharges (PLEDs)	13/827 (1.6%)	7/280 (2.5%)	6/547 (1.1%)	0.14
**Abnormal CSF*****	692/924 (74.9%)	246/296 (83.1%)	446/628 (71.0%)	<0.001
Pleocytosis >4 cells/uL	618/911 (67.8%)	225/296 (76.0%)	393/615 (63.9%)	<0.001
Hyperproteinorrachia >50 mg/dL	189/873 (21.6%)	55/293 (18.8%)	134/580 (23.1%)	0.16
Positive oligoclonal bands	223/356 (62.6%)	99/143 (69.2%)	124/213 (58.2%)	0.04
**NMDAR-antibodies**				
Positive in serum	702/796 (88.2%)	206/226 (91.2%)	496/570 (87.0%)	0.11
Positive in CSF	973/986 (98.7%)	252/255 (98.8%)	721/731 (98.6%)	1.00

eTable 2. Demographics, clinical data, investigations, disease course and outcome in the total literature cohort of patients with NMDAR-antibody encephalitis and according to pre and post-Titulaer *et al.*, 2013[1] epoch.
CNS: central nervous system; CSF: cerebrospinal fluid; d.a.: data available; EEG: electroencephalography; MRI: magnetic resonance imaging; mRS: modified Rankin Scale; NMDARE: NMDAR-antibody encephalitis. The Fisher exact test was used for nominal data and the Mann-Whitney U test for continuous or ordinal data.
*One patient had both ovarian teratoma and extra-ovarian tumour.
Among 66 patients with extra-ovarian tumours, 49.2% (32/65) were females, and 18.2 (12/66) were in paediatric age.
Most frequent extra-ovarian tumour locations were: lung (11/66), mediastinum (5/66), brain, uterus, lymphoma (4/66 each). Less common tumour locations were: adrenal glands, breast, fallopian tubes, pancreas, testicle (3/66 each); thymus, kidney, liver, prostate (2/66 each); abdomen, stomach, bladder, gluteal muscle, heart, inguinal region, parotid glands, perineum, retroperitoneum, tonsils, acute myeloid leukemia, neuroendocrine tumours (1/66 each); tumour location was not available in 3/66.
**Abnormal EEG was considered the presence of focal or diffuse slow or disorganised activity, epileptic activity, or extreme delta brush.[2]
***Abnormal CSF was considered CSF with pleocytosis or oligoclonal bands.[2]

© 2021 Nosadini M et al. *JAMA Neurology*.

eTable 3. First-line immunotherapy at first event of NMDAR antibody encephalitis

DATA ON FIRST-LINE IMMUNOTHERAPY AT FIRST EVENT OF NMDARE IN THE TOTAL LITERATURE COHORT (*N*=1550)	
**FIRST-LINE IMMUNOTHERAPY**	**1395/1528 (91.3%)**
**Corticosteroids**	**1205/1485 (81.1%)**
*Type of corticosteroids (regardless of the route of administration)**	
Methylprednisolone	666/776 (85.8%)
Prednisone	294/776 (37.9%)
Other (dexamethasone, ACTH, hydrocortisone, bethamethasone)	29/776 (3.7%)
*Route of administration (regardless of the type of corticosteroid)**	
Intravenous	814/911 (89.4%)
Oral	315/911 (34.6%)
Intramuscular or intrathecal	7/911 (0.8%)
**Intravenous immunoglobulin**	**980/1476 (66.4%)**
*Number of courses**	
1 course	845/970 (87.1%)
≥2 courses	125/970 (12.9%)
**Therapeutic apheresis**	**500/1482 (33.7%)**
*Type of apheresis**	
Plasmapheresis	478/500 (95.6%)
Immune adsorption	29/500 (5.8%)
*Number of courses and total number of exchanges**	
1 course	468/496 (94.4%)
≥2 courses	28/496 (5.6%)
Total number of exchanges	Median 5, mean 6.6, range 1-23 (d.a.: 196/500)
**Total number of first-line immunotherapies**	
0	132/1478 (8.9%)
1	307/1478 (20.8%)
2	738/1478 (49.9%)
3	301/1478 (20.4%)

eTable 3. First-line immunotherapy at first event of NMDAR-antibody encephalitis.
Legend: ACTH: adrenocorticotropic hormone; d.a.: available data; n.a.: not available.
*Denominators refer to the total number of patients who received the treatment, with available data (i.e. total number of patients who received corticosteroids, with available data on corticosteroid type).

© 2021 Nosadini M et al. *JAMA Neurology*.

eTable 4. Demographic characteristics, data in the acute phase of the first event, treatment, disease course, and outcome in the subgroups of patients who received bortezomib, tocilizumab, or intravenous/intrathecal methotrexate

DATA ON LITERATURE PATIENTS WITH NMDARE WHO RECEIVED BORTEZOMIB, TOCILIZUMAB OR INTRAVENOUS/INTRATHECAL METHOTREXATE AT FIRST DISEASE EVENT OR AFTER RELAPSE	Patients treated with intravenous or intrathecal Methotrexate^ (n=14)	Patients treated with intravenous or subcutaneous Bortezomib^^ (n=20)	Patients treated with intravenous Tocilizumab^^^ (n=11)
**ARTICLE DATA AND DEMOGRAPHICS**			
**Total articles (year of publication)**	9 (2013-2018)	9 (2016-2018)	3 (2017-2018)
**Proportion of females**	10/13 (76.9%)	16/19 (84.2%)	8/11 (72.7%)
**Proportion of children (≤18 years)**	9/14 (64.3%)	1/20 (5%)	2/11 (18.2%)
**Age at onset (years)**	Median 16, mean 17.5, range 7-31 (d.a.: 13/14)	Median 27, mean 33, range 17-61 (d.a.: 20/20)	Median 26, mean 31.6, range 17-58 (d.a.: 11/11)
**CLINICAL DATA AT FIRST EVENT OF NMDARE**			
**Main clinical symptoms in the acute phase**			
Abnormal (psychiatric) behaviour or cognitive dysfunction	12/13 (92.3%)	16/18 (88.9%)	11/11 (100%)
Speech dysfunction (pressured speech, verbal reduction, mutism)	3/13 (23.1%)	6/15 (40%)	8/11 (72.7%)
Seizures	11/13 (84.6%)	17/19 (89.5%)	11/11 (100%)
Movement disorder, dyskinesias, or rigidity/abnormal postures	9/13 (69.2%)	13/17 (76.5%)	10/11 (90.9%)
Decreased level of consciousness	9/13 (69.2%)	10/15 (66.7%)	10/11 (90.9%)
Autonomic dysfunction or central hypoventilation	8/13 (61.5%)	11/17 (64.7%)	10/11 (90.9%)
**mRS in the acute phase**	Median 5, mean 4.5, range 3-5 (d.a.: 14/14)	Median 5, mean 4.9, range 4-5 (d.a.: 20/20)	Median 5, mean 4.8, range 4-5 (d.a.: 11/11)
mRS 2	0/14 (0%)	0/20 (0%)	0/11 (0%)
mRS 3	2/14 (14.3%)	0/20 (0%)	0/11 (0%)
mRS 4	3/14 (21.4%)	2/20 (10%)	2/11 (18.2%)
mRS 5	9/14 (64.3%)	18/20 (90%)	9/11 (81.8%)
**Admission to the intensive care unit**	7/12 (58.3%)	14/19 (73.7%)	3/6 (50%)
**Mechanical ventilation**	7/12 (58.3%)	13/17 (76.5%)	3/4 (75%)
**Length of hospitalization (days)**	Median 328.5, mean 384.2, range 120-760 (d.a.: 4/14)	Median 339, mean 342.7, range 243-450 (d.a.: 4/20)	313 (d.a.: 1/11)
**Associated tumour**	4/14 (28.6%)	7/20 (35%)	6/11 (54.5%)
**Association with demyelinating CNS disease (preceding/concomitant/subsequent)**	0/14 (0%)	0/20 (0%)	0/11 (0%)
**INVESTIGATIONS**			
**Abnormal brain MRI**	4/10 (40%)	7/12 (58.3%)	8/11 (72.7%)
**Abnormal EEG***	9/11 (81.8%)	9/9 (100%)	11/11 (100%)
**Abnormal CSF****	7/11 (63.6%)	9/11 (81.8%)	9/11 (81.8%)
**NMDAR-antibodies in serum**	6/7 (85.7%)	15/15 (100%)	6/6 (100%)
**NMDAR-antibodies in CSF**	13/13 (100%)	17/17 (100%)	6/6 (100%)
**IMMUNOTHERAPY AT FIRST EVENT**			
**Any immunotherapy**	14/14 (100%)	19/20 (95%)	11/11 (100%)
**First-line immunotherapy**	14/14 (100%)	19/20 (95%)	11/11 (100%)
Corticosteroids	14/14 (100%)	16/20 (80%)	10/11 (90.9%)
Intravenous immunoglobulin	10/14 (71.4%)	17/20 (85%)	11/11 (100%)
Therapeutic apheresis	6/14 (42.8%)	13/20 (65%)	4/11 (36.4%)

© 2021 Nosadini M et al. *JAMA Neurology*.

**Second-line immunotherapy**	12/14 (85.7%)	16/20 (80%)	11/11 (100%)
Rituximab	9/14 (64.3%)	16/20 (80%)	11/11 (100%)
Cyclophosphamide	5/14 (35.7%)	7/20 (35%)	5/11 (45.4%)
Intravenous or intrathecal Methotrexate	10/14 (71.4%)	1/20 (5%)	1/11 (9.1%)
Bortezomib	1/14 (7.1%)	16/20 (80%)	7/11 (63.6%)
Tocilizumab	1/14 (7.1%)	7/20 (35%)	11/11 (100%)
Other	1/14 (7.1%)	1/20 (5%)	3/11 (27.3%)
**Long-term immune modulation ≥6 months**	2/14 (14.3%)	10/20 (50%)	6/11 (54.5%)
Mycophenolate mofetil	1/14 (7.1%)	0/20 (0%)	0/11 (0%)
Azathioprine	0/14 (0%)	0/20 (0%)	0/11 (0%)
Intravenous immunoglobulin	0/14 (0%)	5/20 (25%)	5/11 (45.4%)
Methotrexate	0/14 (0%)	4/20 (20%)	0/11 (0%)
Corticosteroids	0/14 (0%)	0/20 (0%)	0/11 (0%)
Rituximab re-dosing	1/14 (7.1%)	6/20 (30%)	6/11 (54.5%)
Other	0/14 (0%)	0/20 (0%)	0/11 (0%)
**Time from onset to first immunotherapy (days)**	Median 10, mean 15.2, range 5-35 (d.a.: 9/14)	Median 13, mean 20.5, range 7-70 (d.a.: 8/20)	Median 10, mean 20, range 7-70 (d.a.: 6/11)
**First immunotherapy ≤30 days from onset**	8/10 (80%)	8/10 (80%)	5/6 (83.3%)
**Number of different immunotherapies received before Bortezomib / Tocilizumab / intravenous or intrathecal Methotrexate**	Median 3.5, mean 3.6, range 2-6 (d.a.: 14/14)	Median 4, mean 4.2, range 2-6 (d.a.: 20/20)	Median 3, mean 3.4, range 2-4 (d.a.: 11/11)
**RELAPSES AND OUTCOME AT FOLLOW-UP**			
**Length of follow-up (months)**	Median 12, mean 12.9, range 4-25 (d.a.: 10/14)	Median 13, mean 35.1, range 4-268 (d.a.: 17/20)	Median 5.5, mean 9, range 4-20 (d.a.: 10/11)
**Proportion of patients who relapsed**	3/14 (21.4%)	4/15 (20.7%)	0/6 (0%)
**mRS at last follow-up**	Median 1, mean 2.2, range 0-6 (d.a.: 13/14)	Median 3, mean 3.1, range 0-5 (d.a.: 20/20)	Median 5, mean 4.6, range 2-5 (d.a.: 11/11)
mRS 0-1	7/13 (53.8%)	4/20 (20%)	0/11 (0%)
mRS 2-3	3/13 (23.1%)	8/20 (40%)	1/11 (9.1%)
mRS 4-5	0/13 (0%)	8/20 (40%)	10/11 (90.9%)
mRS 6	3/13 (23.1%)	0/20 (0%)	0/11 (0%)

eTable 4. Demographics, data in the acute phase of the first event, treatment, disease course and outcome in the subgroups of literature patients who received bortezomib, tocilizumab or intravenous/intrathecal methotrexate for NMDAR-antibody encephalitis, at onset or after relapse (total 37 patients; 11/37 children; 7/37 received both bortezomib and tocilizumab).
CNS: central nervous system; CSF: cerebrospinal fluid; d.a.: data available; EEG: electroencephalography; MRI: magnetic resonance imaging; mRS: modified Rankin Scale; NMDARE: NMDAR-antibody encephalitis.
*Abnormal EEG was considered the presence of focal or diffuse slow or disorganised activity, epileptic activity, or extreme delta brush.[2]
**Abnormal CSF was considered CSF with pleocytosis or oligoclonal bands.[2]

^*Intravenous or intrathecal methotrexate*. The use of intravenous or intrathecal methotrexate as a second-line treatment was reported in 14 patients (median age 16 years, range 7-31), described in 9 articles published between 2013 and 2018. Disease severity in this subgroup was slightly higher than in the total cohort: 69.2% (9/13) had decreased level of consciousness, 61.5% (8/13) had dysautonomias, 64.3% (9/14) had mRS 5, and 58.3% (7/12) required ICU admission. In 71.4% (10/14), intravenous/intrathecal methotrexate was administered at first disease event, and in the remaining 28.6% (4/14) only after relapse. A median of 3.5 other immunotherapies were administered before intravenous/intrathecal methotrexate. Two of the patients in this subgroup experience adverse reactions, although not directly attributable to intravenous/intrathecal methotrexate: 1 had lymphopenia secondary to overall immunotherapy, and another 1 died due to septic shock after receiving cyclophosphamide. While most patients had good outcome (mRS 0-1 in 7/13, 53.8%), 23.1% died (3/13).

^^*Bortezomib*. The use of subcutaneous or intravenous bortezomib was reported in 20 patients, mostly adults (median age 27 years, range 17-61), described in 9 articles published between 2016 and 2018. Disease severity in this subgroup was higher than in the total cohort: 66.7% (10/15) of patients had

© 2021 Nosadini M et al. *JAMA Neurology*.

decreased level of consciousness, 64.7% (11/17) had dysautonomias, 90% (18/20) had mRS 5, and 73.7% (14/19) were admitted to the ICU. In 80% (16/20), bortezomib was administered at first disease event, and in the remaining 20% (4/20) only after relapse. A median of 4 other immunotherapies were administered before bortezomib. 7 patients in this subgroup experienced adverse reactions: 1 had lymphopenia secondary to overall immunotherapy, and the remaining 6 had side effects to bortezomib (infections in 2/6, neurotropenia in 5/6, anemia in 4/6). While none of the patients in this subgroup died, rate of good neurological outcome was low (mRS 0-1: 4/20, 20%).

^^^*Tocilizumab*. The use of intravenous tocilizumab was reported 11 patients, mostly adults (median age 26 years, range 17-58), described in 3 articles published between 2017 and 2018. Similar to the bortezomib group, disease severity in this subgroup was higher than in the total cohort: 90.9% (10/11) had decreased level of consciousness, 90.9% (10/11) had dysautonomias, 81.8% (9/11) had mRS 5, and 50% (3/6) were admitted to the ICU. In all cases tocilizumab was administered at first disease event, and none of the patients with available data relapsed (0/6, 0%). A median of 3 other immunotherapies were administered before tocilizumab. 6 patients in this subgroup experienced adverse reactions, although not directly attributable to tocilizumab: 1 had lymphopenia secondary to overall immunotherapy, and the remaining 5 had side effects to bortezomib (infections, neutropenia, anemia). While none of the patients in this subgroup died, 90.9% had poor neurological outcome (mRS 4-5: 10/11).

© 2021 Nosadini M et al. *JAMA Neurology*.

eTable 5. Severe immunotherapy-related adverse events

A. Type of treatment-related severe adverse events (CTCAE grades 3-5)	Number of patients
Infection	22
Hypotension	9
Neutropenia	7
Anaphylaxis or allergic reactions	6
Anaemia	4
Thrombosis or thromboembolism	3
Intestinal perforation	2
Hyperpyrexia	2
Disseminated intravascular coagulation	1
Hypothermia, hypersalivation, and cardiac arrhythmias	1
Elevated serum creatine kinase	1
Hemoptysis secondary to hypofibrinogenemia	1
Shock	1
Flushing, diaphoresis, coarse breath sounds	1
Diarrhea	1
**B. Immunotherapy to which severe adverse events (CTCAE grades 3-5) were attributed to***	**Number of patients (%)**
Therapeutic apheresis	12/47 (25.6%)
Rituximab	10/47 (21.3%)
Intravenous immunoglobulin	4/47 (8.5%)
Cyclophosphamide	2/47 (4.3%)
Corticosteroids	2/47 (4.3%)
Bortezomib	2/47 (4.3%)
Treatment combinations	6/47 (12.8%)
Not available	9/47 (19.1%)

eTable 5. Adverse events to immunotherapy were categorised according to the National Institutes of Health Common Terminology Criteria for Adverse Events (CTCAE) v5.0, with a focus on severe events: grade 3 (severe or medically significant but not immediately life-threatening; hospitalisation or prolongation of hospitalisation indicated; disabling; limiting self-care activities of daily living), grade 4 (life-threatening consequences; urgent intervention indicated) and grade 5 (death related to adverse event).
Severe immunotherapy-related adverse events (CTCAE v5.0 grades 3-5) occurred in 47 patients. Multiple adverse reactions could occur in an individual patient. Of the 47 patients with severe adverse events to immunotherapy, 63.8% (30/47) were treated with second-line immunotherapy.
Among 486 patients who received second-line treatments, 415/486 patients received rituximab only (244/415), cyclophosphamide only (79/415), or rituximab and cyclophosphamide only (92/415) (excluding patients who received additional second-line treatments beside rituximab and/or cyclophosphamide). Among these subgroups, rates of adverse events to immunotherapy (any immunotherapy) were 4.3% (10/231), 2.7% (2/74), and 11.2% (10/89), respectively (P=0.03, 3x2 Chi square).
Most patients suffering severe adverse events had severe disease: 91.4% (32/35) had mRS 5 at nadir (versus 58.6% [601/1025] in those without severe adverse events [X2=15.1, P=0.001]) and 73.5% (25/34) required ICU admission (versus 51.4% [453/882] in those without [X2=6.45, P=0.01]).
*According to the original article.

© 2021 Nosadini M et al. *JAMA Neurology*.

eTable 6. Logistic regression model (single iteration) predicting poor functional outcome at 12 months—Statsmodel Logit() report (A) and derived odds ratios (B)

A

Logit Regression Results

Dep. Variable:	y	No. Observations:	582
Model:	Logit	Df Residuals:	548
Method:	MLE	Df Model:	33
Date:	Sat, 20 Mar 2021	Pseudo R-squ.:	0.1671
Time:	15:48:37	Log-Likelihood:	-304.36
converged:	True	LL-Null:	-365.41
Covariance Type:	nonrobust	LLR p-value:	3.742e-12

	coef	std err	z	P>|z|	[0.025	0.975]
const	-1.2231	0.643	-1.901	0.057	-2.484	0.038
Female	0.2931	0.242	1.210	0.226	-0.182	0.768
Infant	1.3138	0.810	1.622	0.105	-0.274	2.901
Child	-0.2267	0.296	-0.765	0.445	-0.808	0.354
Adolescent	-0.8683	0.289	-3.005	0.003	-1.435	-0.302
Older adult	1.0687	0.502	2.129	0.033	0.085	2.053
SpeechDys	-0.3038	0.221	-1.375	0.169	-0.737	0.129
Seizures	0.1566	0.244	0.642	0.521	-0.321	0.634
MvmtDis	0.3118	0.244	1.280	0.201	-0.166	0.789
Obtunded	0.2344	0.235	0.999	0.318	-0.225	0.694
BrainstemDys	0.4842	0.233	2.082	0.037	0.028	0.940
BehCogImp	-0.3757	0.297	-1.267	0.205	-0.957	0.206
ITU	0.5582	0.285	1.961	0.050	0.000	1.116
AbnMRIBrain	0.2757	0.210	1.316	0.188	-0.135	0.686
EEGSlowWithoutDB	-0.0818	0.248	-0.329	0.742	-0.569	0.405
EEGSlowWithDB	0.9443	0.378	2.496	0.013	0.203	1.686
EEGEpileptiform	0.1727	0.236	0.731	0.465	-0.290	0.636
CSFPleo	-0.0253	0.234	-0.108	0.914	-0.483	0.433
Tumour	-0.5964	0.254	-2.347	0.019	-1.095	-0.098
NoTreatmentWithin30Days	0.8089	0.228	3.552	0.000	0.363	1.255
IT1stLineComboCS	-0.4906	0.529	-0.927	0.354	-1.528	0.547
IT1stLineComboIVIG	-0.7771	0.585	-1.329	0.184	-1.923	0.369
IT1stLineComboTPE	-1.6607	0.949	-1.751	0.080	-3.520	0.199
IT1stLineComboCS_IVIG	-1.0094	0.460	-2.193	0.028	-1.911	-0.107
IT1stLineComboCS_TPE	-1.1854	0.545	-2.176	0.030	-2.253	-0.117
IT1stLineComboCS_IVIG_TPE	-0.9741	0.498	-1.955	0.051	-1.951	0.002
IT1stLineComboIVIG_TPE	-0.1970	0.664	-0.297	0.767	-1.498	1.104
IT2ndLineRTX	0.1366	0.256	0.533	0.594	-0.366	0.639
IT2ndLineCYC	0.3722	0.285	1.304	0.192	-0.187	0.932
ITMaintenanceMMF	-0.0443	0.676	-0.066	0.948	-1.368	1.280
ITMaintenanceAZA	0.1775	0.686	0.259	0.796	-1.166	1.521
IT6mSteroid	-1.5908	1.139	-1.396	0.163	-3.824	0.642
IT6mIVIG	2.1934	0.910	2.411	0.016	0.410	3.977
WorstMRSAcute 5	0.1799	0.279	0.645	0.519	-0.366	0.726

B

Feature	Odds ratio (95% CI)	p
Female	1.34 (0.83-2.16)	0.226
Infant (<2 years)	3.72 (0.76-18.19)	0.105
Child (2-11 years)	0.80 (0.45-1.43)	0.445
Adolescent (12-19 years)	0.42 (0.24-0.74)	0.003 *
Older adult (>=65 years)	2.91 (1.09-7.79)	0.033 *
Speech dysfunction	0.74 (0.48-1.14)	0.169
Seizures	1.17 (0.73-1.89)	0.521
Movement disorder	1.37 (0.85-2.20)	0.201
Decreased level of consciousness	1.26 (0.80-2.00)	0.318
Autonomic dysfunction/central hypoventilation	1.62 (1.03-2.56)	0.037 *
Abnormal behaviour and/or cognitive dysfunction	0.69 (0.38-1.23)	0.205

© 2021 Nosadini M et al. *JAMA Neurology.*

Intensive care unit admission	1.75 (1.00-3.05)	0.050 *
Abnormal MRI brain	1.32 (0.87-1.99)	0.188
EEG: background slowing without extreme delta brush	0.92 (0.57-1.50)	0.742
EEG: background slowing with extreme delta brush	2.57 (1.22-5.40)	0.013 *
EEG: epileptiform activity	1.19 (0.75-1.89)	0.465
CSF pleocytosis	0.97 (0.62-1.54)	0.914
Tumour	0.55 (0.33-0.91)	0.019 *
No immunotherapy within 30 days of disease onset	2.25 (1.44-3.51)	0.000 *
Corticosteroids only	0.61 (0.22-1.73)	0.354
IVIG only	0.46 (0.15-1.45)	0.184
TPE only	0.19 (0.03-1.22)	0.080
Corticosteroids and IVIG	0.36 (0.15-0.90)	0.028 *
Corticosteroids and TPE	0.31 (0.11-0.89)	0.030 *
Corticosteroids, IVIG and TPE	0.38 (0.14-1.00)	0.051
IVIG and TPE	0.82 (0.22-3.01)	0.767
Rituximab	1.15 (0.69-1.89)	0.594
Cyclophosphamide	1.45 (0.83-2.54)	0.192
Mycophenolate mofetil	0.96 (0.25-3.60)	0.948
Azathioprine	1.19 (0.31-4.58)	0.796
Corticosteroids for at least 6 months from first event	0.20 (0.02-1.90)	0.163
IVIG for at least 6 months from first event	8.97 (1.51-53.33)	0.016 *
Severe disability (mRS 5) in the acute phase	1.20 (0.69-2.07)	0.519

eTable 6. Logistic regression model (single iteration) predicting poor functional outcome at 12 months - Statsmodel *Logit()* report (A) and derived odds ratios (B).

© 2021 Nosadini M et al. *JAMA Neurology*.

eTable 7. Logistic regression model (single iteration) predicting relapsing disease course at ≥24 months—Statsmodel Logit() report (A) and derived odds ratios (B)

A

Logit Regression Results

Dep. Variable:	y	No. Observations:	410
Model:	Logit	Df Residuals:	376
Method:	MLE	Df Model:	33
Date:	Sat, 20 Mar 2021	Pseudo R-squ.:	0.1285
Time:	15:53:28	Log-Likelihood:	-245.41
converged:	True	LL-Null:	-281.60
Covariance Type:	nonrobust	LLR p-value:	9.017e-05

	coef	std err	z	P>|z|	[0.025	0.975]
const	0.1110	0.629	0.176	0.860	-1.123	1.345
Female	0.2195	0.278	0.789	0.430	-0.326	0.765
Infant	-0.0722	1.080	-0.067	0.947	-2.188	2.044
Child	0.2174	0.333	0.652	0.514	-0.436	0.871
Adolescent	0.6006	0.283	2.125	0.034	0.047	1.155
Older adult	-0.3426	0.923	-0.371	0.710	-2.151	1.466
SpeechDys	0.0793	0.261	0.304	0.761	-0.433	0.591
Seizures	0.0524	0.266	0.197	0.844	-0.468	0.573
MvmtDis	-0.3264	0.253	-1.288	0.198	-0.823	0.170
Obtunded	-0.1126	0.247	-0.456	0.649	-0.597	0.372
BrainstemDys	-0.2327	0.265	-0.880	0.379	-0.751	0.286
BehCogImp	-0.0633	0.332	-0.191	0.849	-0.714	0.588
ITU	-0.0054	0.329	-0.017	0.987	-0.649	0.639
AbnMRIBrain	-0.2703	0.236	-1.144	0.252	-0.733	0.193
EEGSlowWithoutDB	-0.3660	0.250	-1.465	0.143	-0.855	0.123
EEGSlowWithDB	0.3601	0.500	0.720	0.472	-0.620	1.340
EEGEpileptiform	-0.1548	0.266	-0.582	0.560	-0.676	0.366
CSFPleo	0.1859	0.243	0.765	0.444	-0.290	0.662
Tumour	-0.8392	0.324	-2.587	0.010	-1.475	-0.203
NoTreatmentWithin30Days	0.4318	0.273	1.582	0.114	-0.103	0.967
IT1stLineComboCS	0.1965	0.393	0.500	0.617	-0.574	0.967
IT1stLineComboIVIG	-0.1313	0.936	-0.140	0.888	-1.966	1.703
IT1stLineComboTPE	0.6685	1.483	0.451	0.652	-2.239	3.576
IT1stLineComboCS_IVIG	0.3195	0.341	0.936	0.349	-0.349	0.988
IT1stLineComboCS_TPE	0.1246	0.500	0.249	0.803	-0.855	1.105
IT1stLineComboCS_IVIG_TPE	-0.5978	0.454	-1.316	0.188	-1.488	0.293
IT1stLineComboIVIG_TPE	0.2951	0.749	0.394	0.694	-1.173	1.764
IT2ndLineRTX	-1.3081	0.412	-3.175	0.001	-2.116	-0.501
IT2ndLineCYC	-0.3335	0.434	-0.768	0.442	-1.184	0.517
ITMaintenanceMMF	-0.5899	0.925	-0.638	0.523	-2.402	1.222
ITMaintenanceAZA	-1.1538	0.800	-1.443	0.149	-2.721	0.413
IT6mSteroid	0.1159	0.827	0.140	0.889	-1.505	1.737
IT6mIVIG	-1.8327	1.163	-1.576	0.115	-4.112	0.447
WorstMRSAcute 5	-0.2393	0.320	-0.747	0.455	-0.867	0.389

B

Feature	Odds ratio (95% CI)	p
Female	1.25 (0.72-2.15)	0.430
Infant (<2 years)	0.93 (0.11-7.72)	0.947
Child (2-11 years)	1.24 (0.65-2.39)	0.514
Adolescent (12-19 years)	1.82 (1.05-3.17)	0.034 *
Older adult (>=65 years)	0.71 (0.12-4.33)	0.710
Speech dysfunction	1.08 (0.65-1.81)	0.761
Seizures	1.05 (0.63-1.77)	0.844
Movement disorder	0.72 (0.44-1.19)	0.198
Decreased level of consciousness	0.89 (0.55-1.45)	0.649
Autonomic dysfunction/central hypoventilation	0.79 (0.47-1.33)	0.379
Abnormal behaviour and/or cognitive dysfunction	0.94 (0.49-1.80)	0.849
Intensive care unit admission	0.99 (0.52-1.89)	0.987

© 2021 Nosadini M et al. *JAMA Neurology*.

Abnormal MRI brain	0.76 (0.48-1.21)	0.252
EEG: background slowing without extreme delta brush	0.69 (0.43-1.13)	0.143
EEG: background slowing with extreme delta brush	1.43 (0.54-3.82)	0.472
EEG: epileptiform activity	0.86 (0.51-1.44)	0.560
CSF pleocytosis	1.20 (0.75-1.94)	0.444
Tumour	0.43 (0.23-0.82)	0.010 *
No immunotherapy within 30 days of disease onset	1.54 (0.90-2.63)	0.114
Corticosteroids only	1.22 (0.56-2.63)	0.617
IVIG only	0.88 (0.14-5.49)	0.888
TPE only	1.95 (0.11-35.71)	0.652
Corticosteroids and IVIG	1.38 (0.71-2.69)	0.349
Corticosteroids and TPE	1.13 (0.43-3.02)	0.803
Corticosteroids, IVIG and TPE	0.55 (0.23-1.34)	0.188
IVIG and TPE	1.34 (0.31-5.83)	0.694
Rituximab	0.27 (0.12-0.61)	0.001 *
Cyclophosphamide	0.72 (0.31-1.68)	0.442
Mycophenolate mofetil	0.55 (0.09-3.40)	0.523
Azathioprine	0.32 (0.07-1.51)	0.149
Corticosteroids for at least 6 months from first event	1.12 (0.22-5.68)	0.889
IVIG for at least 6 months from first event	0.16 (0.02-1.56)	0.115
Severe disability (mRS 5) in the acute phase	0.79 (0.42-1.47)	0.455

eTable 7. Logistic regression model (single iteration) predicting relapsing disease course at ≥24 months - Statsmodel *Logit()* report (A) and derived odds ratios (B).

© 2021 Nosadini M et al. *JAMA Neurology*.

eTable 8. Prior published studies comparing effectiveness of immunotherapy combinations

Reference	Study type<br>Number of patients (age)<br>Additional data on Methods	Main findings<br>(Statistical significance)	Interpretation according to the article's authors
**A. Literature data from articles comparing efficacy of first-line immunotherapies or first-line immunotherapy combinations (corticosteroids, IVIG, therapeutic apheresis)**			
DeSena 2015[3]	Observational, retrospective.<br>14 patients (children and adults).<br>The mRS of 10 of 14 NMDARE patients that received CS and TPE were retrospectively evaluated; all the 14 patients were also subjectively assessed with the point of largest sustained improvement.	In the patients who received both CS and TPE (10/14 patients), 7/10 after TPE had improved mRS versus 3/10 patients after CS. The average mRS improvement after CS in this group was -0.1 as compared with 0.4 after TPE.<br>Based on subjective chart review analysis of all 14 patients, the largest sustained improvement occurred immediately following the third–fifth exchange in 9/14 patients, whereas only 2/14 patients appeared to have had significant benefit immediately following CS.<br>(Statistics not available)	This is compelling preliminary data that suggests that CS may not be as effective compared to CS followed by TPE.
Suppiej 2016[4]	Systematic literature review.<br>242 patients (children).<br>Systematic literature review on TPE in paediatric NMDARE (2007–2015).	TPE was given with CS and IVIG in 69.5%, with CS only in 18%, with IVIG only in 7%, or was the only first-line treatment in 5.5%. Higher rates of full/substantial recovery at follow-up were observed with immunotherapy given within 30 days from onset (69.4%) compared to later (59.2%), and when TPE was associated with CS (66.7%) rather than not (46.7%).<br>(Statistics not available)	Trend towards a better outcome when TPE was used early, and when given with CS.
Zhang 2017[5]	Systematic literature review.<br>432 patients (children and adults).<br>Aim: to provide an overview of the clinical characteristics, treatments, and outcomes of NMDARE.	There were no significant differences among CS, IVIG or TPE used alone (p= 0.9172) or among combinations of every two of them (p=0.3059). With regard to the use of CS and IVIG, there were no significant differences between the outcomes of early combined treatment and sequential treatment (p=0.7277), or between using CS first and IVIG first (p=0.5422).	No significant differences were found among different first-line immunotherapies.
Sakpichaisakul 2018[6]	Observational, retrospective + prospective.<br>19 patients (children).<br>Aim: to investigate outcomes from different treatments for NMDARE. Three treatments group: CS alone, IVIG alone, and IVIG and CS were reviewed.	IVIG was administered to 13 (68%) and 6 (32%) only received CS.<br>Those receiving IVIG treatment with or without CS had greater improvement in mRS at 6 (p=0.04) and 12 months (p=0.03).	Such findings suggest the benefits of IVIG over CS despite the higher immediate cost.
Zhang 2019[7]	Prospective.<br>40 patients (children and adults).	Compared with the non-TPE group, the TPE group exhibited greater clinical improvement after 1 month and 2 months following treatment ($P < 0.05$). After 3 months, 6 months, and 12	TPE might rapidly improve the clinical manifestations in patients with severe refractory NMDARE.

© 2021 Nosadini M et al. *JAMA Neurology*.

	Patients with severe NMDARE with no improvement after CS and/or IVIG for at least 10 days were enrolled. All received immunotherapy and were divided into a TPE group (19 patients, total 118 procedures) and a non-TPE group (21 patients).	months, there were no significant differences in the outcomes between the TPE group and non-TPE group.	
Zhang 2021[8]	Prospective.<br>57 patients with severe refractory autoimmune encephalitis, including NMDARE (n = 51).	Of all 57 patients, 33 patients received TPE. Compared with the non-TPE group, the TPE group exhibited greater clinical improvement: 21 (37%) versus 8 (14%) after 1 month (P = 0.03) and 31 (54%) versus 16 (28%) after 2 months (P = 0.01), respectively. Complications and adverse events associated with TPE occurred in 91 procedures (47%) without serious adverse events associated with the use of TPE.	TPE might be an effective rescue therapy associated with rapid functional improvement in patients with severe steroid/IVIG refractory antibody-associated autoimmune encephalitis.
**B. Literature data from articles comparing the efficacy of adjunctive second-line immunotherapy (rituximab and/or cyclophosphamide) with first-line only**			
Titulaer 2013[1]	Observational, retrospective.<br>577 patients (children and adults).<br>Multi-institutional observational study (2007-2012).	Of 221 patients who failed first-line therapy, 125 (57%) received second-line immunotherapy resulting in better outcome than those who did not (OR 2·69, CI 1·24-5·80, p=0·012).	Second-line immunotherapy is usually effective when first-line therapies fail.
Nosadini 2019[9]	Observational, retrospective.<br>62 patients (children).<br>Retrospective study of an Italian cohort of patients with paediatric (≤18y) onset NMDARE.	At the survival analysis, the risk of relapsing was significantly lower in patients who received three or more different immunotherapies at first disease event (hazard ratio 0.208, 95% confidence interval 0.046-0.941; p=0.042). In this subgroup, 58.8% of patients received TPE (20/34), and 88.2% received second-line immunotherapy (30/34).	Aggressive immunotherapy at onset appears to decrease risk of relapse.
Lee 2020[10]	Prospective.<br>78 patients (children and adults).<br>Consecutive patients treated for NMDARE between Jan 2014 and Oct 2019 in a national referral hospital.<br>T-SIRT= tumor removal, CS, IVIG, RTX, TCZ	In a linear mixed model analysis, using the SIRT regimen was more effective than SIR or SI regimens in lowering CASE scores (P < 0.001 and P = 0.001, respectively). Completion of the (T)-SIRT regimen within 1 month of onset resulted in better 1-year improvements in CASE score (P < 0.001) and mRS scores (P = 0.001), compared to those of using other regimens within 1 month or delaying teratoma removal for more than 1 month.	Early application of combined immunotherapy consisting of T-SIRT had better efficacy than delayed or partial application of this combination.

eTable 8. Prior published studies comparing effectiveness of immunotherapy combinations. A. Literature data from articles comparing efficacy of first-line immunotherapies or first-line immunotherapy combinations (corticosteroids, IVIG, therapeutic apheresis) in NMDAR-antibody encephalitis. B. Literature data from articles comparing the efficacy of adjunctive second-line immunotherapy (rituximab and/or cyclophosphamide) with first-line only in NMDAR-antibody encephalitis.
Legend: CS: corticosteroids; IVIG: intravenous immunoglobulin; mRS: modified Rankin Scale; NMDARE: anti-N-methyl-D-aspartate receptor encephalitis; RTX: rituximab; TCZ: tocilizumab; TPE: therapeutic plasma exchange.

© 2021 Nosadini M et al. *JAMA Neurology*.

eTable 9. Age-stratified associations of rituximab with relapsing disease course

Age group	Total	Proportion with relapsing disease course		Odds ratio (95% CI)	*P*
		Treated with rituximab	Not treated with rituximab		
**Infant (≤2 years)**	4	0/0	2/4 (50.0%)	1.00 (0.01-80.05)	1.00
**Child (2-11 years)**	94	4/19 (21.1%)	37/73 (50.7%)	0.26 (0.08-0.86)	0.03
**Adolescent (12-19 years)**	117	2/13 (15.4%)	49/91 (53.8%)	0.16 (0.03-0.74)	0.02
**Adult (20-64 years)**	179	2/18 (11.1%)	64/147 (43.5%)	0.16 (0.04-0.73)	0.02
**Older adult (≥65 years)**	8	0/1 (0.0%)	2/7 (28.6%)	1.25 (0.03-54.23)	0.91

eTable 9. Age-stratified relationship of rituximab with relapsing disease course. Cases with known long-term relapse outcome (clinical relapse at any time, or absence of relapse after ≥24 months follow-up) were selected from the unimputed dataset and univariate comparisons performed for each age category separately. Odds ratios and *P* values calculated with statsmodels v0.12.2. Rituximab was associated with significantly reduced risk for relapsing disease across all three main age categories (child, adolescent and adult).

© 2021 Nosadini M et al. *JAMA Neurology*.

eTable 10. Comparison of patients receiving earlier vs later second-line immunotherapy

	**Second-line IT initiation <60 days after disease onset**	**Second-line IT initiation ≥60 days after disease onset**	***P***
**Number of patients with data available**	88	71	-
**Worst mRS in the acute phase**	Median 5, mean 4.6, range 3-5 (d.a.: 87/88)	Median 5, mean 4.6, range 3-5 (d.a.: 63/71)	0.80
**Admission to the intensive care unit**	54/88 (61.4%)	35/61 (57.4%)	0.63
**Length of follow-up (months)**	Median 8.7, mean 10.5, range 1.4-48 (d.a.: 74/88)	Median 16, mean 22.4, range 3-93 (d.a.: 59/71)	<0.001
**mRS at last follow-up***	Median 2, mean 2.0, range 0-6 (d.a.: 80/88)	Median 2, mean 2.3, range 0-6 (d.a.: 67/71)	0.30
**Poor functional outcome at 12 months****	10/46 (21.7%)	22/33 (66.7%)	<0.001

eTable 10. Comparison of patients receiving earlier (<60 days from onset) vs. later (≥60 days after onset) second-line immunotherapy. The Chi-square test was used for nominal data and the Mann-Whitney U test for continuous or ordinal data. Those receiving earlier second-line immunotherapy had significantly reduced odds for poor functional outcome at 12 months compared to those receiving later second-line immunotherapy (OR 0.14, 95% CI 0.05-0.38).
d.a., data available.
*Including all patients with available data (at any follow-up duration).
**Including only cases with available data for ascertainment of good vs. poor functional outcome at 12 months, as defined in the main methods.

© 2021 Nosadini M et al. *JAMA Neurology*.

eTable 11. Main factors associated with outcome and disease course in the large cohort by Titulaer et al[1] and our systematic review

	**Association with good functional outcome**		**Association with fewer relapses**	
**Variable**	**Titulaer *et al.* 2013[a]**	**Our analysis[b]**	**Titulaer *et al.* 2013[c]**	**Our analysis[b]**
Early treatment	+	+	na	ns
Use of first-line immunotherapy	na	+	+[d]	ns
Use of second-line immunotherapy	+	ns	+[d]	+
Use of maintenance IVIG	na	-	na	+
Presence of tumour	na	ns	+	ns
ICU admission	-	-	na	ns
Infant or older adult age	na	-	na	ns
Adolescent age	na	+	na	-
EEG extreme delta brush pattern	na	-	na	ns

eTable 11. Main factors associated with outcome and relapsing disease course in the large cohort by Titulaer *et al.*[1] and our systematic review. Only variables with statistical significance in one or both analyses are displayed.

+	Positive association
-	Negative association
na	Not assessed
ns	Not significant at $p<0.05$

a, Multivariable analysis
b, Bootstrapped multivariable analysis
c, Univariate analysis
d, Patients without tumour only

© 2021 Nosadini M et al. *JAMA Neurology*.

eTable 12. Logistic regression models for poor functional outcome at 12 months with interaction terms

Model	Variables included	Coef	SE	z	Odds ratio (95% CI)	*P*
1 (Base)	Female	0.0359	0.213	0.169	1.04 (0.68-1.57)	0.87
	Infant (<2 years)	1.5515	0.739	2.100	4.72 (1.11-20.08)	0.04
	Child (2-11 years)	-0.3446	0.243	-1.420	0.71 (0.44-1.14)	0.16
	Adolescent (12-19 years)	-1.0439	0.257	-4.061	0.35 (0.21-0.58)	<0.001
	Older adult (>=65 years)	1.0991	0.457	2.408	3.00 (1.23-7.34)	0.02
	ICU admission	0.5914	0.257	2.301	1.81 (1.09-2.99)	0.02
	Severe disease (mRS 5) in the acute phase	0.2925	0.255	1.148	1.34 (0.81-2.21)	0.25
	First-line IT without second-line	-1.2624	0.423	-2.987	0.28 (0.12-0.65)	0.003
	First-line and second-line IT	-0.8762	0.439	-1.998	0.42 (0.18-0.98)	0.05
2	Female	0.0471	0.214	0.220	1.05 (0.69-1.59)	0.83
	Infant (<2 years)	1.5440	0.737	2.095	4.68 (1.10-19.85)	0.04
	Child (2-11 years)	-0.3886	0.245	-1.589	0.68 (0.42-1.09)	0.11
	Adolescent (12-19 years)	-1.0932	0.262	-4.167	0.34 (0.20-0.56)	<0.001
	Older adult (>=65 years)	1.0372	0.457	2.270	2.82 (1.15-6.91)	0.02
	ICU admission	0.9332	0.926	1.007	2.54 (0.41-15.63)	0.31
	Severe disease (mRS 5) in the acute phase	0.3262	0.257	1.269	1.39 (0.84-2.29)	0.21
	First-line IT without second-line	-1.0147	0.498	-2.039	0.36 (0.14-0.96)	0.04
	First-line and second-line IT	-1.1597	0.556	-2.087	0.31 (0.11-0.93)	0.04
	ICU admission * First-line IT without second-line	-0.6623	0.951	-0.696	0.52 (0.08-3.33)	0.49
	ICU admission * First-line and second-line IT	0.1766	0.981	0.180	1.19 (0.17-8.16)	0.86
3	Female	0.0391	0.214	0.183	1.04 (0.68-1.58)	0.86
	Infant (<2 years)	1.5731	0.738	2.133	4.82 (1.14-20.47)	0.03
	Child (2-11 years)	-0.3542	0.243	-1.457	0.70 (0.44-1.13)	0.15
	Adolescent (12-19 years)	-1.0645	0.260	-4.097	0.34 (0.21-0.57)	<0.001
	Older adult (>=65 years)	1.0880	0.458	2.377	2.97 (1.21-7.28)	0.02
	ICU admission	0.6074	0.259	2.348	1.84 (1.11-3.05)	0.02
	Severe disease (mRS 5) in the acute phase	0.4908	0.853	0.575	1.63 (0.31-8.69)	0.57

© 2021 Nosadini M et al. *JAMA Neurology*.

	First-line IT without second-line	-1.1309	0.530	-2.133	0.32 (0.11-0.91)	0.03
	First-line and second-line IT	-0.9268	0.575	-1.612	0.40 (0.13-1.22)	0.11
	mRS 5 in the acute phase * First-line IT without second-line	-0.3158	0.884	-0.357	0.73 (0.13-4.12)	0.72
	mRS 5 in the acute phase * First-line and second-line IT	-0.0373	0.912	-0.041	0.96 (0.16-5.76)	0.97
4	Female	-0.7896	0.891	-0.886	0.45 (0.08-2.60)	0.38
	Infant (<2 years)	1.5855	0.737	2.150	4.88 (1.15-20.72)	0.03
	Child (2-11 years)	-0.3209	0.244	-1.318	0.73 (0.45-1.17)	0.19
	Adolescent (12-19 years)	-1.0631	0.259	-4.109	0.35 (0.21-0.57)	<0.001
	Older adult (>=65 years)	1.1500	0.461	2.496	3.16 (1.28-7.79)	0.01
	ICU admission	0.6166	0.258	2.388	1.85 (1.12-3.07)	0.02
	Severe disease (mRS 5) in the acute phase	0.2733	0.256	1.068	1.31 (0.80-2.17)	0.29
	First-line IT without second-line	-1.7374	0.780	-2.228	0.18 (0.04-0.81)	0.03
	First-line and second-line IT	-1.7784	0.819	-2.171	0.17 (0.03-0.84)	0.03
	Female * First-line IT without second-line	0.6772	0.931	0.728	1.97 (0.32-12.20)	0.47
	Female * First-line and second-line IT	1.2614	0.966	1.306	3.53 (0.53-23.44)	0.19
5**	Female	0.0322	0.215	0.150	1.03 (0.68-1.57)	0.88
	Infant (<2 years)	7.4272	6227	0.003	3.7x10^7 (0.00-inf)	1.00
	Child (2-11 years)	-0.3549	0.243	-1.461	0.70 (0.44-1.13)	0.14
	Adolescent (12-19 years)	-1.0460	0.257	-4.068	0.35 (0.21-0.58)	<0.001
	Older adult (>=65 years)	1.0995	0.457	2.406	3.00 (1.23-7.35)	0.02
	ICU admission	0.5969	0.258	2.315	1.82 (1.10-3.01)	0.02
	Severe disease (mRS 5) in the acute phase	0.2615	0.256	1.021	1.30 (0.79-2.15)	0.31
	First-line IT without second-line	-1.2470	0.427	-2.919	0.29 (0.12-0.66)	0.004
	First-line and second-line IT	-0.8063	0.443	-1.821	0.45 (0.19-1.06)	0.07
	Infant * First-line IT without second-line	-4.8001	6227	-0.002	0.00 (0.00-inf)	1.00
	Infant * First-line and second-line IT	-4.6203	8403	-0.004	0.00 (0.00-inf)	1.00
6	Female	0.0319	0.214	0.149	1.03 (0.68-1.57)	0.89
	Infant (<2 years)	1.5610	0.739	2.111	4.76 (1.12-20.29)	0.04
	Child (2-11 years)	-0.1808	1.110	-0.163	0.83 (0.09-7.35)	0.87
	Adolescent (12-19 years)	-1.0471	0.258	-4.061	0.35 (0.21-0.58)	<0.001

© 2021 Nosadini M et al. *JAMA Neurology*.

	Older adult (>=65 years)	1.0985	0.458	2.401	3.00 (1.22-7.35)	0.02
	ICU admission	0.5965	0.258	2.310	1.82 (1.09-3.01)	0.02
	Severe disease (mRS 5) in the acute phase	0.2980	0.256	1.166	1.35 (0.82-2.22)	0.24
	First-line IT without second-line	-1.2602	0.462	-2.726	0.28 (0.11-0.70)	0.006
	First-line and second-line IT	-0.8158	0.479	-1.702	0.44 (0.17-1.13)	0.09
	Child * First-line IT without second-line	-0.0736	1.145	-0.064	0.93 (0.10-8.76)	0.95
	Child * First-line and second-line IT	-0.3168	1.165	-0.272	0.73 (0.07-7.15)	0.79
7	Female	0.0444	0.214	0.208	1.05 (0.69-1.59)	0.84
	Infant (<2 years)	1.5601	0.739	2.111	4.76 (1.12-20.25)	0.04
	Child (2-11 years)	-0.3482	0.243	-1.433	0.71 (0.44-1.14)	0.15
	Adolescent (12-19 years)	-0.8433	0.828	-1.019	0.43 (0.08-2.18)	0.31
	Older adult (>=65 years)	1.1011	0.457	2.409	3.01 (1.23-7.37)	0.02
	ICU admission	0.5843	0.257	2.275	1.79 (1.08-2.97)	0.02
	Severe disease (mRS 5) in the acute phase	0.3009	0.255	1.181	1.35 (0.82-2.23)	0.24
	First-line IT without second-line	-1.2090	0.517	-2.339	0.30 (0.11-0.82)	0.02
	First-line and second-line IT	-0.7630	0.534	-1.429	0.47 (0.16-1.33)	0.15
	Adolescent * First-line IT without second-line	-0.0909	0.885	-0.103	0.91 (0.16-5.17)	0.92
	Adolescent * First-line and second-line IT	-0.4222	0.923	-0.457	0.66 (0.11-4.00)	0.65
8**	Female	-0.0045	0.215	-0.021	1.00 (0.65-1.52)	0.98
	Infant (<2 years)	1.5735	0.739	2.129	4.82 (1.13-20.53)	0.03
	Child (2-11 years)	-0.3535	0.244	-1.451	0.70 (0.44-1.13)	0.15
	Adolescent (12-19 years)	-1.0524	0.258	-4.084	0.35 (0.21-0.58)	<0.001
	Older adult (>=65 years)	9.9740	17300	0.001	4.7x10^8 (0.00-inf)	1.00
	ICU admission	0.6587	0.260	2.533	1.93 (1.16-3.22)	0.01
	Severe disease (mRS 5) in the acute phase	0.2494	0.257	0.970	1.28 (0.78-2.12)	0.33
	First-line IT without second-line	-1.2386	0.435	-2.846	0.29 (0.12-0.68)	0.004
	First-line and second-line IT	-0.7567	0.450	-1.682	0.47 (0.19-1.13)	0.09
	Older adult * First-line IT without second-line	-8.1675	17300	-0.001	0.00 (0.00-inf)	1.00
	Older adult * First-line and second-line IT	-0.1446	17300	-0.001	0.00 (0.00-inf)	1.00

eTable 12. Logistic regression models for poor functional outcome at 12 months with interaction terms.

© 2021 Nosadini M et al. *JAMA Neurology*.

x1 * x2 indicates the interaction term between x1 and x2.
**Model failed to converge. Coef: beta coefficient; ICU: intensive care unit; IT: immunotherapy; mRS: modified Rankin Scale; SE: standard error.
In each model the dependent variable was poor functional outcome at 12 months (defined as in the main multivariable modelling), first-line immunotherapy was defined as corticosteroids, therapeutic apheresis or IVIG (alone or in any combination), and second-line immunotherapy was defined as rituximab, cyclophosphamide, bortezomib or tocilizumab (alone or in any combination). Patients receiving second-line immunotherapy without first-line immunotherapy (n=2) were excluded, leaving 580 patients in the dataset, entered in full to each model (i.e. without bootstrapping).
Model 1 is the base model (without interaction terms). This can be regarded as a feature-reduced version of the multivariable model presented in the main manuscript. As in the main model, infant age, older adult age and ICU admission were significantly associated with increased odds of poor outcome, while adolescent age was significantly associated with good outcome (i.e. decreased OR for poor outcome). Immunotherapy (first-line immunotherapy alone or first-line and second-line immunotherapy together) was also associated with good outcome.
In models 2-8, additional interaction terms (indicated by x1 * x2 in the table) were added to the base model, to evaluate specific interaction effects between severity and demographic factors and immunotherapy. In none of these models were the interaction terms significant at $p<0.05$. We conclude that there were no significant interactions between immunotherapy and severity or demographic factors on the dependent variable (functional outcome at 12 months) in this dataset, while acknowledging that our power to detect such interactions may be limited by the sample size.

© 2021 Nosadini M et al. *JAMA Neurology*.

eFigure 1. PRISMA flow diagram

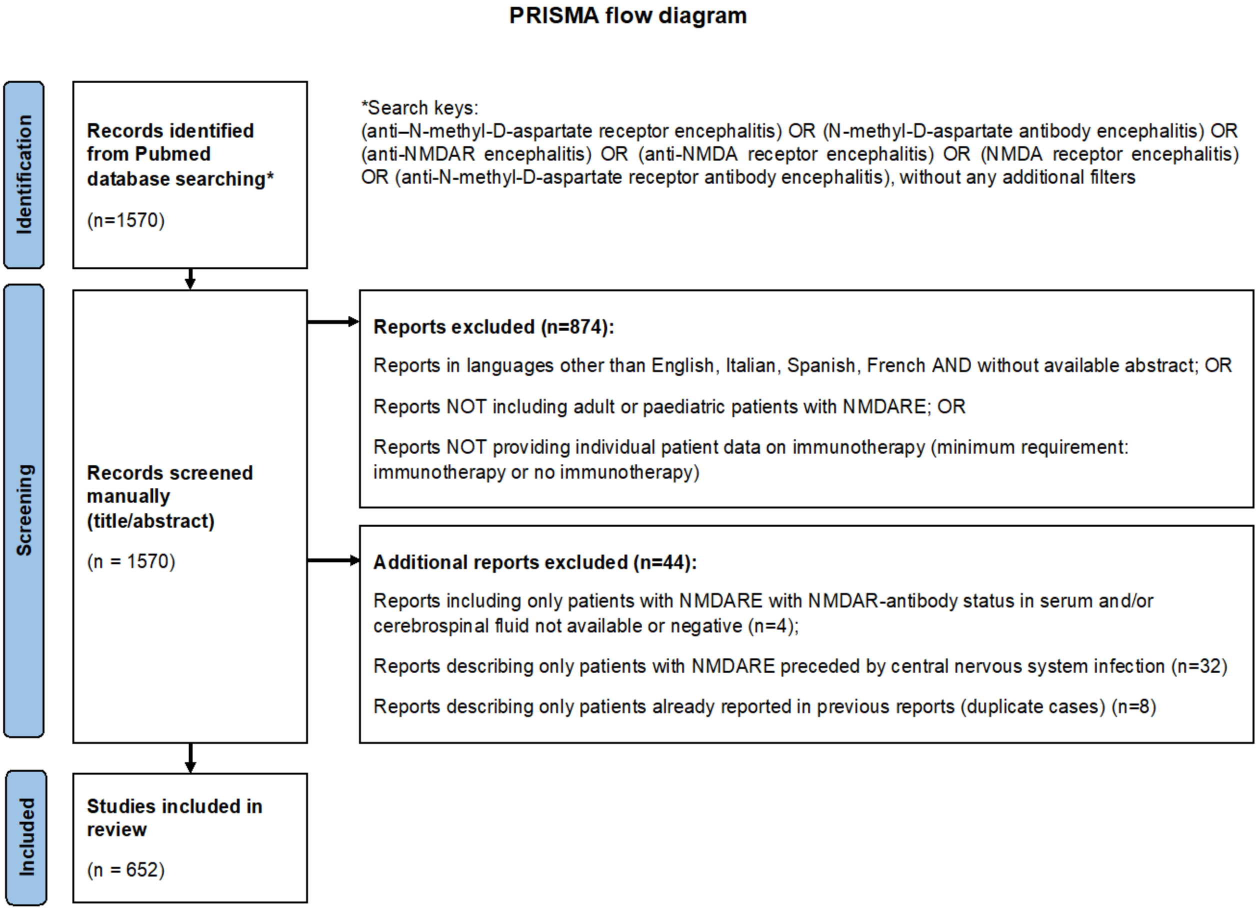

Legend: PRISMA flow diagram.

© 2021 Nosadini M et al. *JAMA Neurology*.

eFigure 2. Number of patients extracted per published report

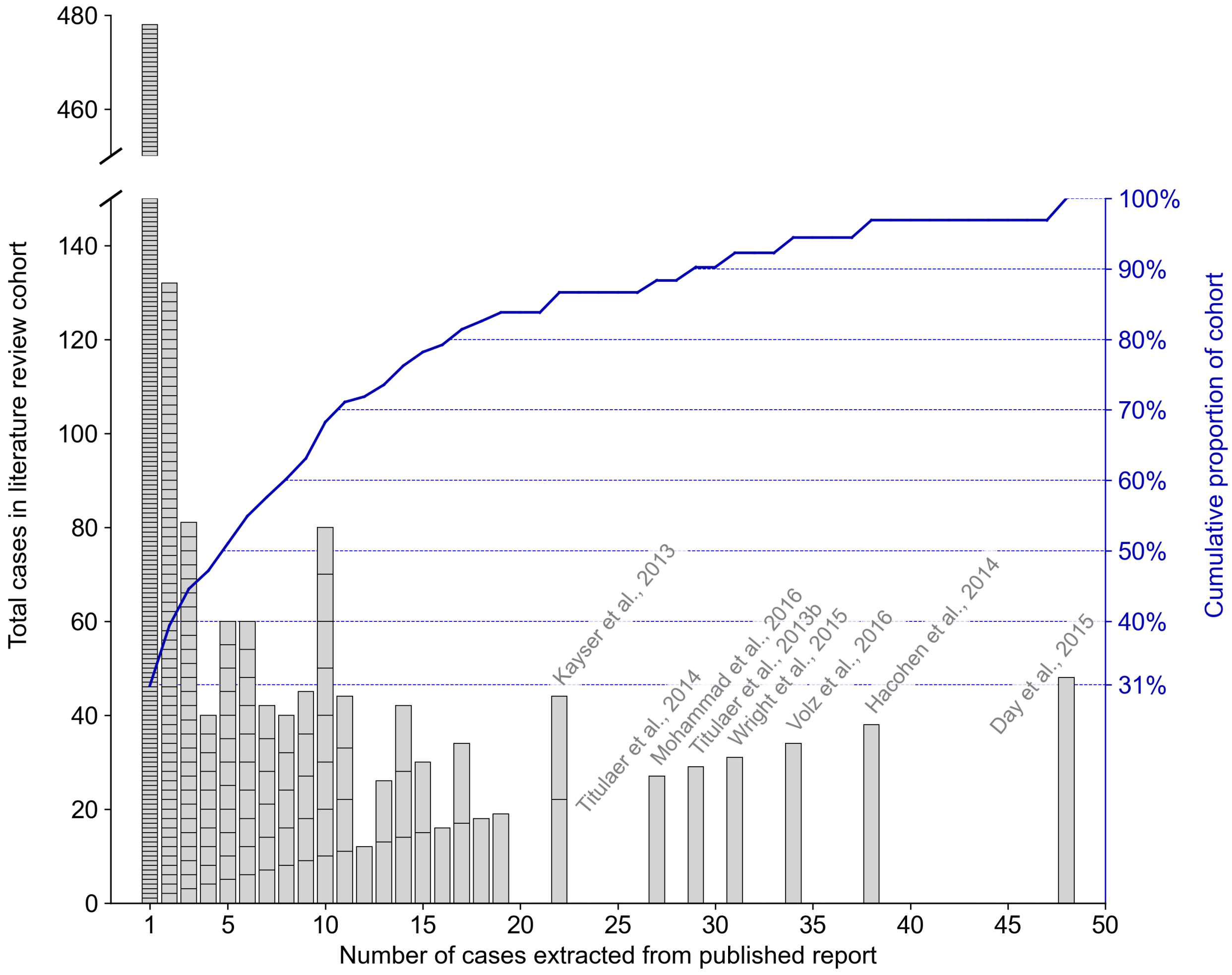

Legend: 1550 cases were extracted from 652 primary reports. For cases described over multiple papers, the paper contributing the most cases overall to the cohort was considered the primary report. Each bounded grey area represents one primary report, with size proportional to the number of cases extracted.
Reports providing ≥20 cases are cited:
Kayser et al., 2013[11]
Titulaer et al., 2014[12]
Mohammad et al., 2016[13]
Titulaer et al., 2013b[14]
Wright et al., 2015[15]
Volz et al., 2016[16]
Hacohen et al., 2014[17]
Day et al., 2015[18]

© 2021 Nosadini M et al. *JAMA Neurology*.

eMethods 1. Additional data on temporal epochs

Changes in immunotherapy use and disease outcome with time were primarily analysed over two epochs: before and after Titulaer *et al.*[1] Where year of disease onset was reported, cases with onset in 2013 or later were assigned to the later epoch and others to the early epoch; otherwise, cases extracted from papers published after 2013 (or in 2013 if citing Titulaer et al.[1]) were assigned to the later epoch and others to the early epoch. Additional post hoc analyses over six epochs (with cutoffs at two-year intervals) were performed for relapse rate and functional outcome, tested for significance with the Cuzick-Wilcoxon nonparametric test for trend.

© 2021 Nosadini M et al. *JAMA Neurology*.

eMethods 2. Statistical appendix

Alternative imputation methods
As data were not missing completely at random (Little's MCAR test: X2=359.005, DF=228, P<0.001),[19] multivariable imputation approaches were explored. Three imputation methods were evaluated[20] on an earlier version of the model targeting functional outcome at 12 months: (i) hot deck imputation, (ii) k-Nearest Neighbours (KNN) imputation implemented in Python 3.6 with MissingPy,[21] and (iii) multiple regression imputation implemented with SPSS.[22, 23]

In KNN imputation each sample's missing values are imputed as the average of the *n* nearest neighbors found in the training set. This can lead to imputed values which would not be possible in the observed data (e.g., mRS = 2.6). SPSS uses a fully conditional specification, or imputation through chain of equations, which employs the filled-in variable from one step as a predictor in all subsequent steps of imputation. Linear regression is applied to the continuous variables, and logistic regression is used for the categorical variables. It is known that, for high proportions of data missing not at random, this method introduces relevant biases.

Hot deck imputation was favoured for this study due to its more conservative approach and superior prediction accuracy:
Accuracy for KNN imputation = 80.5%
Accuracy for hot deck imputation = 81.1%
Accuracy for multiple regression imputation = 80.0%

Hot deck procedure and stability
Hot deck methods impute missing values within a database, by using available values (donors) from the same database. In this study, hot deck method was deployed, with no limit on the number of single donor selections. Case distance was weighted by univariate association of each variable with the target (functional outcome at 12 months). Missing values were filled sequentially, starting with the variable with minimum frequency of missingness (reference imputation). To test the stability of the donors selection, imputations were repeated by introducing single-variable weight changes equal to ±1%, ±5% and ±10% of the original univariate weight. In all tests (weight change x variable) the complete imputed database differed from the reference imputation in less than 2% of imputed values. Reference imputation was employed in all the analyses.

Bootstrapped multivariable regression
Regression models were implemented with the Statsmodels *Logit()* function in Python 3.6. Model parameters were not carried forward between iterations. Odds ratios, confidence intervals and P-values were derived from the distributions of the regression coefficients over the 10,000 folds.

Class-balanced pseudorandomisation procedure
In order to stabilise the Statsmodel *Logit()* function against algebraic errors caused by 100% imbalance in the target variable with respect to infrequently occurring predictor variables, an additional balancing procedure was applied within the bootstrapped regression. The procedure was applied homogeneously across all variables to avoid bias. Prior to the train-test shuffle, one case meeting the criteria [Predictor=1, Target=1] and one case meeting the criteria [Predictor =1, Target=0] were randomly selected from the dataset for each of the 33 predictors (without replacement). These 66 cases were held out of the train-test shuffle, then appended to the training set within each fold of the bootstrap. The train-test ratio was adjusted to compensate for the procedure, such that the final number of cases in the training and testing sets was equal.

© 2021 Nosadini M et al. *JAMA Neurology*.

eResults. Descriptive data on outcome and relapses in relation to immunotherapy

Data on relapses in patients treated with rituximab and with maintenance immunotherapy

Among 363 patients who received rituximab at first event, relapses occurred in 2.5% (8/319; data on relapses available in 319/363). In these relapsing patients with available data, rituximab was administered within 60 days from onset in 4/6 (data available in 6/8), and time between onset and relapse was median 3.5 months (mean 23.3, range 2.3-84; data on time between onset and relapse was available in 4/8); in the 4 relapsing patients with available time to relapse, rituximab had been administered within 60 days from onset in 2/3 (data available in 3/4).

Among the 146 patients who received maintenance immunotherapy for 6 months or longer at first event, relapses occurred in 6.3% (8/128; data on relapses available in 128/146). In these relapsing patients with available data, relapses occurred at median 35.5 months from onset (mean 33.1, range 1.5-72; data available in 6/8).

Data on outcome and relapses in relation to timing of second-line immunotherapy

Among 486 patients who received second-line immunotherapy at first disease event, in 55.3% (88/159) this was administered <60 days from disease onset (data on timing of administration available in 159/486).
In this subgroup who received second-line immunotherapy <60 days from disease onset, worst mRS in the acute phase was: mRS 3 in 11.5% (10/87), mRS 4 in 19.5% (17/87), mRS 5 in 69% (60/87) (data on mRS in the acute phase available in 87/88). 61.4% (54/88) required admission to the intensive care unit.
Relapses occurred in 4.8% (4/84; data on relapses available in 84/88).
At last follow-up (median 8.7 months, mean 10.5, range 1.4-48; data available in 74/88), median mRS was 2 (mean 2, range 0-6; data available in 80/88); 21.3% (17/80) patients had mRS 0, 21.3% (17/80) patients had mRS 1, 25% (20/80) had mRS 2, 16.3% (13/80) had mRS 3, 5% (4/80) had mRS 4, 7.5% (6/80) had mRS 5, 3.8% (3/80) died.

Among 486 patients who received second-line immunotherapy at first disease event, in 44.7% (71/159) this was administered ≥60 days from disease onset (data on timing of administration available in 159/486).
In this subgroup who received second-line immunotherapy ≥60 days from disease onset, worst mRS in the acute phase was: mRS 3 in 6.3% (4/63), mRS 4 in 23.8% (15/63), mRS 5 in 69.8% (44/63) (data on mRS in the acute phase available in 63/71). 57.4% (35/61; data available in 61/63) required admission to the intensive care unit.
Relapses occurred in 5.7% (4/70; data on relapses available in 70/71).
At last follow-up (median 16 months, mean 22.4, range 3-93; data available in 59/81), median mRS was 2 (mean 2.3, range 0-6; data available in 67/71); 16.4% (11/67) patients had mRS 0, 23.9% (16/67) patients had mRS 1, 11.9% (8/67) had mRS 2, 31.3% (21/67) had mRS 3, 6% (4/67) had mRS 4, 1.5% (1/67) had mRS 5, 9% (6/67) died.

© 2021 Nosadini M et al. *JAMA Neurology*.

eReferences.

1. Titulaer MJ, McCracken L, Gabilondo I, et al. Treatment and prognostic factors for long-term outcome in patients with anti-NMDA receptor encephalitis: an observational cohort study. *Lancet Neurol*. 2013;12(2):157-165.
2. Graus F, Titulaer MJ, Balu R, et al. A clinical approach to diagnosis of autoimmune encephalitis. *Lancet Neurol*. 2016;15(4):391-404.
3. DeSena AD, Noland DK, Matevosyan K, et al. Intravenous methylprednisolone versus therapeutic plasma exchange for treatment of anti-N-methyl-D-aspartate receptor antibody encephalitis: A retrospective review. *J Clin Apher*. 2015;30(4):212-216.
4. Suppiej A, Nosadini M, Zuliani L, et al. Plasma exchange in pediatric anti-NMDAR encephalitis: A systematic review. *Brain Dev*. 2016;38(7):613-622.
5. Zhang L, Wu MQ, Hao ZL, et al. Clinical characteristics, treatments, and outcomes of patients with anti-N-methyl-d-aspartate receptor encephalitis: A systematic review of reported cases. *Epilepsy Behav*. 2017;68:57-65.
6. Sakpichaisakul K, Patibat L, Wechapinan T, Sri-Udomkajrorn S, Apiwattanakul M, Suwannachote S. Heterogenous treatment for anti-NMDAR encephalitis in children leads to different outcomes 6-12 months after diagnosis. *J Neuroimmunol*. 2018;324:119-125.
7. Zhang Y, Liu G, Jiang M, Chen W, Su Y. Efficacy of Therapeutic Plasma Exchange in Patients with Severe Refractory Anti-NMDA Receptor Encephalitis. *Neurotherapeutics*. 2019;16(3):828-837.
8. Zhang Y, Huang HJ, Chen WB, Liu G, Liu F, Su YY. Clinical efficacy of plasma exchange in patients with autoimmune encephalitis. *Ann Clin Transl Neurol*. 20218(4):763-773.
9. Nosadini M, Granata T, Matricardi S, et al. Relapse risk factors in anti-N-methyl-D-aspartate receptor encephalitis. *Dev Med Child Neurol*. 2019;61(9):1101-1107.
10. Lee WJ, Lee ST, Shin YW, et al. Teratoma Removal, Steroid, IVIG, Rituximab and Tocilizumab (T-SIRT) in Anti-NMDAR Encephalitis. *Neurotherapeutics*. 2021;18(1):474-487.
11. Kayser MS, Titulaer MJ, Gresa-Arribas N, Dalmau J. Frequency and characteristics of isolated psychiatric episodes in anti–N-methyl-d-aspartate receptor encephalitis. *JAMA Neurol*. 2013;70(9):1133-1139.
12. Titulaer MJ, Höftberger R, Iizuka T, et al. Overlapping demyelinating syndromes and anti–N-methyl-D-aspartate receptor encephalitis. *Ann Neurol*. 2014;75(3):411-428.
13. Mohammad SS, Jones H, Hong M, et al. Symptomatic treatment of children with anti-NMDAR encephalitis. *Dev Med Child Neurol*. 2016;58(4):376-384.
14. Titulaer MJ, McCracken L, Gabilondo I, et al. Late-onset anti-NMDA receptor encephalitis. *Neurology*. 2013;81(12):1058-1063.
15. Wright S, Hacohen Y, Jacobson L, et al. N-methyl-D-aspartate receptor antibody-mediated neurological disease: results of a UK-based surveillance study in children. *Arch Dis Child*. 2015;100(6):521-526.
16. Volz MS, Finke C, Harms L, et al. Altered paired associative stimulation-induced plasticity in NMDAR encephalitis. *Ann Clin Transl Neurol*. 2016;3(2):101-113.
17. Hacohen Y, Absoud M, Hemingway C, et al. NMDA receptor antibodies associated with distinct white matter syndromes. *Neurol Neuroimmunol Neuroinflamm*. 2014;1(1):e2.
18. Day GS, Prüss H, Benseler SM, Paton TA, Paterson AD, Andrade DM. GRIN1 polymorphisms do not affect susceptibility or phenotype in NMDA receptor encephalitis. *Neurol Neuroimmunol Neuroinflamm*. 2015;2(5):e153.
19. Little RJ. A test of missing completely at random for multivariate data with missing values. *Journal of the American statistical Association* 1988;83:1198-1202.
20. Sterne JAC, White IR, Carlin JB, et al. Multiple imputation for missing data in epidemiological and clinical research: Potential and pitfalls. *BMJ*. 2009;338:b2393.
21. Stekhoven DJ, Bühlmann P. MissForest--non-parametric missing value imputation for mixed-type data. *Bioinformatics*. 2012;28(1):112-118.
22. Graham JW. Multiple Imputation and Analysis with SPSS 17-20. In: Missing Data. Statistics for Social and Behavioral Sciences. *Springer, New York, NY*. 2012. https://doi.org/10.1007/978-1-4614-4018-5_5
23. Horton N, Lipsitz S. Multiple Imputation in Practice: Comparison of Software Packages for Regression Models with Missing Variables. *The American Statistician*. 2001;55(3), 244-254.

© 2021 Nosadini M et al. *JAMA Neurology*.

eAppendix. Reports included in the final data set

First author	Reference
Abbas - Jacob, 2016	Extreme delta brushes and BIRDs in the EEG of anti-NMDA-receptor encephalitis.<br>Abbas A, Garg A, Jain R, Mazibrada G, Jacob S.<br>Pract Neurol. 2016 Aug;16(4):326-7.
Abdul-Rahman - Palmer, 2016	Anti-N-methyl-D-aspartate receptor encephalitis with an imaging-invisible ovarian teratoma: a case report.<br>Abdul-Rahman ZM, Panegyres PK, Roeck M, Hawkins D, Bharath J, Grolman P, Neppe C, Palmer D.<br>J Med Case Rep. 2016 Oct 24;10(1):296.
Abe -Yamamoto, 2016	Emergency Department Presentations of Anti-N-Methyl-D-Aspartate Receptor Encephalitis.<br>Abe KK, Koli RL, Yamamoto LG.<br>Pediatr Emerg Care. 2016 Feb;32(2):107-12; quiz 113-5.
Afanasiev - Psimaras, 2016	Anti-NMDA receptor antibody encephalitis and neuroendocrine pancreatic tumor: Causal link?<br>Afanasiev V, Brechemier ML, Boisseau W, Ducoudray R, Mayeur ME, Meyronet D, Peskine A, Desestret V, Psimaras D.<br>Neurology. 2016 Jul 5;87(1):112-3.
Agrawal - Wassmer, 2010	Successful treatment of antiN-methyl-d-aspartate receptor limbic encephalitis in a 22-monthold child with plasmapheresis and pharmacological immunomodulation.<br>Agrawal S, Vincent A, Jacobson L, Milford D, Gupta R, Wassmer E.<br>Arch Dis Child. 2010 Apr;95(4):312.
Aguiar de Sousa - Albuquerque, 2014	Pure ataxia associated with N-methyl-D-aspartate receptor antibodies.<br>Aguiar de Sousa D, Lobo PP, Caldas AC, Coelho M, Albuquerque L.<br>Parkinsonism Relat Disord. 2014 May;20(5):568-9.
Ahmad - Assad, 2017	Anti-n-Methyl-d-Aspartate-Receptor (NMDAR) Encephalitis in Association with Ovarian Teratoma.<br>Ahmad J, Sohail MS, Khan A, Qavi AH, Gaudel P, Zahid M, Assad S.<br>Cureus. 2017 Jul 5;9(7):e1425
Alexopoulos - Dalakas 2011	Paraneoplastic anti-NMDAR encephalitis: long term follow-up reveals persistent serum antibodies.<br>Alexopoulos H, Kosmidis ML, Dalmau J, Dalakas MC.<br>J Neurol. 2011 Aug;258(8):1568-70.
AlHakeem -Tabarki, 2016	Acute psychosis in children: do not miss immune-mediated causes.<br>AlHakeem AS, Mekki MS, AlShahwan SM, Tabarki BM.<br>Neurosciences (Riyadh). 2016 Jul;21(3):252-5.
Allen – Twomey, 2012	Limbic encephalitis in a boy with N-methyl-D-aspartate receptor antibodies.<br>Allen NM, Lynch B, Twomey E.<br>J Pediatr. 2012 Jun;160(6):1060.
Almuslamani - Mahmood, 2015	First Bahraini adolescent with anti-NMDAR-Ab encephalitis.<br>Almuslamani A, Mahmood F.<br>Qatar Med J. 2015 May 14;2015(1):2.
Alsaadi - Hamid, 2015	VNS terminating refractory nonconvulsive SE secondary to anti-NMDA encephalitis: A case report.<br>Alsaadi T, Shakra M, Turkawi L, Hamid J.<br>Epilepsy Behav Case Rep. 2015 May 15;3:39-42.
Amer -Kommineni, 2015	Refractory status epilepticus from NMDA receptor encephalitis successfully treated with an adjunctive ketogenic diet.<br>Amer S, Shah P, Kommineni V.<br>Ann Indian Acad Neurol. 2015 Apr-Jun;18(2):256-7.
Aoki – Inokuchi, 2012	Early diagnosis of anti-N-methyl-D-aspartate receptor encephalitis in a young woman with psychiatric symptoms.<br>Aoki H, Morita S, Miura N, Tsuji T, Ohnuki Y, Nakagawa Y, Yamamoto I, Takahashi H, Inokuchi S.<br>Tokai J Exp Clin Med. 2012 Sep 20;37(3):89-93.
Appu Noetzel, 2014	Clinically significant response to zolpidem in disorders of consciousness secondary to anti-N-methyl-D-aspartate receptor encephalitis in a teenager: a case report.<br>Appu M, Noetzel M.<br>Pediatr Neurol. 2014 Mar;50(3):262-4.
Arboleya - Julià, 2016	Anti-NMDAR antibodies in new-onset psychosis. Positive results in an HIV-infected patient.<br>Arboleya S, Clemente A, Deng S, Bedmar M, Salvador I, Herbera P, Cunill V, Vives-Bauza C, Haro JM, Canellas F, Julià MR.<br>Brain Behav Immun. 2016 Aug;56:56-60.
Armangue, Titulaer - Dalmau, 2013	Pediatric anti-N-methyl-D-aspartate receptor encephalitis-clinical analysis and novel findings in a series of 20 patients.<br>Armangue T, Titulaer MJ, Málaga I, Bataller L, Gabilondo I, Graus F, Dalmau J; Spanish Anti-N-methyl-D-Aspartate Receptor (NMDAR) Encephalitis Work Group.<br>J Pediatr. 2013 Apr;162(4):850-856.e2.
Arshad - Zahid, 2018	Anti-NMDA Receptor Encephalitis: A Masquerade Ball of Neuropsychiatric Symptoms.<br>Arshad A, Nabi S, Zahid M.<br>J Coll Physicians Surg Pak. 2018 Feb;28(2):173.
Arteche Andrés - Azcona Calahorra, 2015	[Anti-N-methyl-D-aspartate receptor encephalitis associated with ovarian teratoma: Description of a case and anesthetic implications].<br>Arteche Andrés MA, Zugasti Echarte O, de Carlos Errea J, Pérez Rodríguez M, Leyún Pérez de Zabalza R, Azcona Calahorra MA.<br>Rev Esp Anestesiol Reanim. 2015 Oct;62(8):468-71.

© 2021 Nosadini M et al. *JAMA Neurology*.

Asai - Iwata, 2011	Laparoscopic cystectomy of ovarian teratoma in anti-NMDAR encephalitis: 2 case reports.<br>Asai S, Ishimoto H, Yabuno A, Asada H, Seki M, Iwata S.<br>J Minim Invasive Gynecol. 2011 Jan-Feb;18(1):135-7.
Atmaca - Gurses, 2017	Investigation of anti-neuronal antibodies in status epilepticus of unknown etiology: a prospective study.<br>Atmaca MM, Tuzun E, Erdag E, Bebek N, Baykan B, Gurses C.<br>Acta Neurol Belg. 2017 Dec;117(4):841-848.
Aung - Grageda, 2017	Case 1: Acute Psychosis in a 16-year-old Girl with an Ovarian Teratoma.<br>Aung T, Ahuja N, Graziano J, Grageda M.<br>Pediatr Rev. 2017 Oct;38(10):487.
Azizyan - Moser, 2014	Anti-NMDA encephalitis: an uncommon, autoimmune mediated form of encephalitis.<br>Azizyan A, Albrektson JR, Maya MM, Pressman BD, Moser F.<br>J Radiol Case Rep. 2014 Aug 31;8(8):1-6. doi: 10.3941/jrcr.v8i8.1566. eCollection 2014 Aug.
Baheerathan - Trip, 2017	Antecedent anti-NMDA receptor encephalitis in two patients with multiple sclerosis.<br>Baheerathan A, Brownlee WJ, Chard DT, Shields K, Gregory R, Trip SA.<br>Mult Scler Relat Disord. 2017 Feb;12:20-22.
Baizabal-Carvallo - Jankovic, 2013	The spectrum of movement disorders in children with anti-NMDA receptor encephalitis.<br>Baizabal-Carvallo JF, Stocco A, Muscal E, Jankovic J.<br>Mov Disord. 2013 Apr;28(4):543-7.
Baker - Osborn, 2016	Two Cases of Anti-NMDA Receptor Encephalitis.<br>Baker J, Jeziorkowski C, Siebe C, Osborn MB.<br>West J Emerg Med. 2016 Sep;17(5):623-6.
Barros - Figueiroa, 2014	Resective surgery in the treatment of super-refractory partial status epilepticus secondary to NMDAR antibody encephalitis.<br>Barros P, Brito H, Ferreira PC, Ramalheira J, Lopes J, Rangel R, Temudo T, Figueiroa S.<br>Eur J Paediatr Neurol. 2014 May;18(3):449-52.
Barry - Murphy, 2011	Anti-NMDA receptor encephalitis: an important differential diagnosis in psychosis.<br>Barry H, Hardiman O, Healy DG, Keogan M, Moroney J, Molnar PP, Cotter DR, Murphy KC.<br>Br J Psychiatry. 2011 Dec;199(6):508-9.
Bashiri - Salih, 2017	Auto-immune anti-N-methyl-D-aspartate receptor (anti-NMDAR) encephalitis: three case reports.<br>Bashiri FA, Al-Rasheed AA, Hassan SM, Hamad MHA, El Khashab HY, Kentab AY, AlBadr FB, Salih MA.<br>Paediatr Int Child Health. 2017 Aug;37(3):222-226.
Batra – Friedman, 2012	Therapeutic plasma exchange in anti-N-methyl-D-aspartate-receptor (anti-NMDA-R) encephalitis associated with benign ovarian teratoma.<br>Batra R, Pang Y, Friedman MT.<br>J Clin Apher. 2012;27(4):227-8
Bayreuther – Thomas, 2009	Complex partial status epilepticus revealing anti-NMDA receptor encephalitis.<br>Bayreuther C, Bourg V, Dellamonica J, Borg M, Bernardin G, Thomas P.<br>Epileptic Disord. 2009 Sep;11(3):261-5.
Baysal-Kirac - Baykan, 2016	Neuronal autoantibodies in epilepsy patients with peri-ictal autonomic findings.<br>Baysal-Kirac L, Tuzun E, Erdag E, Ulusoy C, Vanli-Yavuz EN, Ekizoglu E, Peach S, Sezgin M, Bebek N, Gurses C, Gokyigit A, Vincent A, Baykan B.<br>J Neurol. 2016 Mar;263(3):455-66.
Baysal-Kirac - Baykan, 2016	Are There Any Specific EEG Findings in Autoimmune Epilepsies?<br>Baysal-Kirac L, Tuzun E, Altindag E, Ekizoglu E, Kinay D, Bilgic B, Tekturk P, Baykan B.<br>Clin EEG Neurosci. 2016 Jul;47(3):224-34.
Beatty - Khot, 2014	The diagnostic conundrum and treatment dilemma of a patient with a rapidly progressive encephalopathy.<br>Beatty CW, Creutzfeldt CJ, Davis AP, Hoffer Z, Khot SP.<br>Neurohospitalist. 2014 Jan;4(1):34-41. doi: 10.1177/1941874413496792. No abstract available.
Beaudonnet - Chalvon-Demersay, 2012	[Anti-NMDA receptor encephalitis: an underestimated cause of acute psychiatric syndrome in children and young adults].<br>Beaudonnet F, Garrec N, Sfez A, Epain V, Secret I, Angenard F, Wafo E, Deiva K, Chalvon-Demersay A.<br>Presse Med. 2012 Mar;41(3 Pt 1):318-20.
Beecher – Smyth, 2018	Teaching NeuroImages: Prosopagnosia heralding anti-NMDA receptor encephalitis.<br>Beecher G, Wagner AN, Abele J, Smyth P.<br>Neurology. 2018 May 29;90(22):e2012-e2013.
Behrendt - Kleiter, 2016	Bortezomib Treatment for Patients With Anti-N-Methyl-d-Aspartate Receptor Encephalitis.<br>Behrendt V, Krogias C, Reinacher-Schick A, Gold R, Kleiter I.<br>JAMA Neurol. 2016 Oct 1;73(10):1251-1253.
Ben Achour - Gouider-Khouja, 2013	Anti-NMDA receptor encephalitis mimicking a primary psychiatric disorder in a 13-year-old girl.<br>Ben Achour N, Ben Youssef-Turki I, Messelmani M, Kraoua I, Yaacoubi J, Klaa H, Rouissi A, Benrhouma H, Ben Ahmed M, Didelot A, Ducray F, Gouider-Khouja N.<br>Turk Psikiyatri Derg. 2013 Summer;24(2):145-7.
Ben Azoun - Blanc, 2014	[Two pediatric cases of anti-NMDA receptor antibody encephalitis].<br>Ben Azoun M, Tatencloux S, Deiva K, Blanc P.<br>Arch Pediatr. 2014 Nov;21(11):1216-9.
Benjumea-Cuartas - Kaminska, 2017	Unilateral predominance of abnormal movements: A characteristic feature of the pediatric anti-NMDA receptor encephalitis?<br>Benjumea-Cuartas V, Eisermann M, Simonnet H, Hully M, Nabbout R, Desguerre I, Kaminska A.<br>Epilepsy Behav Case Rep. 2017 Jan 18;7:42-44.

© 2021 Nosadini M et al. *JAMA Neurology*.

Beretta - Salmaggi, 2018	A Case of Anti-N-Methyl-D-Aspartate Receptor Encephalitis Associated with Glioma of the Pons.<br>Beretta F, Aliprandi A, Di Leo C, Salmaggi A.<br>J Clin Neurol. 2018 Oct 26. [Epub ahead of print] No abstract available.
Berg - Coffey, 2015	Neuroleptic Malignant Syndrome in a Boy with NMDA Receptor Encephalitis.<br>Berg A, Byrne R, Coffey BJ.<br>J Child Adolesc Psychopharmacol. 2015 May;25(4):368-71.
Bergink - Kushner, 2015	Autoimmune Encephalitis in Postpartum Psychosis.<br>Bergink V, Armangue T, Titulaer MJ, Markx S, Dalmau J, Kushner SA.<br>Am J Psychiatry. 2015 Sep 1;172(9):901-8.
Bien – Bauer, 2012	Immunopathology of autoantibody-associated encephalitides: clues for pathogenesis.<br>Bien CG, Vincent A, Barnett MH, Becker AJ, Blümcke I, Graus F, Jellinger KA, Reuss DE, Ribalta T, Schlegel J, Sutton I, Lassmann H, Bauer J.<br>Brain. 2012 May;135(Pt 5):1622-38.
Blitshteyn - Brook, 2017	Postural tachycardia syndrome (POTS) with anti-NMDA receptor antibodies after human papillomavirus vaccination.<br>Blitshteyn S, Brook J.<br>Immunol Res. 2017 Feb;65(1):282-284.
Boangher -Goffette, 2016	Small-Cell Lung Cancer with Positive Anti-NMDAR and Anti-AMPAR Antibodies Paraneoplastic Limbic Encephalitis.<br>Boangher S, Mespouille P, Filip CM, Goffette S.<br>Case Rep Neurol Med. 2016;2016:3263718.
Boeck - Stangel, 2013	Ovarectomy despite Negative Imaging in Anti-NMDA Receptor Encephalitis: Effective Even Late.<br>Boeck AL, Logemann F, Krauß T, Hussein K, Bültmann E, Trebst C, Stangel M.<br>Case Rep Neurol Med. 2013;2013:843192
Borlot - Reed, 2012	Anti-N-methyl D-aspartate receptor encephalitis in childhood.<br>Borlot F, Santos ML, Bandeira M, Liberalesso PB, Kok F, Löhr A Jr, Reed UC.<br>J Pediatr (Rio J). 2012 May;88(3):275-8.
Bost - Honnorat, 2018	Malignant tumors in autoimmune encephalitis with anti-NMDA receptor antibodies.<br>Bost C, Chanson E, Picard G, Meyronet D, Mayeur ME, Ducray F, Rogemond V, Psimaras D, Antoine JC, Delattre JY, Desestret V, Honnorat J.<br>J Neurol. 2018 Jul 12.
Bowes - Shannon Weickert, 2015	Anti-N-methyl-D-aspartate encephalitis - a case study of symptomatic progression.<br>Bowes E, Levy F, Lawson J, Mandalis A, Mohan A, Shannon Weickert C.<br>Australas Psychiatry. 2015 Aug;23(4):422-5
Braakman – Nicolai, 2010	Pearls & Oy-sters: electroconvulsive therapy in anti-NMDA receptor encephalitis.<br>Braakman HM, Moers-Hornikx VM, Arts BM, Hupperts RM, Nicolai J.<br>Neurology. 2010 Sep 7;75(10):e44-6.
Bradley, 2015	Rehabilitation following anti-NMDA encephalitis.<br>Bradley L.<br>Brain Inj. 2015;29(6):785-8.
Bravo-Oro - Campos-Guevara, 2013	[Anti-N-methyl-D-aspartate (NMDA) receptor encephalitis: experience with six pediatric patients. Potential efficacy of methotrexate].<br>Bravo-Oro A, Abud-Mendoza C, Quezada-Corona A, Dalmau J, Campos-Guevara V.<br>Rev Neurol. 2013 Nov 1;57(9):405-10.
Bravo-Oro - Reyes-Vaca, 2015	[Reversible cortical atrophy secondary to anti-NMDA receptor antibody encephalitis].<br>Bravo-Oro A, Acosta-Yebra D, Grimaldo-Zapata IP, Reyes-Vaca G.<br>Rev Neurol. 2015 May 16;60(10):447-52. Review. Spanish.
Breese – Sokol, 2010	Anti-N-methyl-D-aspartate receptor encephalitis: early treatment is beneficial.<br>Breese EH, Dalmau J, Lennon VA, Apiwattanakul M, Sokol DK.<br>Pediatr Neurol. 2010 Mar;42(3):213-4.
Brenton - Schwartz, 2016	Approach to the Management of Pediatric-Onset Anti-N-Methyl-d-Aspartate (Anti-NMDA) Receptor Encephalitis: A Case Series.<br>Brenton JN, Kim J, Schwartz RH.<br>J Child Neurol. 2016 Aug;31(9):1150-5.
Brier - Ances, 2016	N-methyl-D-aspartate receptor encephalitis mediates loss of intrinsic activity measured by functional MRI.<br>Brier MR, Day GS, Snyder AZ, Tanenbaum AB, Ances BM.<br>J Neurol. 2016 Jun;263(6):1083-91.
Broderick - Nanji, 2014	Total Intravenous Anesthesia Using N-Methyl-D-Aspartate (NMDA) Receptor-Sparing Drugs in a Patient with Anti-NMDA Receptor Encephalitis.<br>Broderick DK, Raines DE, Nanji KC.<br>A A Case Rep. 2014 Apr 1;2(7):83-5.
Brozzi - Zava, 2015	Anti-N-metil-D-aspartate receptor encephalitis: a challenge for intensivists.<br>Brozzi MK, Vissani M, Giuliani M, Pellegrino V, Peccini L, Luci R, Cascelli M, Nicoletta G, Lentischio L, Stefanucci S, Zava R.<br>Minerva Anestesiol. 2015 Oct;81(10):1146-7.
Bseikri - Roy-Burman, 2012	Anti-N-methyl D-aspartate receptor encephalitis mimics viral encephalitis.<br>Bseikri MR, Barton JR, Kulhanjian JA, Dalmau J, Cohen RA, Glaser CA, Roy-Burman A.<br>Pediatr Infect Dis J. 2012 Feb;31(2):202-4.

© 2021 Nosadini M et al. *JAMA Neurology*.

Bushman – King, 2011	Nine-year-old girl with altered mental status.<br>Bushman ET, Mellion SA, King MA.<br>Clin Pediatr (Phila). 2011 Dec;50(12):1161-3.
Bustos - Ortiz, 2017	[Autoimmune encephalitis associated to antibodies against the N-methyl-D-aspartate receptor: Report of two cases].<br>Bustos J, Sánchez Y, Medina J, Olivieri R, Mojica J, Ortiz J.<br>Biomedica. 2017 Apr 1;37(0):20-25.
Byrne - King, 2014	Does early treatment improve outcomes in N-methyl-D-aspartate receptor encephalitis?<br>Byrne S, McCoy B, Lynch B, Webb D, King MD.<br>Dev Med Child Neurol. 2014 Aug;56(8):794-6.
Caballero, 2011	Fluorodeoxyglucose positron emission tomography findings in NMDA receptor antibody encephalitis.<br>Caballero PE.<br>Arq Neuropsiquiatr. 2011;69(2B):409-10.
Cai, Zhou - Ren, 2018	Anti-N-methyl-D-aspartate receptor encephalitis associated with acute Toxoplasma gondii infection: A case report.<br>Cai X, Zhou H, Xie Y, Yu D, Wang Z, Ren H.<br>Medicine (Baltimore). 2018 Feb;97(7):e9924.
Camdessanché – Antoine, 2011	Brain immunohistopathological study in a patient with anti-NMDAR encephalitis.<br>Camdessanché JP, Streichenberger N, Cavillon G, Rogemond V, Jousserand G, Honnorat J, Convers P, Antoine JC.<br>Eur J Neurol. 2011 Jun;18(6):929-31.
Casanova-Gracia - Cortina-Lacambra, 2012	Encephalitis associated to anti-NMDA receptor antibodies: a description of two cases in the child/youth population].<br>Casanova-Gracia N, Banzo-Arguis C, Sanz-Asin P, Zapata-Usabel M, Jordana-Vilanova N, Cortina-Lacambra MT.<br>Rev Neurol. 2012 Apr 16;54(8):475-8. Spanish.
Cassa – Rosengart, 2013	A young man with altered mental status and new-onset seizures.<br>Cassa RS, Rosengart AJ.<br>JAAPA. 2013 Oct;26(10):34-7.
Castellano -Robinson, 2017	Extreme Delta Brush in NMDA Receptor Encephalitis.<br>Castellano J, Glover R, Robinson J.<br>Neurohospitalist. 2017 Jul;7(3):NP3-NP4.
Chakrabarty - Rathi, 2014	Pediatric anti-N-methyl-D-aspartate (NMDA) receptor encephalitis: experience of a tertiary care teaching center from north India.<br>Chakrabarty B, Tripathi M, Gulati S, Yoganathan S, Pandit AK, Sinha A, Rathi BS.<br>J Child Neurol. 2014 Nov;29(11):1453-9.
Chan – Vincent, 2010	Anti-NMDA receptor encephalitis with atypical brain changes on MRI.<br>Chan SH, Wong VC, Fung CW, Dale RC, Vincent A.<br>Pediatr Neurol. 2010 Oct;43(4):274-8.
Chan - Lynch, 2015	A rare case of anti-N-methyl-D-aspartate receptor encephalitis during pregnancy.<br>Chan LW, Nilsson C, Schepel J, Lynch C.<br>N Z Med J. 2015 Mar 27;128(1411):89-91.
Chanson – Hirsch, 2012	PET follow-up in a case of anti-NMDAR encephalitis: arguments for cingulate limbic encephalitis.<br>Chanson JB, Diaconu M, Honnorat J, Martin T, De Seze J, Namer IJ, Hirsch E.<br>Epileptic Disord. 2012 Mar;14(1):90-3.
Chanson - Rosenberg, 2016	Anti-NMDA-R encephalitis: Should we consider extreme delta brush as electrical status epilepticus?<br>Chanson E, Bicilli É, Lauxerois M, Kauffmann S, Chabanne R, Ducray F, Honnorat J, Clavelou P, Rosenberg S.<br>Neurophysiol Clin. 2016 Feb;46(1):17-25.
Chapman – Vause, 2011	Anti-NMDA receptor encephalitis: diagnosis, psychiatric presentation, and treatment.<br>Chapman MR, Vause HE.<br>Am J Psychiatry. 2011 Mar;168(3):245-51.
Chatterjee - Mitra, 2017	Psychosis and catatonia as presenting features of anti-N-methyl-D-aspartate (anti-NMDA) receptor encephalitis.<br>Chatterjee SS, Ghosal MK, Mitra S.<br>Asian J Psychiatr. 2017 Jun;27:112.
Chawla -Abbate, 2016	Autonomic instability and asystole: Broadening the differential diagnosis of cardiac arrhythmias.<br>Chawla R, Zukas AM, Pitcher JH, Trankle C, Brath L, Abbate A.<br>Int J Cardiol. 2016 Oct 1;220:665-7.
Chen - Mao, 2014	Marked improvement of anti-N-methyl-D-aspartate receptor encephalitis by large-dose methylprednisolone and plasmapheresis therapy combined with 18F-fluorodeoxyglucose positron emission tomography imaging: A case report.<br>Chen B, Wang Y, Geng Y, Huang Y, Guo S, Mao X.<br>Exp Ther Med. 2014 Oct;8(4):1167-1169.
Chi, Wang -Zhou, 2017	Risk factors for mortality in patients with anti-NMDA receptor encephalitis.<br>Chi X, Wang W, Huang C, Wu M, Zhang L, Li J, Zhou D.<br>Acta Neurol Scand. 2017 Oct;136(4):298-304.
Chia - Foo, 2011	Profound sinus node dysfunction in anti-N-methyl-d-aspartate receptor limbic encephalitis.<br>Chia PL, Tan K, Foo D.<br>Pacing Clin Electrophysiol. 2013 Mar;36(3):e90-2
Chiang – Goldsmith, 2018	Pearls & Oy-sters: Relapse of anti-NMDA receptor encephalitis after prior first- and second-line immunotherapy.<br>Chiang S, Garg T, Hu A, Amin H, Davalos-Balderas A, Alfradique-Dunham I, Goldsmith CE.<br>Neurology. 2018 May 15;90(20):936-939.
Choe – Mulert, 2013	A clinical and neurobiological case of IgM NMDA receptor antibody associated encephalitis mimicking bipolar disorder.

© 2021 Nosadini M et al. *JAMA Neurology*.

	Choe CU, Karamatskos E, Schattling B, Leypoldt F, Liuzzi G, Gerloff C, Friese MA, Mulert C.<br>Psychiatry Res. 2013 Jul 30;208(2):194-6.
Chourasia - Kamdar, 2018	An Infant Born to a Mother With Anti-N-Methyl-d-Aspartate Receptor Encephalitis.<br>Chourasia N, Watkins MW, Lankford JE, Kass JS, Kamdar A.<br>Pediatr Neurol. 2018 Feb;79:65-68.
Clara - Oller, 2016	The Enemy Within: Anti-N-Methyl D-Aspartate Receptor Encephalitis.<br>Clara JA, Kalan SP, Oller KL.<br>Am J Med. 2016 Oct;129(10):e219-21.
Cleland - Rahm, 2015	A 16-year-old girl with anti-NMDA-receptor encephalitis and family history of psychotic disorders.<br>Cleland N, Lieblich S, Schalling M, Rahm C.<br>Acta Neuropsychiatr. 2015 Dec;27(6):375-9.
Cleverly - Navaratnarajah, 2014	Paraneoplastic anti-N-methyl-D-aspartate receptor encephalitis: have you checked the ovaries?<br>Cleverly K, Gambadauro P, Navaratnarajah R.<br>Acta Obstet Gynecol Scand. 2014 Jul;93(7):712-5.
Çoban - Gürvit, 2016	NMDA receptor encephalitis with cancer of unknown primary origin.<br>Çoban A, Gündoğdu G, Poyraz M, Yegen G, Demirtaş-Tatlıdede A, Bilgiç B, Hanagasi HA, Tüzün E, Gürvit H.<br>Tumori. 2016 Nov 11;102(Suppl. 2).
Cohen - Wong-Kisiel, 2014	Case of a two-year-old boy with recurrent seizures, abnormal movements, and central hypoventilation.<br>Cohen AL, Wong-Kisiel LC.<br>Semin Pediatr Neurol. 2014 Jun;21(2):114-8.
Colley - Smith, 2014	Sore eyes and psychosis.<br>Colley S, Smith J.<br>BMJ Case Rep. 2014 Sep 5;2014.
Consoli – Cohen, 2011	Malignant catatonia due to anti-NMDA-receptor encephalitis in a 17-year-old girl: case report.<br>Consoli A, Ronen K, An-Gourfinkel I, Barbeau M, Marra D, Costedoat-Chalumeau N, Montefiore D, Maksud P, Bonnot O, Didelot A, Amoura Z, Vidailhet M, Cohen D.<br>Child Adolesc Psychiatry Ment Health. 2011 May 13;5(1):15.
Constantinides - Potagas, 2018	Anti-NMDA receptor encephalitis presenting as isolated aphasia in an adult.<br>Constantinides VC, Kasselimis DS, Paraskevas GP, Zacharopoulou M, Andreadou E, Evangelopoulos ME, Kapaki E, Kilidireas C, Stamboulis E, Potagas C.<br>Neurocase. 2018 Oct 6:1-7
Creten – Schieveld, 2011	Late onset autism and anti-NMDA-receptor encephalitis.<br>Creten C, van der Zwaan S, Blankespoor RJ, Maatkamp A, Nicolai J, van Os J, Schieveld JN.<br>Lancet. 2011 Jul 2;378(9785):98.
Cuende - Ruiz, 2015	Anti-NMDA receptor encephalitis in a patient with rheumatoid arthritis.<br>Cuende E, Ruiz L.<br>J Rheumatol. 2015 Jan;42(1):140.
Cundiff - Shehata, 2015	Does MAP2 have a role in predicting the development of anti-NMDAR encephalitis associated with benign ovarian teratoma? A report of six new pediatric cases.<br>Cundiff CA, Elawabdeh N, Naguib MM, Jactel SN, Demellawy DE, Abramowsky CR, Durham MM, Youssef L, Wittkamp ML, Shehata BM.<br>Pediatr Dev Pathol. 2015 Mar-Apr;18(2):122-6.
da Silva-Júnior - Garzon, 2014	Serial and prolonged EEG monitoring in anti-N-Methyl-d-Aspartate receptor encephalitis.<br>da Silva-Júnior FP, Castro LH, Andrade JQ, Bastos CG, Moreira CH, Valério RM, Jorge CL, Marchiori PE, Nitrini R, Garzon E.<br>Clin Neurophysiol. 2014 Aug;125(8):1541-4.
Dabner - Stewart, 2012	Ovarian teratoma associated with anti-N-methyl D-aspartate receptor encephalitis: a report of 5 cases documenting prominent intratumoral lymphoid infiltrates.<br>Dabner M, McCluggage WG, Bundell C, Carr A, Leung Y, Sharma R, Stewart CJ.<br>Int J Gynecol Pathol. 2012 Sep;31(5):429-37.
Dale, Brilot - Lim, 2014	Utility and safety of rituximab in pediatric autoimmune and inflammatory CNS disease.<br>Dale RC, Brilot F, Duffy LV, Twilt M, Waldman AT, Narula S, Muscal E, Deiva K, Andersen E, Eyre MR, Eleftheriou D, Brogan PA, Kneen R, Alper G, Anlar B, Wassmer E, Heineman K, Hemingway C, Riney CJ, Kornberg A, Tardieu M, Stocco A, Banwell B, Gorman MP, Benseler SM, Lim M.<br>Neurology. 2014 Jul 8;83(2):142-50.
Dale, Pillai, Brilot, 2013	Cerebrospinal fluid CD19(+) B-cell expansion in N-methyl-D-aspartate receptor encephalitis.<br>Dale RC, Pillai S, Brilot F.<br>Dev Med Child Neurol. 2013 Feb;55(2):191-3.
Dalmau - Lynch, 2007	Paraneoplastic anti-N-methyl-D-aspartate receptor encephalitis associated with ovarian teratoma.<br>Dalmau J, Tüzün E, Wu HY, Masjuan J, Rossi JE, Voloschin A, Baehring JM, Shimazaki H, Koide R, King D, Mason W, Sansing LH, Dichter MA, Rosenfeld MR, Lynch DR.<br>Ann Neurol. 2007 Jan;61(1):25-36.
Dalmau – Saiz, 2013	[A 40-year-old woman with headache and diplopia].<br>Dalmau J, Berenguer J, Saiz A.<br>Med Clin (Barc). 2013 Dec 21;141(12):543-9. doi: 10.1016/j.medcli.2013.07.003. Epub 2013 Sep 9. Spanish. No abstract available.
Danieli - Masruha, 2017	Anti-N-methyl-D-aspartate receptor encephalitis and Epstein-Barr virus: another tale on autoimmunity?<br>Danieli D, Moraes ACM, Alves MP, Dutra LA, Höftberger R, Barsottini OGP, Masruha MR.<br>Eur J Neurol. 2017 Aug;24(8):e46-e47.

© 2021 Nosadini M et al. *JAMA Neurology*.

Davies - Kullmann, 2010	Anti-N-methyl-D-aspartate receptor antibodies: a potentially treatable cause of encephalitis in the intensive care unit.<br>Davies G, Irani SR, Coltart C, Ingle G, Amin Y, Taylor C, Radcliffe J, Hirsch NP, Howard RS, Vincent A, Kullmann DM.<br>Crit Care Med. 2010 Feb;38(2):679-82.
Day - Andrade, 2015	GRIN1 polymorphisms do not affect susceptibility or phenotype in NMDA receptor encephalitis.<br>Day GS, Prüss H, Benseler SM, Paton TA, Paterson AD, Andrade DM.<br>Neurol Neuroimmunol Neuroinflamm. 2015 Sep 24;2(5):e153.
Day - Munoz, 2014	Abnormal neurons in teratomas in NMDAR encephalitis.<br>Day GS, Laiq S, Tang-Wai DF, Munoz DG.<br>JAMA Neurol. 2014 Jun;71(6):717-24.
Day - Tang-Wai, 2011	Anti-NMDA-receptor encephalitis: case report and literature review of an under-recognized condition.<br>Day GS, High SM, Cot B, Tang-Wai DF.<br>J Gen Intern Med. 2011 Jul;26(7):811-6.
De Ciervo - Finvarb, 2017	[N-Methyl-D-Aspartate receptor encephalitis: An adolescent case report and literature review. How to manage neuropsychiatric symptoms].<br>De Ciervo F, Willimburgh V, Finvarb G.<br>Vertex. 2017 Mar;28(132):128-135. Spanish.
De Leu – Velkeniers, 2012	Seizures and postictal stupor in a patient with uncontrolled Graves' hyperthyroidism.<br>De Leu N, Unuane D, Poppe K, Velkeniers B.<br>BMJ Case Rep. 2012 Jun 1;2012. pii: bcr0220125929.
De Maeseneire -Santos , 2017	A case of anti-NMDA receptor encephalitis revealed by insular epilepsy.<br>De Maeseneire C, Tahry RE, Santos SF.<br>Epileptic Disord. 2017 Dec 1;19(4):471-475.
De Nayer – Sindic, 2009	A subacute behavioral disorder in a female adolescent. Autoimmune anti-N-methyl-D-aspartate receptor encephalitis associated with ovarian teratoma.<br>De Nayer AR, Myant N, Sindic CJ.<br>Biol Psychiatry. 2009 Sep 15;66(6):e13-4.
De Scheerder - Vogelaers, 2016	How far to investigate presumed psychosomatic symptoms: Lessons from a particular case….<br>De Scheerder MA, Rottey S, Mariman A, Praet M, Vogelaers D.<br>Acta Clin Belg. 2017 Apr;72(2):138-141.
Dean - Farrugia, 2012	End of the bed (end of the video) diagnosis.<br>Dean Z, Vincent A, Farrugia ME.<br>Pract Neurol. 2012 Apr;12(2):135-8.
Deiva - Tardieu, 2014	Sudden and isolated Broca's aphasia: a new clinical phenotype of anti NMDA receptor antibodies encephalitis in children.<br>Deiva K, Pera MC, Maurey H, Chrétien P, Archambaud F, Bouilleret V, Tardieu M.<br>Eur J Paediatr Neurol. 2014 Nov;18(6):790-2.
Delgado-García -Flores-Rivera, 2018	Opisthotonus (arc de cercle) in anti-NMDAR encephalitis.<br>Delgado-García G, Cano-Nigenda V, Abundes-Corona A, Carrillo-Loza K, Calleja-Castillo J, Flores-Rivera J.<br>Arq Neuropsiquiatr. 2018 Jun;76(6):426.
Demma - Dolak, 2017	Neuraxial anesthesia in a patient with anti-N-methyl-D-aspartate receptor encephalitis in pregnancy: management for cesarean delivery and oophorectomy.<br>Demma L, Norris S, Dolak J.<br>Int J Obstet Anesth. 2017 May;31:104-107.
Dengler - Seifi, 2017	Tramadol may increase the efficacy of therapeutic plasma exchange in anti-NMDAR encephalitis.<br>Dengler BA, Kitchen D, Seifi A.<br>Clin Neurol Neurosurg. 2017 Sep;160:38-39.
Dericioglu - Topcuoglu, 2013	Antiepileptic treatment for anti-NMDA receptor encephalitis: the need for video-EEG monitoring.<br>Dericioglu N, Vural A, Acar P, Agayeva N, Ismailova V, Kurne A, Saka E, Arsava EM, Topcuoglu MA.<br>Epileptic Disord. 2013 Jun;15(2):166-70.
DeSena – Graves (Light switch), 2014<br>DeSena – Graves (Three phenotypes), 2014	"Light switch" mental status changes and irritable insomnia are two particularly salient features of anti-NMDA receptor antibody encephalitis.<br>DeSena AD, Greenberg BM, Graves D.<br>Pediatr Neurol. 2014 Jul;51(1):151-3.
DeSena – Graves (Three phenotypes), 2014	Three phenotypes of anti-N-methyl-D-aspartate receptor antibody encephalitis in children: prevalence of symptoms and prognosis.<br>DeSena AD, Greenberg BM, Graves D.<br>Pediatr Neurol. 2014 Oct;51(4):542-9. doi: 10.1016/j.pediatrneurol.2014.04.030.
DeSena, Noland - Graves, 2015	Intravenous methylprednisolone versus therapeutic plasma exchange for treatment of anti-N-methyl-D-aspartate receptor antibody encephalitis: A retrospective review.<br>DeSena AD, Noland DK, Matevosyan K, King K, Phillips L, Qureshi SS, Greenberg BM, Graves D.<br>J Clin Apher. 2015 Aug;30(4):212-6.
Di Capua - García-Morales, 2013	Extreme delta brush in a patient with anti-NMDAR encephalitis.<br>Di Capua D, García-Ptacek S, García-García ME, Abarrategui B, Porta-Etessam J, García-Morales I.<br>Epileptic Disord. 2013 Dec;15(4):461-4.
Ding - Son, 2017	Case report: anaesthetic management of radical gastrectomy for gastric cancer associated with anti-N-methyl-D-aspartate receptor encephalitis.

© 2021 Nosadini M et al. *JAMA Neurology*.

	Ding L, Tan H, Li Z, Ji J, Song X.<br>BMC Anesthesiol. 2017 Jul 6;17(1):90.
do Valle - Scola, 2018	Clinical variability of children with anti-N-methyl-D-aspartate receptor encephalitis in southern Brazil: a cases series and review of the literature.<br>do Valle DA, Galeazzi JSP, Machado MR, Dos Santos VCSAR, da Silva AF, Lohr Júnior A, Santos MLSF, Scola RH.<br>Neurol Sci. 2018 Nov 20. doi: 10.1007/s10072-018-3648-z. [Epub ahead of print]<br>PMID: 30460460
Doden - Ikeda, 2017	Postpartum Anti-N-methyl-D-aspartate Receptor Encephalitis: A Case Report and Literature Review.<br>Doden T, Sekijima Y, Ikeda J, Ozawa K, Ohashi N, Kodaira M, Hineno A, Tachibana N, Ikeda SI.<br>Intern Med. 2017;56(3):357-362.
Dogan Onugoren -Bien, 2016	Immunoadsorption therapy in autoimmune encephalitides.<br>Dogan Onugoren M, Golombeck KS, Bien C, Abu-Tair M, Brand M, Bulla-Hellwig M, Lohmann H, Münstermann D, Pavenstädt H, Thölking G, Valentin R, Wiendl H, Melzer N, Bien CG.<br>Neurol Neuroimmunol Neuroinflamm. 2016 Feb 26;3(2):e207.
Dou – Chen, 2012	Abnormal sensory-motor integration in a patient with anti-NMDA-receptor encephalitis.<br>Dou YH, Lai KL, Liao KK, Chen SP.<br>J Neurol. 2012 Jan 28.
Dulcey – Nogales, 2012	Necrotic mature ovarian teratoma associated with anti-N-methyl-D-aspartate receptor encephalitis.<br>Dulcey I, Céspedes MU, Ballesteros JL, Preda O, Aneiros-Fernández J, Clavero PA, Nogales FF.<br>Pathol Res Pract. 2012 Aug 15;208(8):497-500.
Eker	Testicular teratoma and anti-N-methyl-D-aspartate receptor-associated encephalitis.<br>Eker A, Saka E, Dalmau J, Kurne A, Bilen C, Ozen H, Ertoy D, Oguz KK, Elibol B.<br>J Neurol Neurosurg Psychiatry. 2008 Sep;79(9):1082-3.
Endres - van Elst, 2015	Hypoglutamatergic state is associated with reduced cerebral glucose metabolism in anti-NMDA receptor encephalitis: a case report.<br>Endres D, Perlov E, Stich O, Rauer S, Maier S, Waldkircher Z, Lange T, Mader I, Meyer PT, van Elst LT.<br>BMC Psychiatry. 2015 Aug 1;15:186.
Evoli – Marra, 2012	Spontaneous recovery from anti-NMDAR encephalitis.<br>Evoli A, Spinelli P, Frisullo G, Alboini PE, Servidei S, Marra C.<br>J Neurol. 2012 Sep;259(9):1964-6.
Fan , Xu - Cui, 2018	Comparison of myelin oligodendrocyte glycoprotein (MOG)-antibody disease and AQP4-IgG-positive neuromyelitis optica spectrum disorder (NMOSD) when they co-exist with anti-NMDA (N-methyl-D-aspartate) receptor encephalitis.<br>Fan S, Xu Y, Ren H, Guan H, Feng F, Gao X, Ding D, Fang F, Shan G, Guan T, Zhang Y, Dai Y, Yao M, Peng B, Zhu Y, Cui L.<br>Mult Scler Relat Disord. 2018 Feb;20:144-152.
Feigal - Preud'Homme, 2018	Anti-N-Methyl-d-Aspartate Receptor Encephalitis Presenting With Features of Kleine-Levin Syndrome and Demyelination.Feigal J, Lin C, Barrio G, Kranz PG, Preud'Homme X.<br>Psychosomatics. 2016 May-Jun;57(3):310-4. d
Ferioli – Espay, 2010	Anti-N-methyl-D-aspartate receptor encephalitis: characteristic behavioral and movement disorder.<br>Ferioli S, Dalmau J, Kobet CA, Zhai QJ, Broderick JP, Espay AJ.<br>Arch Neurol. 2010 Feb;67(2):250-1.
Fields – Coffey, 2013	A rare case of anti-N-methyl-D-aspartate receptor encephalitis in an adolescent.<br>Fields J, Lim T, Kolevzon A, Coffey BJ.<br>J Child Adolesc Psychopharmacol. 2013 Sep;23(7):502-6.
Filatenkov - Rajaram, 2017	Persistence of parenchymal and perivascular T-cells in treatment-refractory anti-N-methyl-D-aspartate receptor encephalitis.<br>Filatenkov A, Richardson TE, Daoud E, Johnson-Welch SF, Ramirez DM, Torrealba J, Greenberg B, Monson NL, Rajaram V.<br>Neuroreport. 2017 Sep 27;28(14):890-895.
Finke - Ruprecht, 2014	Anti-NMDAR encephalitis mimicking HaNDL syndrome.<br>Finke C, Mengel A, Prüss H, Stöcker W, Meisel A, Ruprecht K.<br>Cephalalgia. 2014 Oct;34(12):1012-4.
Finke - Ploner, 2012	Cognitive deficits following anti-NMDA receptor encephalitis.<br>Finke C, Kopp UA, Prüss H, Dalmau J, Wandinger KP, Ploner CJ.<br>J Neurol Neurosurg Psychiatry. 2012 Feb;83(2):195-8.
Finné Lenoir - Hantson, 2013	Anti-N-methyl-D-aspartate receptor encephalitis with favorable outcome despite prolonged status epilepticus.<br>Finné Lenoir X, Sindic C, van Pesch V, El Sankari S, de Tourtchaninoff M, Denays R, Hantson P.<br>Neurocrit Care. 2013 Feb;18(1):89-92.
Fisher - Tolby, 2017	Psychosis in the ED: A case of NMDA receptor antibody encephalitis.<br>Fisher J, Ellingson C, Tolby N.<br>Am J Emerg Med. 2017 Jul;35(7):1035.e5-1035.e6.
Fleischmann - Ruprecht, 2015	Severe cognitive impairment associated with intrathecal antibodies to the NR1 subunit of the N-methyl-D-aspartate receptor in a patient with multiple sclerosis.<br>Fleischmann R, Prüss H, Rosche B, Bahnemann M, Gelderblom H, Deuschle K, Harms L, Kopp U, Ruprecht K.<br>JAMA Neurol. 2015 Jan;72(1):96-9.
Florance, Davis - Dalmau, 2009	Anti-N-methyl-D-aspartate receptor (NMDAR) encephalitis in children and adolescents.<br>Florance NR, Davis RL, Lam C, Szperka C, Zhou L, Ahmad S, Campen CJ, Moss H, Peter N, Gleichman AJ,

© 2021 Nosadini M et al. *JAMA Neurology*.

	Glaser CA, Lynch DR, Rosenfeld MR, Dalmau J.<br>Ann Neurol. 2009 Jul;66(1):11-8.
Foff - Quigg, 2017	EEG Findings May Serve as a Potential Biomarker for Anti-NMDA Receptor Encephalitis.<br>Foff EP, Taplinger D, Suski J, Lopes MB, Quigg M.<br>Clin EEG Neurosci. 2017 Jan;48(1):48-53.
Frawley – McMasters, 2012	'Benign' ovarian teratoma and N-methyl-D-aspartate receptor (NMDAR) encephalitis in a child.<br>Frawley KJ, Calvo-Garcia MA, Krueger DA, McMasters RL.<br>Pediatr Radiol. 2012 Jan;42(1):120-3.
Frechette – Dalmau, 2011	Prolonged follow-up and CSF antibody titers in a patient with anti-NMDA receptor encephalitis.<br>Frechette ES, Zhou L, Galetta SL, Chen L, Dalmau J.<br>Neurology. 2011 Feb 15;76(7 Suppl 2):S64-6.
Freri - Granata, 2015	Focal seizure, focal dyskinesia, or both? A complex motor phenomenon reveals anti-NMDAR encephalitis.<br>Freri E, Matricardi S, Patrini M, Binelli S, Andreetta F, Teutonico F, Nardocci N, Granata T.<br>Seizure. 2015 Apr;27:16-8.
Frunza-Stefan - Malek, 2018	Unusual case of anti-N-methyl-D-aspartic acid-receptor (NMDA-R) encephalitis and autoimmune polyglandular syndrome (APS).<br>Frunza-Stefan S, Whitlatch HB, Rao GG, Malek R.<br>BMJ Case Rep. 2018 May 2;2018. pii: bcr-2018-224821.
Funayama - Mimura, 2018	Cotard's syndrome in anti-N-methyl-d-aspartate receptor encephalitis.<br>Funayama M, Takata T, Mimura M.<br>Psychiatry Clin Neurosci. 2018 Jun;72(6):455-456.
Gabilondo - Graus, 2011	Analysis of relapses in anti-NMDAR encephalitis.<br>Gabilondo I, Saiz A, Galán L, González V, Jadraque R, Sabater L, Sans A, Sempere A, Vela A, Villalobos F, Viñals M, Villoslada P, Graus F.<br>Neurology. 2011 Sep 6;77(10):996-9.
Gahr - Lewerenz, 2015	Periventricular white matter lesion and incomplete MRZ reaction in a male patient with anti-N-methyl-D-aspartate receptor encephalitis presenting with dysphoric mania.<br>Gahr M, Lauda F, Wigand ME, Connemann BJ, Rosenbohm A, Tumani H, Reindl M, Uzelac Z, Lewerenz J.<br>BMJ Case Rep. 2015 Apr 26;2015.
Garcia – Lorenzo-Bosquet, 2015	[Anti-NMDAR (N-methy-D-aspartate receptor) limbic encephalitis diagnosed by ^{18}F-FDG PET/CT].<br>Garcia JR, Saura J, Flor A, Soler M, Moragas M, Lorenzo-Bosquet C.<br>Rev Esp Med Nucl Imagen Mol. 2015 Mar-Apr;34(2):143-5. doi: 10.1016/j.remn.2014.06.001. Epub 2014 Jul 19. Spanish. No abstract available.
García-Ull - Masegosa, 2018	Anti-NMDA receptor encephalitis in an elderly patient: A case report.<br>García-Ull J, Cañizares-Ledo E, Gómez-Martínez J, Gómez IV, Masegosa AG.<br>Neurologia. 2018 Sep;33(7):482-483.
Garg - Gupta, 2019	Association of typhoid fever with anti-NMDAR encephalitis in a young child.<br>Garg M, Mittal J, Gupta P.<br>J Neuroimmunol. 2019 Mar 15;328:76-77.
Gastaldi - Franciotta, 2018	NMDAR encephalitis presenting as akinesia in a patient with Parkinson disease.<br>Gastaldi M, Arbasino C, Dallocchio C, Diamanti L, Bini P, Marchioni E, Franciotta D.<br>J Neuroimmunol. 2018 Dec 12;328:35-37.
Gataullina – Dulac, 2011	Paroxysmal EEG pattern in a child with N-methyl-D-aspartate receptor antibody encephalitis.<br>Gataullina S, Plouin P, Vincent A, Scalais E, Nuttin C, Dulac O.<br>Dev Med Child Neurol. 2011 Aug;53(8):764-7.
Gharedaghi – Ebrahimi, 2018	Anaesthetic management of a patient with a unique combination of anti-N-methyl-D-aspartate receptor encephalitis and stiff-person syndrome.<br>Gharedaghi MH, Khorasani A, Knezevic NN, Ebrahimi F.<br>BMJ Case Rep. 2018 May 7;2018.
Gitiaux - Kaminska, 2013	Early electro-clinical features may contribute to diagnosis of the anti-NMDA receptor encephalitis in children.<br>Gitiaux C, Simonnet H, Eisermann M, Leunen D, Dulac O, Nabbout R, Chevignard M, Honnorat J, Gataullina S, Musset L, Scalais E, Gauthier A, Hully M, Boddaert N, Kuchenbuch M, Desguerre I, Kaminska A.<br>Clin Neurophysiol. 2013 Dec;124(12):2354-61.
Goenka - Steinschneider, 2017	Extended Clinical Spectrum of Anti-N-Methyl-d-Aspartate Receptor Encephalitis in Children: A Case Series.<br>Goenka A, Jain V, Nariai H, Spiro A, Steinschneider M.<br>Pediatr Neurol. 2017 Jul;72:51-55.
Gold – Barone, 2010	Clinical Reasoning: An 18-year-old man with subacute mental status change.<br>Gold D, Dougherty R, Barone D.<br>Neurology. 2010 May 18;74(20):e83-6.
Goldberg – Ryan, 2014	Anti-N-methyl-D-aspartate receptor-mediated encephalitis in infants and toddlers: case report and review of the literature.<br>Goldberg EM, Titulaer M, de Blank PM, Sievert A, Ryan N.<br>Pediatr Neurol. 2014 Feb;50(2):181-4
Goldberg – Abend, 2011	Anti-NMDA receptor encephalitis presenting with focal non-convulsive status epilepticus in a child.<br>Goldberg EM, Taub KS, Kessler SK, Abend NS.<br>Neuropediatrics. 2011 Oct;42(5):188-90.
Goldberg - Cellucci, 2017	New Onset Insomnia in a Pediatric Patient: A Case of Anti-NMDA Receptor Encephalitis.<br>Goldberg TN, Cellucci MF.<br>Case Rep Pediatr. 2017;2017:4083785.

Gomes Ferreira - Doyague Sánchez, 2018	Successful treatment of anti-NMDA receptor encephalitis with early teratoma removal and plasmapheresis: A case report.<br>Gomes Ferreira M, Lapresa Alcalde V, García Sánchez MH, Hernández Hernández L, Doyague Sánchez MJ.<br>Medicine (Baltimore). 2018 Aug;97(31):e11325.
Gómez Castro - Conde, 2017	[Suspicion Index: Psychiatric Manifestations of NMDAR Encephalitis in Paediatric Patients].<br>Gómez Castro JF, Salazar O, Conde Z.<br>Rev Colomb Psiquiatr. 2017 Oct - Dec;46(4):252-256.
Gong - Huang, 2015	Potential Effect of Preoperative Immunotherapy on Anesthesia of Patients with Anti-N-methyl-D-aspartate Receptor Encephalitis.<br>Gong YH, Zhang MZ, Zhang XH, Guan HZ, Xu XQ, Huang YG.<br>Chin Med J (Engl). 2015 Nov 5;128(21):2972-5.
González-Latapi - González-Aguilar, 2014	[Anti-N-Methyl-D-aspartate receptor encephalitis (anti-NMDAR): a case report].<br>González-Latapi P, Rodríguez-Violante M, Cervantes-Arriaga A, Calleja-Castillo JM, González-Aguilar A.<br>Gac Med Mex. 2014 Jul-Aug;150(4):348-51. Spanish.
González-Toro - Gómez-Gosálvez, 2013	[Anti-NMDA receptor encephalitis: two paediatric cases].<br>González-Toro MC, Jadraque-Rodríguez R, Sempere-Pérez Á, Martínez-Pastor P, Jover-Cerdá J, Gómez-Gosálvez F.<br>Rev Neurol. 2013 Dec 1;57(11):504-8. Spanish.
González-Valcárcel - Dalmau, 2010	[Differential diagnosis of encephalitis due to anti-NMDA receptor antibodies].<br>González-Valcárcel J, Rosenfeld MR, Dalmau J.<br>Neurologia. 2010 Sep;25(7):409-13. Spanish.
Gorman - Thibert, 2108	Case 27-2018: A 3-Year-Old Boy with Seizures.<br>Gorman MP, Gombolay GY, Mehan WA Jr, Thibert RL.<br>N Engl J Med. 2018 Aug 30;379(9):870-878.
Gough - Nilforooshan, 2016	Electroconvulsive therapy and/or plasmapheresis in autoimmune encephalitis?<br>Gough JL, Coebergh J, Chandra B, Nilforooshan R.<br>World J Clin Cases. 2016 Aug 16;4(8):223-8.
Graus, Saiz - Dalmau - 2008	Neuronal surface antigen antibodies in limbic encephalitis: clinical-immunologic associations.<br>Graus F, Saiz A, Lai M, Bruna J, López F, Sabater L, Blanco Y, Rey MJ, Ribalta T, Dalmau J.<br>Neurology. 2008 Sep 16;71(12):930-6.
Greiner -Krueger, 2011	Anti-NMDA receptor encephalitis presenting with imaging findings and clinical features mimicking Rasmussen syndrome.<br>Greiner H, Leach JL, Lee KH, Krueger DA.<br>Seizure. 2011 Apr;20(3):266-70.
Grewal - Tripathi, 2018	Confusional state in a pregnant woman: A case of NMDA receptor encephalitis during pregnancy.<br>Grewal KS, Bhatia R, Singh N, Singh R, Dash D, Tripathi M.<br>J Neuroimmunol. 2018 Oct 19;325:29-31.
Guan - Jia, 2015	Non-tumor-Associated Anti-N-Methyl-D-Aspartate (NMDA) Receptor Encephalitis in Chinese Girls With Positive Anti-thyroid Antibodies.<br>Guan W, Fu Z, Zhang H, Jing L, Lu J, Zhang J, Lu H, Teng J, Jia Y.<br>J Child Neurol. 2015 Oct;30(12):1582-5.
Gulyayeva - Duhamel, 2014	Anti-NMDA receptor encephalitis: psychiatric presentation and diagnostic challenges from psychosomatic medicine perspective.<br>Gulyayeva NA, Massie MJ, Duhamel KN.<br>Palliat Support Care. 2014 Apr;12(2):159-63.
Guo - Lin, 2014	Rehabilitation for a child with recalcitrant anti-N-methyl-d-aspartate receptor encephalitis: case report and literature review.<br>Guo YH, Kuan TS, Hsieh PC, Lien WC, Chang CK, Lin YC.<br>Neuropsychiatr Dis Treat. 2014 Nov 24;10:2263-7. doi: 10.2147/NDT.S74205. eCollection 2014.
Gurcharran - Karkare, 2017	Anti-N-Methyl-D-Aspartate Receptor Encephalitis and Rasmussen-like Syndrome: An Association?<br>Gurcharran K, Karkare S.<br>Pediatr Neurol. 2017 Jan;66:104-107.
Haberlandt -Rostásy, 2017	Epileptic phenotypes, electroclinical features and clinical characteristics in 17 children with anti-NMDAR encephalitis.<br>Haberlandt E, Ensslen M, Gruber-Sedlmayr U, Plecko B, Brunner-Krainz M, Schimmel M, Schubert-Bast S, Neirich U, Philippi H, Kurleman G, Tardieu M, Wohlrab G, Borggraefe I, Rostásy K.<br>Eur J Paediatr Neurol. 2017 May;21(3):457-464.
Hachiya – Sakuma, 2013	Rituximab ameliorates anti-N-methyl-D-aspartate receptor encephalitis by removal of short-lived plasmablasts.<br>Hachiya Y, Uruha A, Kasai-Yoshida E, Shimoda K, Satoh-Shirai I, Kumada S, Kurihara E, Suzuki K, Ohba A, Hamano S, Sakuma H.<br>J Neuroimmunol. 2013 Dec 15;265(1-2):128-30
Hacohen - Lin, 2016	N-methyl-d-aspartate (NMDA) receptor antibodies encephalitis mimicking an autistic regression.<br>Hacohen Y, Wright S, Gadian J, Vincent A, Lim M, Wassmer E, Lin JP.<br>Dev Med Child Neurol. 2016 Oct;58(10):1092-4.
Hacohen - Vincent, 2015	Clinical relevance of voltage-gated potassium channel–complex antibodies in children.<br>Hacohen Y, Singh R, Rossi M, Lang B, Hemingway C, Lim M, Vincent A.<br>Neurology. 2015 Sep 15;85(11):967-75.
Hacohen, Absoud - Lim, 2014	NMDA receptor antibodies associated with distinct white matter syndromes.<br>Hacohen Y, Absoud M, Hemingway C, Jacobson L, Lin JP, Pike M, Pullaperuma S, Siddiqui A, Wassmer E,

© 2021 Nosadini M et al. *JAMA Neurology*.

	Waters P, Irani SR, Buckley C, Vincent A, Lim M.<br>Neurol Neuroimmunol Neuroinflamm. 2014 Apr 24;1(1):e2.
Hacohen, Dlamini - Lim, 2014	N-methyl-D-aspartate receptor antibody-associated movement disorder without encephalopathy.<br>Hacohen Y, Dlamini N, Hedderly T, Hughes E, Woods M, Vincent A, Lim M.<br>Dev Med Child Neurol. 2014 Feb;56(2):190-3.
Halbert, 2016	Anti-N-Methyl-D-Aspartate Receptor Encephalitis: A Case Study.<br>Halbert RK.<br>J Neurosci Nurs. 2016 Oct;48(5):270-3.
Hallowell - Hand, 2017	Rituximab for Treatment of Refractory Anti-NMDA Receptor Encephalitis in a Pediatric Patient.<br>Hallowell S, Tebedge E, Oates M, Hand E.<br>J Pediatr Pharmacol Ther. 2017 Mar-Apr;22(2):118-123.
Han - Kapoor, 2017	Chronic Neuropsychological Sequelae in a Patient with Nontumorous Anti-NMDA-Receptor Encephalitis.<br>Han DY, Koehl LM, Patel A, Zhou Z, Phillips S, Kapoor S.<br>Case Rep Neurol Med. 2017;2017:5675732.
Haneche – Pourcher, 2018	An anti-NMDA receptor encephalitis mimicking an HIV encephalitis.<br>Haneche F, Demeret S, Psimaras D, Katlama C, Pourcher V.<br>Clin Immunol. 2018 May 14;193:10-11.
Hansen – Wandinger, 2013	Persistent intrathecal antibody synthesis 15 years after recovering from anti-N-methyl-D-aspartate receptor encephalitis.<br>Hansen HC, Klingbeil C, Dalmau J, Li W, Weissbrich B, Wandinger KP.<br>JAMA Neurol. 2013 Jan;70(1):117-9.
Hara – Dalmau, 2011	Anti-N-methyl-D-aspartate receptor encephalitis associated with carcinosarcoma with neuroendocrine differentiation of the uterus.<br>Hara M, Morita A, Kamei S, Yamaguchi M, Homma T, Nemoto N, Sugita K, Yamamoto T, Dalmau J.<br>J Neurol. 2011 Jul;258(7):1351-3.
Hatanaka - Tamai, 2018	The efficacy of adrenocorticotropic hormone in a girl with anti-N-methyl-D-aspartate receptor encephalitis.<br>Hatanaka M, Shimakawa S, Okumura A, Natsume J, Fukui M, Nomura S, Kashiwagi M, Tamai H.<br>Brain Dev. 2018 Mar;40(3):247-250.
Hattori - Matsukawa, 2017	Anti-N-methyl-d-aspartate receptor limbic encephalitis associated with mature cystic teratoma of the fallopian tube.<br>Hattori Y, Yamashita Y, Mizuno M, Katano K, Sugiura-Ogasawara M, Matsukawa N.<br>J Obstet Gynaecol Res. 2017 Feb;43(2):412-415.
Hau - Hollody, 2016	Anti-N-methyl-D-aspartate receptor encephalitis and drug abuse - the probable role of molecular mimicry or the overstimulation of CB receptors in a 17-year-old adolescent - case report.<br>Hau L, Csabi G, Rozsai B, Stankovics J, Tenyi T, Hollody K.<br>Neuropsychopharmacol Hung. 2016 Sep;18(3):162-164.
Hayashi - Fukasawa, 2014	Successful Laparoscopic Resection of 7 mm Ovarian Mature Cystic Teratoma Associated with Anti-NMDAR Encephalitis.<br>Hayashi M, Motegi E, Honma K, Masawa N, Sakuta H, Hirata K, Kaji Y, Fukasawa I.<br>Case Rep Obstet Gynecol. 2014;2014:618742.
Hébert - Tang-Wai, 2018	Adult-Onset Anti-N-methyl-D-aspartate-receptor Encephalitis Presenting as a Non-Fluent Aphasia.<br>Hébert J, El-Sadi F, Maurice C, Wennberg RA, Tang-Wai DF.<br>Can J Neurol Sci. 2018 Mar;45(2):248-251.
Heekin -Catalano, 2015	Anti-NMDA Receptor Encephalitis in a Patient with Previous Psychosis and Neurological Abnormalities: A Diagnostic Challenge.<br>Heekin RD, Catalano MC, Frontera AT, Catalano G.<br>Case Rep Psychiatry. 2015;2015:253891
Hegarty – Mikli, 2013	Behavioural disturbance requiring medical referral: A case of anti-N-methyl-D-aspartate receptor encephalitis in the emergency department.<br>Hegarty CP, Mikli JE.<br>Emerg Med Australas. 2013 Feb;25(1):87-9.
Hegen -Schmutzhard, 2016	Bi-insular cortical involvement in anti-NMDA-receptor encephalitis - a case report.<br>Hegen H, Uprimny C, Grams A, Virgolini I, Ramberger M, Beer R, Helbok R, Pfausler B, Schmutzhard E.<br>BMC Neurol. 2016 Aug 8;16:130.
Heine - Harms, 2016	Immunoadsorption or plasma exchange in the treatment of autoimmune encephalitis: a pilot study.<br>Heine J, Ly LT, Lieker I, Slowinski T, Finke C, Prüss H, Harms L.<br>J Neurol. 2016 Dec;263(12):2395-2402.
Henry - de Broucker, 2009	[Autoimmune limbic encephalitis with anti-NMDA receptor antibodies and ovarian teratoma: a treatable form of paraneoplastic limbic encephalitis].<br>Henry C, Husson H, de Broucker T.<br>Rev Neurol (Paris). 2009 Jan;165(1):70-5.
Heresco-Levy - Mori, 2015	Clinical and electrophysiological effects of D-serine in a schizophrenia patient positive for anti-N-methyl-D-aspartate receptor antibodies.<br>Heresco-Levy U, Durrant AR, Ermilov M, Javitt DC, Miya K, Mori H. Biol Psychiatry. 2015 Mar 15;77(6):e27-9.
Hermans - Lemmens, 2018	Anti-NMDA receptor encephalitis: still unknown and underdiagnosed by physicians and especially by psychiatrists?<br>Hermans T, Santens P, Matton C, Oostra K, Heylens G, Herremans S, Lemmens GMD.<br>Acta Clin Belg. 2018 Oct;73(5):364-367.
Hernaez-Goni - Tirapu-Ustarroz, 2017	[A new case of psychotic break as the presenting symptom of anti-NMDA receptor encephalitis. Treatment from a neuropsychiatric approach].

© 2021 Nosadini M et al. *JAMA Neurology*.

	Hernaez-Goni P, Luna-Lario P, Tirapu-Ustarroz J.<br>Rev Neurol. 2017 Jul 1;65(1):26-30.
Herrero-Velázquez - Dalmau-Obrador, 2010	[Encephalitis due to antibodies against the NMDA receptor. A case report of a female patient with no associated tumour and a literature review].<br>Herrero-Velázquez S, Luis Guerrero-Peral A, Gámez-Leyva G, Fernández-Buey MN, Conde A, Rodríguez M, Rojo-Martínez E, Pascual J, Fernández-Herranz MR, Dalmau-Obrador J.<br>Rev Neurol. 2010 Jun 1;50(11):661-6. Spanish.
Hilderink - Bunt, 2015	Transient anti-NMDAR encephalitis in a newborn infant due to transplacental transmission.<br>Hilderink M, Titulaer MJ, Schreurs MW, Keizer K, Bunt JE.<br>Neurol Neuroimmunol Neuroinflamm. 2015 Jun 18;2(4):e126.
Hinkle - Heffelfinger, 2016	Neuropsychological characterization of three adolescent females with anti-NMDA receptor encephalitis in the acute, post-acute, and chronic phases: an inter-institutional case series.<br>Hinkle CD, Porter JN, Waldron EJ, Klein H, Tranel D, Heffelfinger A.<br>Clin Neuropsychol. 2017 Jan;31(1):268-288.
Hinson – Bourdette, 2013	Anti-NMDA receptor encephalitis with paroxysmal sympathetic hyperactivity: an under-recognized association?<br>Hinson HE, Takahashi C, Altowaijri G, Baguley IJ, Bourdette D.<br>Clin Auton Res. 2013 Apr;23(2):109-11
Ho, Chan - Wong , 2018	Anti-N-methyl-d-aspartate receptor encephalitis in children: Incidence and experience in Hong Kong.<br>Ho AC, Chan SH, Chan E, Wong SS, Fung ST, Cherk SW, Fung EL, Ma KH, Tsui KW, Yau EK, Wong VC.<br>Brain Dev. 2018 Jun;40(6):473-479.
Hofmann – Schroten, 2011	Anti-NMDA receptor encephalitis after TdaP-IPV booster vaccination: cause or coincidence?<br>Hofmann C, Baur MO, Schroten H.<br>J Neurol. 2011 Mar;258(3):500-1.
Hole – Sokol, 2014	NMDA receptor encephalitis: late treatment also effective.<br>Hole MK, Lennon VA, Cohen ML, Sokol DK.<br>Pediatr Neurol. 2014 Jan;50(1):115-6.
Hollódy - Illés, 2011	[Anti-NMDA-receptor encephalitis: description of the syndrome in connection with the first Hungarian patient].<br>Hollódy K, Csábi G, Láng A, Rózsai B, Komáromy H, Bors L, Illés Z.<br>Ideggyogy Sz. 2011 Mar 30;64(3-4):119-25. Hungarian.
Holzer - Seeck, 2012	Antibody-mediated status epilepticus: a retrospective multicenter survey.<br>Holzer FJ, Rossetti AO, Heritier-Barras AC, Zumsteg D, Roebling R, Huber R, Lerche H, Kiphuth IC, Bardutzky J, Bien CG, Tröger M, Schoch G, Prüss H, Seeck M.<br>Eur Neurol. 2012;68(5):310-7
Hopkins - Chan, 2013	Autoimmune limbic encephalitis presenting as relapsing psychosis.<br>Hopkins SA, Moodley KK, Chan D.<br>BMJ Case Rep. 2013 Aug 30;2013.
Houtrow - Neufeld, 2012	The rehabilitation of children with anti-N-methyl-D-aspartate-receptor encephalitis: a case series.<br>Houtrow AJ, Bhandal M, Pratini NR, Davidson L, Neufeld JA.<br>Am J Phys Med Rehabil. 2012 May;91(5):435-41.
Howard - Guntupalli, 2014	Challenges in providing critical care for patients with anti-N-methyl-D-aspartate receptor encephalitis.<br>Howard CM, Kass JS, Bandi VDP, Guntupalli KK.<br>Chest. 2014 May;145(5):1143-1147.
Hsu - Chang, 2014	Paraneoplastic neurological disorders in children with benign ovarian tumors.<br>Hsu MH, Huang CC, Hung PL, Huang HM, Huang LT, Huang CC, Sheen JM, Huang SC, Chang YC.<br>Brain Dev. 2014 Mar;36(3):248-53.
Hsu - Yang, 2017	Anti-N-Methyl-D-Aspartate-Receptor Encephalitis Complicated With Antiphospholipid Syndrome and Cerebral Venous Thrombosis.<br>Hsu YW, Juan CJ, Lee JT, Lin YK, Lai CH, Yang FC.<br>J Clin Rheumatol. 2017 Aug;23(5):294-295.
Huang - Guo, 2015	Anti-N-methyl-d-aspartate receptor encephalitis in a patient with a 7-year history of being diagnosed as schizophrenia: complexities in diagnosis and treatment.<br>Huang C, Kang Y, Zhang B, Li B, Qiu C, Liu S, Ren H, Yang Y, Liu X, Li T, Guo W.<br>Neuropsychiatr Dis Treat. 2015 Jun 11;11:1437-42.
Hung – Lai, 2011	Anti-N-methyl-d-aspartate receptor encephalitis.<br>Hung TY, Foo NH, Lai MC.<br>Pediatr Neonatol. 2011 Dec;52(6):361-4.
Hur, 2015	Fever of Unknown Origin: An Unusual Presentation of Anti-N-Methyl-D-Aspartate Receptor Encephalitis.<br>Hur J.<br>Infect Chemother. 2015 Jun;47(2):129-32.
Iadisernia – Biancheri, 2012	Anti-N-methyl-D-aspartate-receptor encephalitis: cognitive profile in two children.<br>Iadisernia E, Battaglia FM, Vanadia E, Trapolino E, Vincent A, Biancheri R.<br>Eur J Paediatr Neurol. 2012 Jan;16(1):79-82.
Iemura - Haga, 2018	Histopathological characterization of the neuroglial tissue in ovarian teratoma associated with anti-N-methyl-D-aspartate (NMDA) receptor encephalitis.<br>Iemura Y, Yamada Y, Hirata M, Kataoka TR, Minamiguchi S, Haga H.<br>Pathol Int. 2018 Nov 14. doi: 10.1111/pin.12732. [Epub ahead of print]
Iglesias-Alonso - Iglesias-García, 2017	Case Report: Anti-N-methyl-D-aspartate receptor encephalitis with psychiatric symptoms.<br>Iglesias-Alonso A, Iglesias-García C.<br>Actas Esp Psiquiatr. 2017 Jan;45(1):39-46.

© 2021 Nosadini M et al. *JAMA Neurology*.

Iizuka - Nishiyama, 2016	Association of Progressive Cerebellar Atrophy With Long-term Outcome in Patients With Anti-N-Methyl-d-Aspartate Receptor Encephalitis.<br>Iizuka T, Kaneko J, Tominaga N, Someko H, Nakamura M, Ishima D, Kitamura E, Masuda R, Oguni E, Yanagisawa T, Kanazawa N, Dalmau J, Nishiyama K.<br>JAMA Neurol. 2016 Jun 1;73(6):706-13.
Iizuka - Dalmau, 2008	Anti-NMDA receptor encephalitis in Japan: long-term outcome without tumor removal.<br>Iizuka T, Sakai F, Ide T, Monzen T, Yoshii S, Iigaya M, Suzuki K, Lynch DR, Suzuki N, Hata T, Dalmau J.<br>Neurology. 2008 Feb 12;70(7):504-11. Epub 2007 Sep 26.
Ikeguchi – Yamamoto, 2012	Rituximab used successfully in the treatment of anti-NMDA receptor encephalitis.<br>Ikeguchi R, Shibuya K, Akiyama S, Hino S, Kubo H, Takeda T, Shibata N, Yamamoto K.<br>Intern Med. 2012;51(12):1585-9. Epub 2012 Jun 15.
Imai - Sumi, 2015	Complete recovery from paraneoplastic anti-NMDAR encephalitis associated with a small ovarian teratoma following a laparoscopic salpingo-oophorectomy: A case report.<br>Imai K, Fukuda T, Wada T, Kawanishi M, Yamauchi M, Hashiguchi Y, Ichimura T, Yasui T, Sumi T.<br>Exp Ther Med. 2015 May;9(5):1723-1726.
Inoue - Suzuki, 2018	A Case of Paraneoplastic Limbic Encephalitis in a Patient with Invasive Thymoma with Anti-Glutamate Receptor Antibody-Positive Cerebrospinal Fluid: A Case Report.<br>Inoue T, Kanno R, Moriya A, Nakamura K, Watanabe Y, Matsumura Y, Suzuki H.<br>Ann Thorac Cardiovasc Surg. 2018 Aug 20;24(4):200-204.
Ioannidis - Karacostas, 2015	Anti-NMDA receptor encephalitis possibly triggered by measles virus.<br>Ioannidis P, Papadopoulos G, Koufou E, Parissis D, Karacostas D.<br>Acta Neurol Belg. 2015 Dec;115(4):801-2.
Irani, Bera - Vincent, 2010	N-methyl-D-aspartate antibody encephalitis: temporal progression of clinical and paraclinical observations in a predominantly non-paraneoplastic disorder of both sexes.<br>Irani SR, Bera K, Waters P, Zuliani L, Maxwell S, Zandi MS, Friese MA, Galea I, Kullmann DM, Beeson D, Lang B, Bien CG, Vincent A.<br>Brain. 2010 Jun;133(Pt 6):1655-67.
Iriondo - Aguilera, 2017	Anti-NMDA (a-NMDAR) receptor encephalitis related to acute consumption of metamphetamine: Relevance of differential diagnosis.<br>Iriondo O, Zaldibar-Gerrikagoitia J, Rodríguez T, García JM, Aguilera L.<br>Rev Esp Anestesiol Reanim. 2017 Mar;64(3):172-176.
Ishikawa - Iwasaki, 2013	Ophthalmoplegia and flaccid paraplegia in a patient with anti-NMDA receptor encephalitis: a case report and literature review.<br>Ishikawa Y, Ikeda K, Murata K, Hirayama T, Takazawa T, Yanagihashi M, Kano O, Kawabe K, Takahashi Y, Iwasaki Y.<br>Intern Med. 2013;52(24):2811-5. Review.
Ishiura – Tsuji, 2008	Response of anti-NMDA receptor encephalitis without tumor to immunotherapy including rituximab.<br>Ishiura H, Matsuda S, Higashihara M, Hasegawa M, Hida A, Hanajima R, Yamamoto T, Shimizu J, Dalmau J, Tsuji S.<br>Neurology. 2008 Dec 2;71(23):1921-3.
Ito – Araki, 2010	[Anti-NMDA receptor encephalitis during pregnancy].<br>Ito Y, Abe T, Tomioka R, Komori T, Araki N.<br>Rinsho Shinkeigaku. 2010 Feb;50(2):103-7. Japanese.
Iwamoto - Nakao, 2014	Improvement of NMDA encephalitis by active lymph node removal.<br>Iwamoto T, Shiokawa Y, Nakao S.<br>J Anesth. 2014 Aug;28(4):646.
Jagota - Bhidayasiri, 2014	Transplacental transfer of NMDA receptor antibodies in an infant with cortical dysplasia.<br>Jagota P, Vincent A, Bhidayasiri R.<br>Neurology. 2014 May 6;82(18):1662-3.
Jain - Go, 2018	Extreme delta brushes in a 14-year old girl with anti-NMDAR encephalitis.<br>Jain P, Whitney R, Go C.<br>Neurol India. 2018 Mar-Apr;66(2):536-538.
Jandu - Vidgeon, 2016	Status epilepticus and anti-NMDA receptor encephalitis after resection of an ovarian teratoma.<br>Jandu AS, Odor PM, Vidgeon SD.<br>J Intensive Care Soc. 2016 Nov;17(4):346-352.
Janmohamed - Salman, 2018	Primary lateral sclerosis-like picture in a patient with a remote history of anti-N-methyl-D- aspartate receptor (anti-NMDAR) antibody encephalitis.<br>Janmohamed M, Knezevic W, Needham M, Salman S.<br>BMJ Case Rep. 2018 Jun 10;2018.
Jensen - Pinborg , 2015	Anti-NMDAR encephalitis: demonstration of neuroinflammation and the effect of immunotherapy.<br>Jensen P, Kondziella D, Thomsen G, Dyssegaard A, Svarer C, Pinborg LH.<br>Neurology. 2015 Feb 24;84(8):859.
Jeraiby - Paul, 2016	A case of anti-NMDA receptor encephalitis in a woman with a NMDA-R(+) small cell lung carcinoma (SCLC).<br>Jeraiby M, Depincé-Berger A, Bossy V, Antoine JC, Paul S.<br>Clin Immunol. 2016 May;166-167:96-9.
Jesse - Lewerenz, 2018	On Razor's edge: Managing analgosedation during severe anti-NMDA receptor encephalitis.<br>Jesse S, Wagner J, Gastl R, Steinacker P, Otto M, Kassubek J, Lewerenz J.<br>Neurol Neuroimmunol Neuroinflamm. 2018 Nov 19;6(1):e522.

© 2021 Nosadini M et al. *JAMA Neurology*.

Jinte - Guy, 2018	EBV-NMDA double positive encephalitis in an immunocompromised patient.<br>Jinte G, Mathieu S, Deborah VM, Guy L.<br>J Neurol Sci. 2018 Nov 3;396:76-77.
Joe - Desai, 2016	An Atypical Case of Anti-N-Methyl-D-Aspartate Receptor Encephalitis.<br>Joe E, Desai J.<br>Pediatr Neurol. 2016 Oct;63:80-81.
Johnson - Dalmau, 2010	Anti-NMDA receptor encephalitis causing prolonged nonconvulsive status epilepticus.<br>Johnson N, Henry C, Fessler AJ, Dalmau J.<br>Neurology. 2010 Oct 19;75(16):1480-2.
Jones - Kahn, 2015	A Case of Anti-NMDA Receptor Encephalitis Treated with ECT.<br>Jones KC, Schwartz AC, Hermida AP, Kahn DA.<br>J Psychiatr Pract. 2015 Sep;21(5):374-80.
Jones - Lockwood, 2014	A Case of Anti-NMDA Receptor Encephalitis With the Highest Reported CSF White Cells to Date.<br>Jones SV, Breakey RW, Lockwood BM.<br>J Neuropsychiatry Clin Neurosci. 2014 Fall;26(4):E10-1.
Jones - Sharpe, 2017	Anti-N-methyl-d-aspartate receptor encephalitis in Māori and Pacific Island children in New Zealand.<br>Jones HF, Mohammad SS, Reed PW, Dunn PPJ, Steele RH, Dale RC, Sharpe C.<br>Dev Med Child Neurol. 2017 Jul;59(7):719-724.
Jones - Yazbek, 2017	Ultrasound-guided laparoscopic ovarian preserving surgery to treat anti-NMDA receptor encephalitis.<br>Jones BP, Rees R, Saso S, Stalder C, Smith JR, Yazbek J.<br>BJOG. 2017 Jan;124(2):337-341.
Joseph - Baltimore, 2015	A 14-Year-Old Girl with Slurred Speech, Aggressive Behavior, and Seizures.<br>Joseph K, Oliveira CR, Baltimore RS.<br>Pediatr Ann. 2015 Jun;44(6):236-7.
Jun , Seo - Lee, 2017	Botulinum toxin treatment for hypersalivation in anti-NMDA receptor encephalitis.<br>Jun JS, Seo HG, Lee ST, Chu K, Lee SK.<br>Ann Clin Transl Neurol. 2017 Sep 26;4(11):830-834.
Kadoya - Kaida, 2015	An Atypical Case of Anti-NMDA Receptor Encephalitis: Predominant Parkinsonism and Persisting Micrographia without Oro-facial Dyskinesia.<br>Kadoya M, Kadoya A, Onoue H, Ikewaki K, Kaida K.<br>Intern Med. 2015;54(15):1927-32.
Kadoya -Kaida, 2018	Refractory status epilepticus caused by anti-NMDA receptor encephalitis that markedly improved following combination therapy with rituximab and cyclophosphamide.<br>Kadoya M, Onoue H, Kadoya A, Ikewaki K, Kaida K.<br>Intern Med. 2015;54(2):209-13.
Kalam - Singh-Curry, 2018	Anti-NMDAR encephalitis complicating pregnancy.<br>Kalam S, Baheerathan A, McNamara C, Singh-Curry V.<br>Pract Neurol. 2018 Oct 10. pii: practneurol-2018-002042.
Kaplan -Probasco, 2017	Limbic and new onset refractory tonic status epilepticus (NORSE) in anti-NMDAR encephalitis.<br>Kaplan PW, Probasco J.<br>Clin Neurophysiol Pract. 2017 Jul 1;2:140-143. doi: 10.1016/j.cnp.2017.06.003.
Kashyape - Whitney, 2012	Successful treatment of two paediatric cases of anti-NMDA receptor encephalitis with cyclophosphamide: the need for early aggressive immunotherapy in tumour negative paediatric patients.<br>Kashyape P, Taylor E, Ng J, Krishnakumar D, Kirkham F, Whitney A.<br>Eur J Paediatr Neurol. 2012 Jan;16(1):74-8.
Kataoka – Ueno, 2012	Low-voltage EEG activity presenting from psychotic stage in a patient with anti-NMDA receptor encephalitis.<br>Kataoka H, Takatani T, Ueno S.<br>BMJ Case Rep. 2012 Oct 30;2012.
Kataoka - Ueno, 2017	Early progression of brain atrophy in patients with anti-N-methyl-D-aspartate receptor encephalitis: Case reports.<br>Kataoka H, Sawa N, Tonomura Y, Ueno S.<br>Medicine (Baltimore). 2017 Apr;96(17):e6776.
Kattepur – Gopinath, 2014	Anti-NMDAR limbic encephalitis--a clinical curiosity.<br>Kattepur AK, Patil D, Shankarappa A, Swamy S, Chandrashekar NS, Chandrashekar P, Prabhu S, Gopinath KS.<br>World J Surg Oncol. 2014 Aug 9;12:256.
Kaur - Jain, 2014	Anti-N-methyl-D-aspartate receptor encephalitis: A case report and review of the literature.<br>Kaur S, Juneja M, Mishra D, Jain S.<br>J Pediatr Neurosci. 2014 May;9(2):145-7
Kawano – Oshita, 2011	Anaesthesia for a patient with paraneoplastic limbic encephalitis with ovarian teratoma: relationship to anti-N-methyl-D-aspartate receptor antibodies.<br>Kawano H, Hamaguchi E, Kawahito S, Tsutsumi YM, Tanaka K, Kitahata H, Oshita S.<br>Anaesthesia. 2011 Jun;66(6):515-8.
Kayal - Synmon, 2014	Relapsing Anti-NMDAR Encephalitis after a gap of eight years in a girl from North-East India.<br>Kayal AK, Das M, Bhowmick S, Synmon B.<br>Ann Indian Acad Neurol. 2014 Jul;17(3):349-51.
Kayser - Dalmau, 2013	Frequency and characteristics of isolated psychiatric episodes in anti–N-methyl-d-aspartate receptor encephalitis.<br>Kayser MS, Titulaer MJ, Gresa-Arribas N, Dalmau J.<br>JAMA Neurol. 2013 Sep 1;70(9):1133-9.
Keddie - Lunn, 2018	Plasma cell depletion with bortezomib in the treatment of refractory NMDAR-antibody encephalitis. Rational developments in neuroimmunological treatment.

© 2021 Nosadini M et al. *JAMA Neurology*.

	Keddie S, Crisp SJ, Blackaby J, Cox A, Coles A, Hart M, Church AJ, Vincent A, Zandi M, Lunn MP.<br>Eur J Neurol. 2018 Jul 23.
Keller - Lotan, 2014	Anti-NMDA Receptor Encephalitis Presenting as an Acute Psychotic Episode in a Young Woman: An Underdiagnosed yet Treatable Disorder.<br>Keller S, Roitman P, Ben-Hur T, Bonne O, Lotan A.<br>Case Rep Psychiatry. 2014;2014:868325.
Khadem – White, 2009	Anti-N-methyl-D-aspartate receptor antibody limbic encephalitis.<br>Khadem GM, Heble S, Kumar R, White C.<br>Intern Med J. 2009 Jan;39(1):54-6.
Khoo - Rahman, 2017	An unusual case of refractory status epilepticus in a young lady: anti-NMDA receptor encephalitis.<br>Khoo CS, Zulkifli NH, Rahman SSA.<br>Clin Med (Lond). 2017 Oct;17(5):436-438.
Kiani - Gumber, 2015	Anti-NMDA-receptor encephalitis presenting with catatonia and neuroleptic malignant syndrome in patients with intellectual disability and autism.<br>Kiani R, Lawden M, Eames P, Critchley P, Bhaumik S, Odedra S, Gumber R.<br>BJPsych Bull. 2015 Feb;39(1):32-5.
Kim - Jung, 2015	Anti-NMDA Receptor Encephalitis in a Pregnant Woman.<br>Kim J, Park SH, Jung YR, Park SW, Jung DS.<br>J Epilepsy Res. 2015 Jun 30;5(1):29-32.
Kim - Kang, 2015	Anti-NMDA Receptor Antibody Encephalitis Presenting with Unilateral Non-convulsive Status Epilepticus in a Male Patient.<br>Kim H, Ryu H, Kang JK.<br>J Epilepsy Res. 2015 Jun 30;5(1):17-9.
Kim - Lee, 2014	Screening Autoimmune Anti-neuronal Antibodies in Pediatric Patients with Suspected Autoimmune Encephalitis.<br>Kim SY, Choi SA, Ryu HW, Kim H, Lim BC, Hwang H, Chae JH, Choi J, Kim KJ, Hwang YS, Lee ST, Chu K, Lee SK.<br>J Epilepsy Res. 2014 Dec 31;4(2):55-61.
Kim, Kang - Day, 2018	Autoimmune Encephalitis With Multiple Autoantibodies: A Diagnostic and Therapeutic Challenge.<br>Kim AE, Kang P, Bucelli RC, Ferguson CJ, Schmidt RE, Varadhachary AS, Day GS.<br>Neurologist. 2018 Mar;23(2):55-59.
Kim, Kim - Lee, 2016	A young child of anti-NMDA receptor encephalitis presenting with epilepsia partialis continua: the first pediatric case in Korea.<br>Kim EH, Kim YJ, Ko TS, Yum MS, Lee JH.<br>Korean J Pediatr. 2016 Nov;59(Suppl 1):S133-S138.
Kim, Park - Lee, 2018	Anti-NMDAR Encephalitis in a 13-Year-Old Female: A 24-Month Clinical Follow-Up.<br>Kim E, Park EG, Lee J, Lee M, Kim J, Lee J.<br>J Epilepsy Res. 2018 Jun 30;8(1):41-48.
Kinno – Kinugasa, 2013	Cerebellar symptoms in a case of acute limbic encephalitis associated with autoantibodies to glutamate receptors δ2 and ε2.<br>Kinno R, Yamazaki T, Yamamoto M, Takahashi Y, Fukui T, Kinugasa E.<br>Clin Neurol Neurosurg. 2013 Apr;115(4):481-3
Kirkpatrick - Abou-Khalil, 2011	Rhythmic delta activity represents a form of nonconvulsive status epilepticus in anti-NMDA receptor antibody encephalitis.<br>Kirkpatrick MP, Clarke CD, Sonmezturk HH, Abou-Khalil B.<br>Epilepsy Behav. 2011 Feb;20(2):392-4.
Kitada Kusunoki, 2011	[Dramatic improvement in two cases of anti-NMDA receptor encephalitis after immunomodulating therapy].<br>Kitada M, Suzuki H, Ichihashi J, Mitsui Y, Tanaka K, Kusunoki S.<br>Rinsho Shinkeigaku. 2011 Sep;51(9):683-7. Japanese.
Kleinig - Blumbergs, 2008	The distinctive movement disorder of ovarian teratoma-associated encephalitis.<br>Kleinig TJ, Thompson PD, Matar W, Duggins A, Kimber TE, Morris JG, Kneebone CS, Blumbergs PC.<br>Mov Disord. 2008 Jul 15;23(9):1256-61.
Kleyensteuber - Loeffler, 2010	Limbic encephalitis presenting with seizures, anterograde amnesia, and psychosis in a patient seven weeks status post immature ovarian teratoma removal.<br>Kleyensteuber B, Ruterbusch V, Bennett J, Llewellyn D, Loeffler G.<br>Mil Med. 2010 Aug;175(8):616-8.
Kobayashi - Hattor, 2017	Anti-NMDA receptor encephalitis due to large-cell neuroendocrine carcinoma of the uterus.<br>Kobayashi M, Nishioka K, Takanashi M, Hattori A, Shojima Y, Hayashida A, Sumii A, Ota T, Terao Y, Yokoyama K, Hattori N.<br>J Neurol Sci. 2017 Dec 15;383:72-74.
Kobayashi -Takamatsu, 2018	Anti-N-methyl-D-aspartate receptor encephalitis relapse in the brainstem.<br>Kobayashi Y, Sato S, Takasone K, Takamatsu R.<br>BMJ Case Rep. 2018 Apr 7;2018. pii: bcr-2018-224584.
Kohler - Fassbender, 2015	Tryptophan immunoadsorption for the treatment of autoimmune encephalitis.<br>Köhler W, Ehrlich S, Dohmen C, Haubitz M, Hoffmann F, Schmidt S, Klingel R, Kraft A, Neumann-Haefelin T, Topka H, Stich O, Baumgartner A, Fassbender C.<br>Eur J Neurol. 2015 Jan;22(1):203-6.
Koksal - Keskek, 2015	A case of NMDAR encephalitis misdiagnosed as postpartum psychosis and neuroleptic malignant syndrome.<br>Koksal A, Baybas S, Mutluay B, Altunkaynak Y, Keskek A.<br>Neurol Sci. 2015 Jul;36(7):1257-8.

© 2021 Nosadini M et al. *JAMA Neurology*.

Kokubun - Hirata, 2016	Pregnancy and delivery in anti-NMDA receptor encephalitis survivors.<br>Kokubun N, Komagamine T, Hirata K.<br>Neurol Clin Pract. 2016 Oct;6(5):e40-e43.
Konuskan - Anlar, 2018	Clinical presentation of anti-N-methyl-d-aspartate receptor and anti-voltage-gated potassium channel complex antibodies in children: A series of 24 cases.<br>Konuskan B, Yildirim M, Topaloglu H, Erol I, Oztoprak U, Tan H, Gocmen R, Anlar B.<br>Eur J Paediatr Neurol. 2018 Jan;22(1):135-142.
Kort – Lobo, 2009	Paraneoplastic anti-N-methyl-d-aspartate-receptor encephalitis from mature cystic teratoma.<br>Kort DH, Vallerie AM, DeMarco EF, Lobo RA.<br>Obstet Gynecol. 2009 Aug;114(2 Pt 1):373-6.
Kramina -Viksna, 2015	Acute psychosis due to non-paraneoplastic anti-NMDA-receptor encephalitis in a teenage girl: Case report.<br>Kramina S, Kevere L, Bezborodovs N, Purvina S, Rozentals G, Strautmanis J, Viksna Z.<br>Psych J. 2015 Dec;4(4):226-30.
Kremm -Lawler, 2016	Poster 326 Paraneoplastic Ovarian Teratoma Anti-NMDA Receptor Encephalitis: A Case Report.<br>Kremm LA, Armstrong Q, Lawler MH.<br>PM R. 2016 Sep;8(9S):S267.
Kubota – Nomura, 2012	[Anti-Ma2, anti-NMDA-receptor and anti-GluRε2 limbic encephalitis with testicular seminoma: short-term memory disturbance].<br>Kubota A, Tajima T, Narukawa S, Yamazato M, Fukaura H, Takahashi Y, Tanaka K, Shimizu J, Nomura K.<br>Rinsho Shinkeigaku. 2012;52(9):666-71. Japanese.
Kumar – Tuck, 2013	Acute neuropsychiatric manifestations of anti-N-methyl-D-aspartate receptor encephalitis.<br>Kumar R, Gunaratne D, Khan S, Crawford K, Cook M, Tuck R.<br>Australas Psychiatry. 2013 Jun;21(3):279-80.
Kumar-Dalmau, 2010	Anti-N-methyl-D-aspartate receptor encephalitis during pregnancy.<br>Kumar MA, Jain A, Dechant VE, Saito T, Rafael T, Aizawa H, Dysart KC, Katayama T, Ito Y, Araki N, Abe T, Balice-Gordon R, Dalmau J.<br>Arch Neurol. 2010 Jul;67(7):884-7.
Kumari - Saini, 2017	Anti-N-Methyl-D-Aspartate-Receptor Encephalitis in Young Females.<br>Kumari K, Sahni N, Kumari V, Saini V.<br>Turk J Anaesthesiol Reanim. 2017 Dec;45(6):377-379.
Kümpfel - Prüss, 2016	Delayed diagnosis of extraovarian teratoma in relapsing anti-NMDA receptor encephalitis.<br>Kümpfel T, Gerdes LA, Heck C, Prüss H.<br>Neurol Neuroimmunol Neuroinflamm. 2016 Jun 16;3(4):e250.
Kung – Kass, 2011	Psychiatric manifestations of anti-NMDA receptor encephalitis in a man without tumor.<br>Kung DH, Qiu C, Kass JS.<br>Psychosomatics. 2011 Jan-Feb;52(1):82-5.
Kuo – Yang, 2012	Anti-NMDA receptor encephalitis with the initial presentation of psychotic mania.<br>Kuo YL, Tsai HF, Lai MC, Lin CH, Yang YK.<br>J Clin Neurosci. 2012 Jun;19(6):896-8
Kuppuswamy - Sola, 2014	Management of psychiatric symptoms in anti-NMDAR encephalitis: a case series, literature review and future directions.<br>Kuppuswamy PS, Takala CR, Sola CL.<br>Gen Hosp Psychiatry. 2014 Jul-Aug;36(4):388-91.
Kurian – Korff, 2012	Anti-NMDA receptor encephalitis: the importance of early diagnosis and aggressive immunotherapy in tumor negative pediatric patients.<br>Kurian M, Fluss J, Korff C.<br>Eur J Paediatr Neurol. 2012 Nov;16(6):764-5.
Kurian - Hovarth, 2010	Opsoclonus-myoclonus syndrome in anti-N-methyl-D-aspartate receptor encephalitis.<br>Kurian M, Lalive PH, Dalmau JO, Horvath J.<br>Arch Neurol. 2010 Jan;67(1):118-21.
Kurita - Mori, 2015	Deterioration of clinical features of a patient with autism spectrum disorder after anti-N-methyl-D-aspartate receptor encephalitis.<br>Kurita D, Wakuda T, Takagai S, Takahashi Y, Iwata Y, Suzuki K, Mori N.<br>Psychiatry Clin Neurosci. 2015 Aug;69(8):507.
Labate – Aguglia, 2009	Anti-NMDA receptor encephalitis: a video case report.<br>Labate A, Irani SR, Vincent A, Gambardella A, Piane EL, Cianci V, Aguglia U.<br>Epileptic Disord. 2009 Sep;11(3):267-9.
Labate – Gambardellam, 2013	Anti-N-methyl-D-aspartate-glutamic-receptor encephalitis presenting as paroxysmal exercise-induced foot weakness.<br>Labate A, Quattrone A, Dalmau J, Gambardella A.<br>Mov Disord. 2013 Jun;28(6):820-2.
Lagarde - Guedj, 2016	Cerebral (18)FluoroDeoxy-Glucose Positron Emission Tomography in paediatric anti N-methyl-D-aspartate receptor encephalitis: A case series.<br>Lagarde S, Lepine A, Caietta E, Pelletier F, Boucraut J, Chabrol B, Milh M, Guedj E.<br>Brain Dev. 2016 May;38(5):461-70.
Lalanne - Foucher, 2015	Melancholia Associated With Severe Cognitive Disorders as the Expression of Late-Onset Postpartum Anti-N-Methyl-d-Aspartic Acid Receptor Limbic Encephalitis.<br>Lalanne L, Jantzi C, Gorse A, Zimmermann MA, Danion JM, Foucher J.<br>J Neuropsychiatry Clin Neurosci. 2015;27(2):e168-9.

© 2021 Nosadini M et al. *JAMA Neurology*.

Lamale-Smith - Scott, 2015	Maternal-fetal transfer of anti-N-methyl-D-aspartate receptor antibodies.<br>Lamale-Smith LM, Moore GS, Guntupalli SR, Scott JB.<br>Obstet Gynecol. 2015 May;125(5):1056-8.
Lapébie - François, 2014	Potential side effect of propofol and sevoflurane for anesthesia of anti-NMDA-R encephalitis.<br>Lapébie FX, Kennel C, Magy L, Projetti F, Honnorat J, Pichon N, Vignon P, François B.<br>BMC Anesthesiol. 2014 Jan 16;14:5.
Lasoff - Wardi, 2016	Anti-N-Methyl-D-Aspartate Receptor Encephalitis, an Underappreciated Disease in the Emergency Department.<br>Lasoff DR, Corbett-Detig J, Sell R, Nolan M, Wardi G.<br>West J Emerg Med. 2016 May;17(3):280-2.
Le Foll – Pelletier, 2010	[Psychiatric symptoms of a paraneoplastic anti-N-methyl-D-aspartate receptor encephalitis: A case report].<br>Le Foll J, Pelletier A.<br>Encephale. 2010 Apr;36(2):166-71.
Le Moigno -Castelnau, 2014	[N-methyl-D-aspartate receptor antibody encephalitis: value of immunomodulatory therapy].<br>Le Moigno L, Ternant D, Paintaud G, Thibault G, Cloarec S, Tardieu M, Lagrue E, Castelnau P.<br>Arch Pediatr. 2014 Jun;21(6):620-3. doi: 10.1016/j.arcped.2014.03.005. Epub 2014 May 13. French.
Lebas - Tardieu, 2010	Expanding spectrum of encephalitis with NMDA receptor antibodies in young children.<br>Lebas A, Husson B, Didelot A, Honnorat J, Tardieu M.<br>J Child Neurol. 2010 Jun;25(6):742-5.
Lebon - Perez, 2012	Anti-N-methyl-D-aspartate (NMDA) receptor encephalitis mimicking a primary psychiatric disorder in an adolescent.<br>Lebon S, Mayor-Dubois C, Popea I, Poloni C, Selvadoray N, Gumy A, Roulet-Perez E.<br>J Child Neurol. 2012 Dec;27(12):1607-10.
Lee – Wu, 2018	Fulminant course in a patient with anti-N-methyl-D-aspartate receptor encephalitis with bilateral ovarian teratomas: A case report and literature review.<br>Lee KW, Liou LM, Wu MN.<br>Medicine (Baltimore). 2018 Apr;97(15):e0339.
Lee – Chan, 2018	Anti-NMDA receptor encephalitis associated with ictal asystole.<br>Lee M, Lawn N, Prentice D, Chan J.<br>J Clin Neurosci. 2011 Dec;18(12):1716-8.
Lee - Kim, 2014	18F-Fluorodeoxyglucose Positron-Emission Tomography Findings with Anti-N-Methyl-D-Aspartate Receptor Encephalitis that Showed Variable Degrees of Catatonia: Three Cases Report.<br>Lee EM, Kang JK, Oh JS, Kim JS, Shin YW, Kim CY.<br>J Epilepsy Res. 2014 Dec 31;4(2):69-73.
Lee - Lu, 2016	Long-term and Strong Immunotherapy to Treat Anti-N-Methyl- D-Aspartate Receptor Encephalitis with Refractory Status Epilepticus.<br>Lee LH, Lu CJ.<br>Acta Neurol Taiwan. 2016 Sep 15;25(3):99-103.
Leel - Bouhadiba, 2018	Ovarian teratoma associated with anti-NMDA (N-methyl D-aspartate) receptor encephalitis.<br>Leel N, Thakkar HS, Drake D, Bouhadiba N.<br>BMJ Case Rep. 2018 Mar 13;2018.
Lekoubou - Honnorat, 2012	Anti-N-methyl-D-aspartate receptor encephalitis with acute disseminated encephalomyelitis-like MRI features.<br>Lekoubou A, Viaccoz A, Didelot A, Anastasi A, Marignier R, Ducray F, Rogemond V, Honnorat J.<br>Eur J Neurol. 2012 Feb;19(2):e16-7.
Lesher – Streck, 2010	Anti-N-methyl-D-aspartate receptor encephalitis associated with an ovarian teratoma in an adolescent female.<br>Lesher AP, Myers TJ, Tecklenburg F, Streck CJ.<br>J Pediatr Surg. 2010 Jul;45(7):1550-3.
Leypoldt – Wandinger, 2013	Recovery from severe frontotemporal dysfunction at 3years after N-methyl-d-aspartic acid (NMDA) receptor antibody encephalitis.<br>Leypoldt F, Gelderblom M, Schöttle D, Hoffmann S, Wandinger KP.<br>J Clin Neurosci. 2013 Apr;20(4):611-3.
Li - Hu, 2018	Analysis and discussion of the rare complication of autoimmune encephalitis: Two case reports.<br>Li R, Jiang L, Li XJ, Hong SQ, Zhong M, Hu Y.<br>Medicine (Baltimore). 2018 Jul;97(27):e11202.
Li - Peng, 2018	Anti-N-methyl-D-aspartate receptor encephalitis: A case report.<br>Li H, Guo YK, Cui YL, Peng T.<br>Medicine (Baltimore). 2018 Dec;97(50):e13625.
Li - Ren, 2015	Negative myoclonus in a child with anti-NMDA receptor encephalitis.<br>Li X, Wu D, Fernández IS, Chen J, Jin P, Zhou Z, Wu Y, Jiao J, Ren L.<br>J Neurol Sci. 2015 Nov 15;358(1-2):532-4.
Li - Zhao, 2015	A case of anti-NMDAR encephalitis induced by ovarian teratoma.<br>Li S, Zhao A.<br>Cell Biochem Biophys. 2015 Mar;71(2):1011-4.
Li, Liu - Liu, 2017	Anti-N-methyl-D-aspartate receptor encephalitis associated with mediastinal teratoma: a rare case report and literature review.<br>Li C, Liu C, Lin F, Liu L.<br>J Thorac Dis. 2017 Dec;9(12):E1118-E1121.
Li, Wang – Wu, 2017	Anti-N-Methyl-d-Aspartate Receptor Encephalitis in a Patient with Alcoholism: A Rare Case Report.<br>Li Y, Wang Q, Liu C, Wu Y.<br>Front Psychiatry. 2017 Aug 3;8:141.

© 2021 Nosadini M et al. *JAMA Neurology*.

Liang, Yang - Yu, 2017	Teratoma-associated anti-NMDAR encephalitis: Two cases report and literature review.<br>Liang Z, Yang S, Sun X, Li B, Li W, Liu Z, Yu G.<br>Medicine (Baltimore). 2017 Dec;96(49):e9177.
Liao - Jiang, 2017	Anesthesia management of cesarean section in parturient with anti-N-methyl-D-aspartate receptor encephalitis: a case report.<br>Liao Z, Jiang X, Ni J.<br>J Anesth. 2017 Apr;31(2):282-285.
Liba - Sediva, 2016	Anti-N-methyl-D-aspartate receptor encephalitis: the clinical course in light of the chemokine and cytokine levels in cerebrospinal fluid.<br>Liba Z, Kayserova J, Elisak M, Marusic P, Nohejlova H, Hanzalova J, Komarek V, Sediva A.<br>J Neuroinflammation. 2016 Mar 3;13(1):55.
Lim – Nugent, 2013	Anti-N-methyl-D-aspartate receptor encephalitis.<br>Lim SY, Panikkath R, Mankongpaisarnrung C, Islam E, Mulkey Z, Nugent K.<br>Am J Med Sci. 2013 Jun;345(6):491-3.
Lim - Yip, 2017	Anti-N-methyl-D-aspartate receptor encephalitis associated with hepatic neuroendocrine carcinoma: A case report.<br>Lim EW, Yip CW.<br>J Clin Neurosci. 2017 Jul;41:70-72.
Lin, Lin - Wang, 2014	Anti-N-methyl-D-aspartate receptor encephalitis in Taiwan--a comparison between children and adults.<br>Lin JJ, Lin KL, Hsia SH, Chou ML, Hung PC, Hsieh MY, Chou IJ, Wang HS; Children with Encephalitis and/or Encephalopathy Related Status Epilepticus and Epilepsy (CHEESE) Study Group.<br>Pediatr Neurol. 2014 Jun;50(6):574-80.
Linnoila - McKeon, 2016	CSF herpes virus and autoantibody profiles in the evaluation of encephalitis.<br>Linnoila JJ, Binnicker MJ, Majed M, Klein CJ, McKeon A.<br>Neurol Neuroimmunol Neuroinflamm. 2016 Jun 1;3(4):e245.
Liu - Han, 2015	Anti-N-methyl-D-aspartate receptor encephalitis associated with an ovarian teratoma: two cases report and anesthesia considerations.<br>Liu H, Jian M, Liang F, Yue H, Han R.<br>BMC Anesthesiol. 2015 Oct 16;15:150.
Liu - Liu, 2015	Anti-NMDAR Encephalitis of 11 Cases in China - Detailed Clinical, Laboratory and Imagiological Description.<br>Liu J, Wang D, Xiong Y, Liu B, Liu M.<br>Eur Neurol. 2015;74(1-2):73-8.
Llorens – Zarranz, 2010	Abnormal multifocal cerebral blood flow on Tc-99m HMPAO SPECT in a patient with anti-NMDA-receptor encephalitis.<br>Llorens V, Gabilondo I, Gómez-Esteban JC, Agundez M, Mendibe M, Bergara JC, Ciordia R, Saiz A, Zarranz JJ.<br>J Neurol. 2010 Sep;257(9):1568-9.
Loughan - Malkin, 2016	Anti-N-Methyl-D-Aspartate Receptor Encephalitis: A Review and Neuropsychological Case Study.<br>Loughan AR, Allen A, Perna R, Malkin MG.<br>Clin Neuropsychol. 2016;30(1):150-63.
Low, 2017	Important differential in a patient presenting with neuropsychiatric symptoms: Anti-N-Methyl-D-Aspartate receptor encephalitis.<br>Low JM.<br>Med J Malaysia. 2017 Oct;72(5):306-307.
Lu - Lu, 2016	Brainstem and vestibulocochlear nerve involvement in relapsing-remitting anti-NMDAR encephalitis.<br>Lu T, Cai W, Qiu W, Sun X, Lu Z.<br>Neurol Sci. 2016 Jan;37(1):149-51.
Lu - Ram, 2015	Acute psychosis in a pregnant patient with Graves' hyperthyroidism and anti-NMDA receptor encephalitis.<br>Lu J, Samson S, Kass J, Ram N.<br>BMJ Case Rep. 2015 Apr 22;2015.
Luca - Benseler, 2011	Anti-N-methyl-D-aspartate receptor encephalitis: a newly recognized inflammatory brain disease in children.<br>Luca N, Daengsuwan T, Dalmau J, Jones K, deVeber G, Kobayashi J, Laxer RM, Benseler SM.<br>Arthritis Rheum. 2011 Aug;63(8):2516-22.
Luo - Huang, 2016	Anti-N-methyl-d-aspartate receptor encephalitis in a patient with neuromyelitis optica spectrum disorders.<br>Luo JJ, Lv H, Sun W, Zhao J, Hao HJ, Gao F, Huang YN.<br>Mult Scler Relat Disord. 2016 Jul;8:74-7.
Lwanga - Lastra, 2018	Occult teratoma in a case of N-methyl-D-aspartate receptor encephalitis.<br>Lwanga A, Kamson DO, Wilkins TE, Sharma V, Schulte JJ, Miller J, Hassan I, Lastra RR.<br>Neuroradiol J. 2018 Aug;31(4):415-419.
Maccaferri - Berney, 2016	Anti-N-Methyl-D-Aspartate Receptor Encephalitis: A New Challenging Entity for Consultation-Liaison Psychiatrist.<br>Maccaferri GE, Rossetti AO, Dalmau J, Berney A.<br>Brain Disord Ther. 2016 May;5(2).
MacMahon - Rhodes, 2013	Ketamine Infusion Associated with Improved Neurology in a Patient with NMDA Receptor Encephalitis.<br>MacMahon M, Naysmith MR, McCallion S, Rhodes J.<br>Case Rep Crit Care. 2013;2013:383125.
Maeder – Ingvar, 2011	FDG-PET hyperactivity in basal ganglia correlating with clinical course in anti-NDMA-R antibodies encephalitis.<br>Maeder-Ingvar M, Prior JO, Irani SR, Rey V, Vincent A, Rossetti AO.<br>J Neurol Neurosurg Psychiatry. 2011 Feb;82(2):235-6. d
Maggina – Mastroyianni, 2012	Anti-N-methyl-D-aspartate receptor encephalitis presenting with acute psychosis in a preteenage girl: a case report.<br>Maggina P, Mavrikou M, Karagianni S, Skevaki CL, Triantafyllidou A, Voudris C, Katsarou E, Stamogiannou L,

© 2021 Nosadini M et al. *JAMA Neurology*.

	Mastroyianni S.<br>J Med Case Rep. 2012 Jul 30;6:224.
Maggio - Cimaz, 2017	Atypical presentation of anti-N-methyl-D-aspartate receptor encephalitis: two case reports.<br>Maggio MC, Mastrangelo G, Skabar A, Ventura A, Carrozzi M, Santangelo G, Vanadia F, Corsello G, Cimaz R.<br>J Med Case Rep. 2017 Aug 16;11(1):225.
Magley – Apperson, 2012	Pregnancy outcome in anti-N-methyl-D-aspartate receptor encephalitis.<br>Magley J, Towner D, Taché V, Apperson ML.<br>Obstet Gynecol. 2012 Aug;120(2 Pt 2):480-3.
Makuch - Irani, 2018	N-methyl-D-aspartate receptor antibody production from germinal center reactions: Therapeutic implications.<br>Makuch M, Wilson R, Al-Diwani A, Varley J, Kienzler AK, Taylor J, Berretta A, Fowler D, Lennox B, Leite MI, Waters P, Irani SR.<br>Ann Neurol. 2018 Mar;83(3):553-561.
Malayev - Imlay, 2015	Immature Teratoma Associated With Anti-N-Methyl-D-Aspartate Receptor Encephalitis.<br>Malayev Y, Alberts J, Verardi MA, Mattison AR, Imlay S.<br>J Am Osteopath Assoc. 2015 Sep;115(9):573-7.
Mangalwedhe - Dugani, 2015	Anti-N-Methyl-D-Aspartate Receptor Encephalitis Presenting With Psychiatric Symptoms.<br>Mangalwedhe SB, Pandurangi AA, Pandurangi AK, Dugani RI.<br>J Neuropsychiatry Clin Neurosci. 2015;27(2):e152-3.
Mann – Afzal, 2012	A multidisciplinary approach to the treatment of anti-NMDA-receptor antibody encephalitis: a case and review of the literature.<br>Mann A, Machado NM, Liu N, Mazin AH, Silver K, Afzal KI.<br>J Neuropsychiatry Clin Neurosci. 2012 Spring;24(2):247-54.
Mantere - Suvisaar, 2018	Anti-neuronal anti-bodies in patients with early psychosis.<br>Mantere O, Saarela M, Kieseppä T, Raij T, Mäntylä T, Lindgren M, Rikandi E, Stoecker W, Teegen B, Suvisaari J.<br>Schizophr Res. 2018 Feb;192:404-407.
Maqbool – Chugani, 2011	Novel FDG-PET findings in anti-NMDA receptor encephalitis: a case based report.<br>Maqbool M, Oleske DA, Huq AH, Salman BA, Khodabakhsh K, Chugani HT.<br>J Child Neurol. 2011 Oct;26(10):1325-8.
Maramattom – Sundaram, 2010	Idiopathic anti-NMDA-receptor encephalitis in a young Indian girl.<br>Maramattom BV, Philip C, Sundaram PS.<br>Neurol India. 2010 Jul-Aug;58(4):671-2.
Maramattom – Jacob, 2011	N-methyl D-aspartate receptor encephalitis: A new addition to the spectrum of autoimmune encephalitis.<br>Maramattom BV, Jacob A.<br>Ann Indian Acad Neurol. 2011 Jul;14(3):153-7.
Mariotto - Ferrari, 2017	Persistence of anti-NMDAR antibodies in CSF after recovery from autoimmune encephalitis.<br>Mariotto S, Andreetta F, Farinazzo A, Monaco S, Ferrari S.<br>Neurol Sci. 2017 Aug;38(8):1523-1524.
Mariotto - Monaco, 2014	Anti-N-methyl-d-aspartate receptor encephalitis causing a prolonged depressive disorder evolving to inflammatory brain disease.<br>Mariotto S, Tamburin S, Salviati A, Ferrari S, Zoccarato M, Giometto B, Bertolasi L, Alessandrini F, Benedetti MD, Monaco S.<br>Case Rep Neurol. 2014 Feb 8;6(1):38-43.
Marques - Sales, 2014	Anti-NMDA receptor encephalitis presenting with total insomnia--a case report.<br>Marques IB, Teotónio R, Cunha C, Bento C, Sales F.<br>J Neurol Sci. 2014 Jan 15;336(1-2):276-80.
Martínez – Cartier, 2012	[Limbic encephalitis with positive anti-N-methyl-D-aspartate antibodies. Report of one case].<br>Martínez DB, Guerrero RT, Grandjean MB, Cartier LR.<br>Rev Med Chil. 2012 Sep;140(9):1170-3.
Martinez -Camara-Lemarroy, 2018	Lyme borreliosis as a trigger for NMDA receptor encephalitis?<br>Martinez HR, Olguin-Ramirez LA, Camara-Lemarroy CR.<br>Neurol Sci. 2018 Jul 14
Martinez-Hernandez, 2011	Analysis of complement and plasma cells in the brain of patients with anti-NMDAR encephalitis.<br>Martinez-Hernandez E, Horvath J, Shiloh-Malawsky Y, Sangha N, Martinez-Lage M, Dalmau J.<br>Neurology. 2011 Aug 9;77(6):589-93.
Martín – Viota, 2012	[Anti-NMDA receptor encephalitis in a 3-year-old girl with no associated pathology involving a tumour].<br>Martin-Viota L, Garcia-Conde M, Solis-Reyes C, Duque-Fernandez MR, Lopez-Mendoza S.<br>Rev Neurol. 2012 Nov 16;55(10):593-7. Spanish.
Masghati - Dorigo, 2014	Anti-N-methyl-aspartate receptor encephalitis in identical twin sisters: role for oophorectomy.<br>Masghati S, Nosratian M, Dorigo O.<br>Obstet Gynecol. 2014 Feb;123(2 Pt 2 Suppl 2):433-5.
Mathai - Janssen, 2016	Scratching Below the Surface.<br>Mathai SK, Josephson SA, Badlam J, Saint S, Janssen WJ.<br>N Engl J Med. 2016 Dec 1;375(22):2188-2193.
Mathis - Neau, 2015	Anti-NMDA Receptor Encephalitis During Pregnancy: A Case Report.<br>Mathis S, Pin JC, Pierre F, Ciron J, Iljicsov A, Lamy M, Neau JP.<br>Medicine (Baltimore). 2015 Jul;94(26):e1034.
Matricardi - Granata, 2016	Cognitive and neuropsychological evolution in children with anti-NMDAR encephalitis.<br>Matricardi S, Patrini M, Freri E, Ragona F, Zibordi F, Andreetta F, Nardocci N, Granata T.<br>J Neurol. 2016 Apr;263(4):765-71.

© 2021 Nosadini M et al. *JAMA Neurology*.

Matsumoto – Kato, 2012	Electroconvulsive therapy can improve psychotic symptoms in anti-NMDA-receptor encephalitis.<br>Matsumoto T, Matsumoto K, Kobayashi T, Kato S.<br>Psychiatry Clin Neurosci. 2012 Apr;66(3):242-3.
Matsumoto - Takahashi, 2017	Dystonic Seizures and Intense Hyperperfusion of the Basal Ganglia in a Patient with Anti-N-Methyl-D-Aspartate Receptor Encephalitis.<br>Matsumoto H, Hashida H, Takahashi Y.<br>Case Rep Neurol. 2017 Nov 23;9(3):272-276.
McCarthy - O'Rourke, 2012	Anti-NMDA receptor encephalitis with associated catatonia during pregnancy.<br>McCarthy A, Dineen J, McKenna P, Keogan M, Sheehan J, Lynch T, O'Rourke K.<br>J Neurol. 2012 Dec;259(12):2632-5.
McCoy - Go, 2011	Autoimmune limbic encephalitis as an emerging pediatric condition: case report and review of the literature.<br>McCoy B, Akiyama T, Widjaja E, Go C.<br>J Child Neurol. 2011 Feb;26(2):218-22.
McIvor - Moore, 2017	Spontaneous recovery of memory functions in an untreated case of anti NMDAR encephalitis - a reason to maintain hope.<br>McIvor K, Moore P.<br>Clin Neuropsychol. 2017 Jan;31(1):289-300.
McKeon - Robinson, 2016	Cognitive and Social Functioning Deficits after Anti-N-Methyl-D-Aspartate Receptor Encephalitis: An Exploratory Case Series.<br>McKeon GL, Scott JG, Spooner DM, Ryan AE, Blum S, Gillis D, Langguth D, Robinson GA.<br>J Int Neuropsychol Soc. 2016 Sep;22(8):828-38.
Mechelhoff - Winter, 2015	Anti-NMDA receptor encephalitis presenting as atypical anorexia nervosa: an adolescent case report.<br>Mechelhoff D, van Noort BM, Weschke B, Bachmann CJ, Wagner C, Pfeiffer E, Winter S.<br>Eur Child Adolesc Psychiatry. 2015 Nov;24(11):1321-4.
Medepalli - Elwing, 2016	Psychosis: call a surgeon? A rare etiology of psychosis requiring resection.<br>Medepalli K, Lee CM, Benninger LA, Elwing JM.<br>SAGE Open Med Case Rep. 2016 Sep 21;4:2050313X16670084. eCollection 2016.
Medina - Cooper, 2017	Refractory Catatonia Due to N-methyl-D-Aspartate Receptor Encephalitis Responsive to Electroconvulsive Therapy: The Clinical Use of the Clock Drawing Test.<br>Medina M, Cooper JJ.<br>J ECT. 2017 Dec;33(4):223-224.
Mehr - Kumar, 2016	Profound Autonomic Instability Complicated by Multiple Episodes of Cardiac Asystole and Refractory Bradycardia in a Patient with Anti-NMDA Encephalitis.<br>Mehr SR, Neeley RC, Wiley M, Kumar AB.<br>Case Rep Neurol Med. 2016;2016:7967526.
Menon - Thomas, 2018	Clinical-radiological-pathological correlation in an unusual case of refractory epilepsy: a two-year journey of whodunit!<br>Menon D, Menon RN, Kesavadas C, Mahadevan A, Radhakrishnan A, Kannoth S, Nair PP, Abraham M, Thomas B, Thomas SV.<br>Epileptic Disord. 2018 Feb 1;20(1):51-59
Mesquita – Siva, 2011	Anti-NMDA receptor encephalitis suspected as cause of drug-induced psychosis.<br>Mesquita J, Siva L.<br>J Neuropsychiatry Clin Neurosci. 2011 Fall;23(4):E2.
Metzger -Desestret, 2018	Balint syndrome in anti-NMDA receptor encephalitis.<br>Metzger A, Pisella L, Vighetto A, Joubert B, Honnorat J, Tilikete C, Desestret V.<br>Neurol Neuroimmunol Neuroinflamm. 2018 Dec 13;6(1):e532.
Miao - Wang, 2018	Ictal Rhythmic Alpha Sinusoidal Waves in 3 Cases of Anti-NMDAR Encephalitis.<br>Miao A, Wang X.<br>Clin EEG Neurosci. 2018 Sep;49(5):302-305.
Millichap – Wainwright, 2011	Ictal asystole and anti-N-methyl-D-aspartate receptor antibody encephalitis.<br>Millichap JJ, Goldstein JL, Laux LC, Nordli DR Jr, Stack CV, Wainwright MS.<br>Pediatrics. 2011 Mar;127(3):e781-6.
Milovac - Filipčić, 2016	Acute psychosis - anti-NMDA receptor encephalitis phase.<br>Milovac Ž, Santini M, Pisk SV, Caratan S, Grošić V, Filipčić I.<br>Psychiatr Danub. 2016 Sep;28(3):301-303.
Mimbella - Podobinski, 2016	Poster 260 The Rehabilitative Management of a Patient with Anti-N-methyl-D-aspartate Receptor Encephalitis: A Case Report.<br>Mimbella PC, Verduzco-Gutierrez M, Irvine MJ, Podobinski TK.<br>PM R. 2016 Sep;8(9S):S244-S245.
Mirabelli – Badenier, 2014	Anti-NMDAR encephalitis misdiagnosed as Hashimoto's encephalopathy.<br>Mirabelli-Badenier M, Biancheri R, Morana G, Fornarino S, Siri L, Celle ME, Veneselli E, Vincent A, Gaggero R, Mancardi MM.<br>Eur J Paediatr Neurol. 2014 Jan;18(1):72-4.
Mirza – Richa, 2011	Adjunct therapeutic plasma exchange for anti-N-methyl-D-aspartate receptor antibody encephalitis: a case report and review of literature.<br>Mirza MK, Pogoriler J, Paral K, Ananthanarayanan V, Mandal S, Mazin A, Baron B, Richa E.<br>J Clin Apher. 2011 Dec;26(6):362-5.
Mitani – Osaka, 2013	[An 8-year-old boy with anti-NMDA receptor encephalitis, successfully treated with cyclophosphamide].<br>Mitani T, Ohtsuka Y, Yamamoto K, Watanabe Y, Tsuji M, Samejima K, Aida N, Sato T, Wada T, Osaka H.<br>No To Hattatsu. 2013 Jan;45(1):53-7. Japanese.

© 2021 Nosadini M et al. *JAMA Neurology*.

Mitra – Afify, 2018	Ovarian teratoma associated Anti-N-methyl-D-aspartate receptor encephalitis: a difficult diagnosis with a favorable prognosis.<br>Mitra AD, Afify A.<br>Autops Case Rep. 2018 Apr 18;8(2):e2018019.
Miyauchi - Yamagata, 2016	A case of anti-NMDAR encephalitis presented hypotensive shock during plasma exchange.<br>Miyauchi A, Monden Y, Osaka H, Takahashi Y, Yamagata T.<br>Brain Dev. 2016 Apr;38(4):427-30.
Mizutamari - Katabuchi, 2016	Successful outcome following detection and removal of a very small ovarian teratoma associated with anti-NMDA receptor encephalitis during pregnancy.<br>Mizutamari E, Matsuo Y, Namimoto T, Ohba T, Yamashita Y, Katabuchi H.<br>Clin Case Rep. 2016 Jan 8;4(3):223-5.
Mohammad - Dale, 2016	Symptomatic treatment of children with anti-NMDAR encephalitis.<br>Mohammad SS, Jones H, Hong M, Nosadini M, Sharpe C, Pillai SC, Brilot F, Dale RC.<br>Dev Med Child Neurol. 2016 Apr;58(4):376-84.
Mohammad, Wallace - Dale, 2014	Antipsychotic-induced akathisia and neuroleptic malignant syndrome in anti-NMDAR encephalitis.<br>Mohammad SS, Wallace G, Ramanathan S, Brilot F, Dale RC.<br>Ann Clin Psychiatry. 2014 Nov;26(4):297-8.
Monteiro - das Neves, 2015	Managing severe behavioral symptoms of a patient with anti-NMDAR encephalitis: case report and findings in current literature.<br>Monteiro VL, Barreto FJ, Rocha PM, do Prado PH, Garcia FD, Correa H, das Neves MC.<br>Trends Psychiatry Psychother. 2015 Jan-Mar;37(1):47-50.
Moss - Sherwood, 2018	Two Case Reports of Neuropsychological Outcomes following Pediatric anti-N-methyl D-aspartate Receptor Autoimmune Encephalitis.<br>Moss N, Petranovich CL, Parks L, Sherwood A.<br>Dev Neuropsychol. 2018 Aug 10:1-13.
Motoyama - Tanaka, 2010	[Anti-NMDA receptor antibody encephalitis with recurrent optic neuritis and epilepsy].<br>Motoyama R, Shiraishi K, Tanaka K, Kinoshita M, Tanaka M.<br>Rinsho Shinkeigaku. 2010 Aug;50(8):585-8. Japanese.
Motta – Stęposz, 2012	[Anti-NMDA receptor encephalitis - case report].<br>Motta E, Gołba A, Kazibutowska Z, Huć M, Stęposz A.<br>Neurol Neurochir Pol. 2012 May-Jun;46(3):288-93. Polish.
Moura - Talina, 2016	First-episode psychosis in a 15 year-old female with clinical presentation of anti-NMDA receptor encephalitis: a case report and review of the literature.<br>Moura M, Silva-Dos-Santos A, Afonso J, Talina M.<br>BMC Res Notes. 2016 Jul 29;9:374.
Murdie - Ferguson, 2016	Seronegative Anti-N-Methyl-D-Aspartate Receptor Encephalitis.<br>Murdie D, Cooney G, Ferguson J.<br>Biol Psychiatry. 2016 May 1;79(9):e67-8.
Mutti -Caffarra, 2017	A case of reversible anti-NMDA-receptor encephalitis: neuropsychological and neuroradiological features.<br>Mutti C, Barocco F, Zinno L, Negrotti A, Spallazzi M, Pavesi G, Gardini S, Caffarra P.<br>Neurol Sci. 2017 Dec;38(12):2231-2236.
Mythri -Mathew, 2016	Catatonic Syndrome in Anti-NMDA Receptor Encephalitis.<br>Mythri SV, Mathew V.<br>Indian J Psychol Med. 2016 Mar-Apr;38(2):152-4.
Naeije – Legros, 2010	Acute behavioural change in a young woman evolving towards cerebellar syndrome.<br>Naeije G, de Hemptinne Q, Depondt C, Pandolfo M, Legros B.<br>Clin Neurol Neurosurg. 2010 Jul;112(6):509-11.
Nagata - Mitsuo, 2018	[A case of anti-myelin oligodendrocyte glycoprotein (MOG) and anti-N-methyl-D-aspartate (NMDA) receptor antibody-positive encephalitis with optic neuritis].<br>Nagata S, Nishimura Y, Mitsuo K.<br>Rinsho Shinkeigaku. 2018 Oct 24;58(10):636-641.
Naoura – Koskas, 2011	Anti-N-methyl-D-aspartate receptor encephalitis complicating ovarian teratomas: a case report.<br>Naoura I, Didelot A, Walker F, Luton D, Koskas M.<br>Am J Obstet Gynecol. 2011 Oct;205(4):e6-8.
Nasky, 2008	Psychosis associated with anti-N-methyl-D-aspartate receptor antibodies.<br>Nasky KM, Knittel DR, Manos GH.<br>CNS Spectr. 2008 Aug;13(8):699-703.
Nath – Ali, 2011	NMDA receptor encephalitis--expanding the clinical spectrum.<br>Nath U, Warren NM, Ali H.<br>BMJ Case Rep. 2011 Jul 28;2011.
Nazif - Dizon, 2012	Anti-N-methyl-D-aspartate receptor encephalitis: an emerging cause of centrally mediated sinus node dysfunction.<br>Nazif TM, Vázquez J, Honig LS, Dizon JM.<br>Europace. 2012 Aug;14(8):1188-94.
Neiman - Chokroverty, 2015	Bulbar and Limb Myorhythmia with "Smooch Sign": A Distinctive Movement Disorder in an Adult Patient with Reversible Anti-NMDA Receptor Encephalitis Associated with an Ovarian Teratoma.<br>Neiman ES, Panezai S, Salim S, Seyffert M, Leifer LE, Rosenberg M, Chokroverty S.<br>Neurodiagn J. 2015 Sep;55(3):149-56.
Newey - Hantus, 2016	[(18)F]-Fluoro-Deoxy-Glucose Positron Emission Tomography Scan Should Be Obtained Early in Cases of Autoimmune Encephalitis.

© 2021 Nosadini M et al. *JAMA Neurology*.

	Newey CR, Sarwal A, Hantus S.<br>Autoimmune Dis. 2016;2016:9450452.
Ng - Mirsattari, 2018	Teratoma-negative anti-NMDA receptor encephalitis presenting with a single generalized tonic-clonic seizure.<br>Ng AC, Tripic M, Mirsattari SM.<br>Epilepsy Behav Case Rep. 2018 Mar 13;10:29-31.
Nichols, 2016	Anti-NMDA receptor encephalitis: An emerging differential diagnosis in the psychiatric community.<br>Nichols TA.<br>Ment Health Clin. 2016 Nov 3;6(6):297-303.
Niehusmann - Bien, 2009	Diagnostic value of N-methyl-D-aspartate receptor antibodies in women with new-onset epilepsy.<br>Niehusmann P, Dalmau J, Rudlowski C, Vincent A, Elger CE, Rossi JE, Bien CG.<br>Arch Neurol. 2009 Apr;66(4):458-64.
Nijmeijer – Coutinho, 2014	Anti-N-methyl-D-aspartate receptor encephalitis in a pre-teenage girl: a case report.<br>Nijmeijer S, Bontemps S, Naeije L, Coutinho J.<br>Eur J Pediatr. 2014 May;173(5):681-3.
Niu - Guo, 2017	Paraneoplastic Anti-N-Methyl-D-Aspartic Acid Receptor Encephalitis.<br>Niu C, Guo W.<br>Chin Med J (Engl). 2017 Nov 20;130(22):2765-2766.
Noble - Lancaster, 2018	Anti-NMDAR encephalitis in a patient with Crohn disease receiving adalimumab.<br>Noble GP, Lancaster E.<br>Neurol Neuroimmunol Neuroinflamm. 2018 Jul 2;5(5):e476.
Nolan - Carr, 2014	Anti-N-methyl-D-aspartate receptor (anti-NMDAR) encephalitis presenting to the emergency department with status epilepticus.<br>Nolan B, Plenk K, Carr D.<br>CJEM. 2014 Sep;16(5):425-8.
Nosadini - Sartori, 2014	Longitudinal electroencephalographic (EEG) findings in pediatric anti-N-methyl-D-aspartate (anti-NMDA) receptor encephalitis: the Padua experience.<br>Nosadini M, Boniver C, Zuliani L, de Palma L, Cainelli E, Battistella PA, Toldo I, Suppiej A, Sartori S.<br>J Child Neurol. 2015 Feb;30(2):238-45.
Nunez-Enamorado – Graus, 2012	Fast and spectacular clinical response to plasmapheresis in a paediatric case of anti-NMDA encephalitis].<br>Nunez-Enamorado N, Camacho-Salas A, Belda-Hofheinz S, Cordero-Castro C, Simon-De Las Heras R, Saiz-Diaz R, Martinez-Sarries FJ, Martinez-Menendez B, Graus F.<br>Rev Neurol. 2012 Apr 1;54(7):420-4.
Ochoa-Figueroa - Desequera-Rahola, 2012	[Changes in cerebral metabolism detected by (18)F-FDG PET-CT in a case of anti-NMDA receptor encephalitis].<br>Ochoa-Figueroa MA, Cárdenas-Negro C, Allende-Riera A, Uña-Gorospe J, Cabello García D, Desequera-Rahola M.<br>Rev Esp Med Nucl Imagen Mol. 2012 Jul-Aug;31(4):219-22
Odriozola-Grijalba – Graus, 2011	[Anti-NMDA receptor encephalitis in a four-year-old male].<br>Odriozola-Grijalba M, Galé-Ansó I, López-Pisón J, Monge-Galindo L, García-Iñiguez JP, Madurga-Revilla P, Graus F.<br>Rev Neurol. 2011 Jul 1;53(1):58-60. Spanish. No abstract available.
Ohe – Araki, 2011	[A case of occipital epilepsy with anti-GluRepsilon2 antibody in cerebrospinal fluid, presenting as repeated visual disturbance and headache].<br>Ohe Y, Nakazato Y, Ohkuma A, Tamura N, Takahashi Y, Araki N.<br>Rinsho Shinkeigaku. 2011 Jul;51(7):505-9. Japanese.
Okanishi - Enoki, 2018	Epileptic spasms secondary to acute cerebral and cerebellar encephalitis.<br>Okanishi T, Fujimoto A, Hashimoto R, Nishimura M, Kanai S, Ogawa M, Suzuki T, Motoi H, Takahashi Y, Enoki H.<br>Brain Dev. 2018 Mar;40(3):218-221.
Omata - Shimojo, 2017	Ovarian teratoma development after anti-NMDA receptor encephalitis treatment.<br>Omata T, Kodama K, Watanabe Y, Iida Y, Furusawa Y, Takashima A, Takahashi Y, Sakuma H, Tanaka K, Fujii K, Shimojo N.<br>Brain Dev. 2017 May;39(5):448-451.
Omura - Ohta, 2014	Anti-NMDAR encephalitis: case report and diagnostic issues.<br>Omura T, Sonoda S, Nagata K, Okita T, Hoshiai A, Sano H, Tanaka Y, Tsurukiri J, Arai T, Ohta S.<br>Acute Med Surg. 2014 Sep 8;2(1):56-59.
Orengo - Cree, 2015	Simultaneous serum aquaporin-4 antibody and CSF NMDA receptor antibody-positive encephalitis.<br>Orengo JP, Pekmezci M, Cree BA.<br>Neurol Neuroimmunol Neuroinflamm. 2015 Apr 9;2(3):e101.
Osei-Lah - Kirkham, 2014	Focal EEG slowing and chorea: electroclinical clues to the diagnosis of anti-NMDAR encephalitis.<br>Osei-Lah A, Durrant E, Hussain M, Kirkham F.<br>Epileptic Disord. 2014 Dec;16(4):482-5.
Ozelle - Riquin, 2017	[Organic troubles with psychiatric symptoms: What is the appropriate childhood and adolescence psychiatric care? Reflections on disimmune encephalitis cases].<br>Ozelle R, Doudard A, Bodin AL, Gueden S, Duverger P, Riquin E.<br>Arch Pediatr. 2017 May;24(5):483-491.
Padma – Maramatton, 2011	PET/CT in the evaluation of anti-NMDA-receptor encephalitis: What we need to know as a NM physician.<br>Padma S, Sundaram PS, Marmattom BV.<br>Indian J Nucl Med. 2011 Apr;26(2):99-101.

© 2021 Nosadini M et al. *JAMA Neurology*.

Palakkuzhiyil - Shihabudheen, 2018	Anti-NMDA-receptor encephalitis presenting with catatonia in a middle aged male.<br>Palakkuzhiyil N, Uvais NA, Moideen S, Shihabudheen P.<br>Asian J Psychiatr. 2018 Apr 27;35:26-27.
Pandit - Tripathi, 2013	Autoimmune encephalitis: A potentially reversible cause of status epilepticus, epilepsy, and cognitive decline.<br>Pandit AK, Ihtisham K, Garg A, Gulati S, Padma MV, Tripathi M.<br>Ann Indian Acad Neurol. 2013 Oct;16(4):577-84.
Parfene - Gordon-Elliott, 2016	The Neurocognitive Profile of an Anti-N-Methyl-D-Aspartate Receptor Encephalitis Patient Presenting With Neuropsychiatric Symptoms.<br>Parfene C, Lipira C, Gunning F, Gordon-Elliott JS.<br>J Neuropsychiatry Clin Neurosci. 2016 Summer;28(3):255-6.
Pascual-Ramírez - García-Serrano, 2011	Anesthetic management of ovarian teratoma excision associated with anti-N-methyl-D-aspartate receptor encephalitis.<br>Pascual-Ramírez J, Muñoz-Torrero JJ, Bacci L, Trujillo SG, García-Serrano N.<br>Int J Gynaecol Obstet. 2011 Dec;115(3):291-2.
Passareli - Rocha, 2016	Early occurrence of brief potentially ictal rhythmic discharges [B(I)RDs], and subsequent emergence of asymmetric extreme delta brush (EDB) in Anti-NMDA receptors encephalitis.<br>Passareli V, Medeiros L, Simabukuro M, Baldocci M, Brucki S, Rocha MS.<br>Arq Neuropsiquiatr. 2016 Feb;74(2):177.
Patarata - Moraes-Fontes, 2016	Anti-N-Methyl-D-Aspartate Receptor Encephalitis in HIV Infection.<br>Patarata E, Bernardino V, Martins A, Pereira R, Loureiro C, Moraes-Fontes MF.<br>Case Rep Neurol. 2016 Dec 13;8(3):251-257.
Peacock - Syed, 2016	Poster 461 Recovery in Acute Inpatient Rehabilitation for Pediatric Anti-N-methyl-D-aspartate Receptor Encephalitis: A Case Report.<br>Peacock J, Amin I, Syed Ahmed.<br>PM R. 2016 Sep;8(9S):S310-S311.
Pellkofer – Derfuss, 2010	Non-paraneoplastic limbic encephalitis associated with NMDAR and VGKC antibodies.<br>Pellkofer HL, Kuempfel T, Jacobson L, Vincent A, Derfuss T.<br>J Neurol Neurosurg Psychiatry. 2010 Dec;81(12):1407-8.
Peng - Wang, 2017	Anti-N-methyl-D-aspartate receptor encephalitis associated with intracranial Angiostrongylus cantonensis infection: a case report.<br>Peng Y, Liu X, Pan S, Xie Z, Wang H.<br>Neurol Sci. 2017 Apr;38(4):703-706.
Pennington - Razvi, 2012	N-methyl D-aspartate receptor antibody encephalitis associated with myelitis.<br>Pennington C, Livingstone S, Santosh C, Razvi S.<br>J Neurol Sci. 2012 Jun 15;317(1-2):151-3.
Perogamvros – Leemann, 2012	The role of NMDA receptors in human eating behavior: evidence from a case of anti-NMDA receptor encephalitis.<br>Perogamvros L, Schnider A, Leemann B.<br>Cogn Behav Neurol. 2012 Jun;25(2):93-7.
Pham - Schwartz, 2011	Therapeutic plasma exchange for the treatment of anti-NMDA receptor encephalitis.<br>Pham HP, Daniel-Johnson JA, Stotler BA, Stephens H, Schwartz J.<br>J Clin Apher. 2011 Dec;26(6):320-5.
Phan - Delwart, 2016	A new densovirus in cerebrospinal fluid from a case of anti-NMDA-receptor encephalitis.<br>Phan TG, Messacar K, Dominguez SR, da Costa AC, Deng X, Delwart E.<br>Arch Virol. 2016 Nov;161(11):3231-5.
Phillips - DiFazio, 2017	Hemiataxia: A Novel Presentation of Anti-NMDA Receptor Antibody Mediated Encephalitis in an Adolescent.<br>Phillips GD, Jones GN, Callaghan M, DiFazio MP.<br>Case Rep Psychiatry. 2017;2017:1310465.
Pillai - Dale, 2010	Cortical hypometabolism demonstrated by PET in relapsing NMDA receptor encephalitis.<br>Pillai SC, Gill D, Webster R, Howman-Giles R, Dale RC.<br>Pediatr Neurol. 2010 Sep;43(3):217-20.
Pinho - Beleza, 2012	Diversity in anti-N-methyl-D-aspartate receptor encephalitis: case-based evidence.<br>Pinho J, Rocha J, Rodrigues M, Pereira J, Maré R, Ferreira C, Lourenço E, Beleza P.<br>Psychiatry Clin Neurosci. 2012 Mar;66(2):153-6.
Player - Croix, 2015	Pediatric Opsoclonus-Myoclonus-Ataxia Syndrome Associated With Anti-N-methyl-D-aspartate Receptor Encephalitis.<br>Player B, Harmelink M, Bordini B, Weisgerber M, Girolami M, Croix M.<br>Pediatr Neurol. 2015 Nov;53(5):456-8.
Poduval	Immune-mediated encephalitis and virilization in association with a mature cystic ovarian teratoma in an adolescent girl.<br>Poduval A, Antal Z, Lee T, Bar A, Dalmau J, Muzumdar R.<br>Horm Res. 2009;72(4):252-6.
Poloni - Deonna, 2010	Severe childhood encephalopathy with dyskinesia and prolonged cognitive disturbances: evidence for anti-N-methyl-D-aspartate receptor encephalitis.<br>Poloni C, Korff CM, Ricotti V, King MD, Perez ER, Mayor-Dubois C, Haenggeli CA, Deonna T.<br>Dev Med Child Neurol. 2010 May;52(5):e78-82.
Power - Altman, 2014	Tubal teratoma causing anti-NMDAR encephalitis.<br>Power L, James J, Masoud I, Altman A.<br>J Obstet Gynaecol Can. 2014 Dec;36(12):1093-1096.
Probasco - Kaplan, 2014	Electroencephalographic and fluorodeoxyglucose-positron emission tomography correlates in anti-N-methyl-d-aspartate receptor autoimmune encephalitis.

© 2021 Nosadini M et al. *JAMA Neurology*.

	Probasco JC, Benavides DR, Ciarallo A, Sanin BW, Wabulya A, Bergey GK, Kaplan PW.<br>Epilepsy Behav Case Rep. 2014 Oct 10;2:174-8.
Pruss - Ebinger, 2014	A case of inflammatory peripheral nerve destruction antedating anti-NMDA receptor encephalitis.<br>Prüss H, Hoffmann C, Stenzel W, Saschenbrecker S, Ebinger M.<br>Neurol Neuroimmunol Neuroinflamm. 2014 May 22;1(2):e14.
Prüss - Wandinger, 2010	Retrospective analysis of NMDA receptor antibodies in encephalitis of unknown origin.<br>Prüss H, Dalmau J, Harms L, Höltje M, Ahnert-Hilger G, Borowski K, Stoecker W, Wandinger KP.<br>Neurology. 2010 Nov 9;75(19):1735-9.
Punja - Schwartz, 2013	Anti-N-methyl-D-aspartate receptor (anti-NMDAR) encephalitis: an etiology worth considering in the differential diagnosis of delirium.<br>Punja M, Pomerleau AC, Devlin JJ, Morgan BW, Schier JG, Schwartz MD.<br>Clin Toxicol (Phila). 2013 Sep-Oct;51(8):794-7.
Qin, Wu - Zheng, 2017	Anti-N-methyl-D-aspartate receptor(NMDAR) antibody encephalitis presents in atypical types and coexists with neuromyelitis optica spectrum disorder or neurosyphilis.<br>Qin K, Wu W, Huang Y, Xu D, Zhang L, Zheng B, Jiang M, Kou C, Gao J, Li W, Zhang J, Wang S, Luan Y, Yan C, Xu D, Zheng X.<br>BMC Neurol. 2017 Jan 5;17(1):1
Raha - Udani, 2012	Nonparaneoplastic anti-N-methyl-D-aspartate receptor encephalitis: a case series of four children.<br>Raha S, Gadgil P, Sankhla C, Udani V.<br>Pediatr Neurol. 2012 Apr;46(4):246-9.
Rainey - Cheesman, 2014	Anti-NMDA receptor encephalitis: an easily missed diagnosis in older patients.<br>Rainey K, Gholkar B, Cheesman M.<br>Age Ageing. 2014 Sep;43(5):725-6.
Rajahram - Menon, 2015	Anti N-Methyl-D-Aspartate receptor encephalitis: An under-recognised cause of encephalitis.<br>Rajahram GS, Nadarajah R, Lim KS, Menon J.<br>Med J Malaysia. 2015 Dec;70(6):363-4.
Ramanathan - Fung, 2013	Long duration between presentation of probable anti-N-methyl-D-aspartate receptor encephalitis and either clinical relapse or positive serum autoantibodies.<br>Ramanathan S, Wong CH, Fung VS.<br>J Clin Neurosci. 2013 Sep;20(9):1322-3.
Ramanathan - Fung, 2014	Characterisation of a syndrome of autoimmune adult onset focal epilepsy and encephalitis.<br>Ramanathan S, Bleasel A, Parratt J, Orr C, Dale RC, Vincent A, Fung VS.<br>J Clin Neurosci. 2014 Jul;21(7):1169-75.
Ran - Yu, 2017	Anti-NMDAR encephalitis followed by seropositive neuromyelitis optica spectrum disorder: A case report and literature review.<br>Ran Y, Wang L, Zhang F, Ao R, Dong Z, Yu S.<br>Clin Neurol Neurosurg. 2017 Apr;155:75-82.
Randall - Larner, 2018	Autoimmune encephalitis (NMDAR antibody) in a patient receiving chronic post-transplant immunosuppression.<br>Randall A, Huda S, Jacob A, Larner AJ.<br>Pract Neurol. 2018 Aug;18(4):320-322.
Rangel-Guerra - Galarza-Delgado, 2015	Could coenzyme Q10 supplementation have a role in the treatment of anti-NMDA receptor encephalitis?<br>Rangel-Guerra R, Camara-Lemarroy CR, Garcia-Arellano G, Rodriguez-Martinez AC, Galarza-Delgado DA.<br>Acta Neurol Belg. 2015 Mar;115(1):85-6.
Rao – Kissell, 2011	An unusual case of altered mental status in a young woman.<br>Rao RM, Chipinapi T, Bharadwaj S, Kissell KA.<br>N Am J Med Sci. 2011 Nov;3(11):518-9.
Raynor - Berkowitz, 2016	Psychosis Secondary to Anti-N-methyl-D-Aspartate Receptor Encephalitis.<br>Raynor G, Bader C, Srikanth M, Kroll D, Gutheil T, Berkowitz A.<br>Harv Rev Psychiatry. 2016 May-Jun;24(3):229-37.
Reddy - Taly, 2018	Anti-NMDA receptor encephalitis presenting as postpartum psychosis-a clinical description and review.<br>Reddy MSS, Thippeswamy H, Ganjekar S, Nagappa M, Mahadevan A, Arvinda HR, Chandra PS, Taly AB.<br>Arch Womens Ment Health. 2018 Aug;21(4):465-469.
Reid – Clardy, 2013	Neurological picture. Anti-NMDA-receptor encephalitis: unusual presentation of an uncommon condition.<br>Reid DK, Clardy SL.<br>J Neurol Neurosurg Psychiatry. 2013 Jan;84(1):69-70.
Reilly-Shapiro - Fendrick, 2016	Anti-NMDA Receptor Encephalitis: A Case Study of Recovery Through Art.<br>Reilly-Shapiro C, Fendrick S.<br>J Pediatr Health Care. 2016 Jan-Feb;30(1):78-83.
Reyes – Botero, 2011	[Anti-NMDA receptor paraneoplastic encephalitis: complete recovery after ovarian teratoma removal].<br>Reyes-Botero G, Uribe CS, Hernandez-Ortiz OE, Ciro J, Guerra A, Dalmau-Obrador J.<br>Rev Neurol. 2011 May 1;52(9):536-40. Spanish.
Reyna-Villasmil – Herrera-Moya, 2017	Anti-N-methyl-D-aspartate-receptor encephalitis due to ovarian teratoma.<br>Reyna-Villasmil E, Mayner-Tresol G, Herrera-Moya P.<br>Med Clin (Barc). 2017 Dec 20;149(12):560-561.
Richard-Mornas, 2011	[N-methyl-d-aspartate receptor antibody encephalitis: a treatable disorder involving B-lymphocytes. A report of two patients].<br>Richard-Mornas A, Jousserand G, Camdessanche JP, Forest F, Paul S, Antoine JC.<br>Rev Med Interne. 2012 Jan;33(1):41-5.

© 2021 Nosadini M et al. *JAMA Neurology*.

Roberts – Swingler, 2012	Not hysteria: ovarian teratoma-associated anti-N-methyl-D-aspartate receptor encephalitis.<br>Roberts R, MacDougall NJ, O'Brien P, Abdelaziz K, Christie J, Swingler R.<br>Scott Med J. 2012 Aug;57(3):182.
Rodriguez-Osorio - Arias, 2014	[Recurrence of encephalitis after 25 years due to anti-NMDA receptor antibodies].<br>Rodriguez-Osorio X, Fernandez-Pajarin G, Arias-Rivas S, Requena-Caballero I, Lopez-Gonzalez FJ, Arias M.<br>Rev Neurol. 2014 Nov 1;59(9):428-9. Review. Spanish.
Rong – Li, 2017	Case report of anti-N-methyl-D-aspartate receptor encephalitis in a middle-aged woman with a long history of major depressive disorder.<br>Rong X, Xiong Z, Cao B, Chen J, Li M, Li Z.<br>BMC Psychiatry. 2017 Aug 31;17(1):320.
Rosenbloom - Tran-Lim, 2018	NMDA receptor antibody encephalitis presenting with enhancing lesion and seizures.<br>Rosenbloom MH, Samuelsson MK, Brogan ME, Tran-Lim TA.<br>Neurol Clin Pract. 2017 Oct;7(5):433-435.
Rozier - King, 2016	Anti-N-Methyl-D-Aspartate Receptor Encephalitis: A Potential Mimic of Neuroleptic Malignant Syndrome.<br>Rozier M, Morita D, King M.<br>Pediatr Neurol. 2016 Oct;63:71-72.
Rutledge - Tubridy, 2016	Anti-NMDA-receptor antibody-mediated cortical blindness: a case report.<br>Rutledge S, Chalissery A, O'Connor R, Mahon S, Connolly S, Farrell M, Crowley P, MacSweeney F, Tubridy N.<br>QJM. 2016 Feb;109(2):127-8.
Ryan – Markx, 2013	Anti-NMDA receptor encephalitis: a cause of acute psychosis and catatonia.<br>Ryan SA, Costello DJ, Cassidy EM, Brown G, Harrington HJ, Markx S.<br>J Psychiatr Pract. 2013 Mar;19(2):157-61.
Rypulak - Czuczwar, 2016	Successful treatment of anti-NMDA receptor encephalitis with a prompt ovarian tumour removal and prolonged course of plasmapheresis: A case report.<br>Rypulak E, Borys M, Piwowarczyk P, Fijalkowska M, Potrec B, Sysiak J, Spustek J, Bartkowska-Sniatkowska A, Kotarski J, Turski WA, Rejdak K, Czuczwar M.<br>Mol Clin Oncol. 2016 Dec;5(6):845-849.
Sachs - Burdette, 2018	Arterial spin labeling perfusion imaging demonstrates cerebral hyperperfusion in anti-NMDAR encephalitis.<br>Sachs JR, Zapadka ME, Popli GS, Burdette JH.<br>Radiol Case Rep. 2017 Jul 24;12(4):833-837.
Sacré - Papo, 2011	Acute psychosis in anti-NMDA-receptor encephalitis.<br>Sacré K, Lidove O, Chanson N, Laganier J, Vidailhet M, Lejoyeux M, Papo T.<br>Presse Med. 2011 Sep;40(9 Pt 1):882-4.
Safadieh – Dabbagh, 2013	Anti-N-methyl-D-aspartate (NMDA) receptor encephalitis in a young Lebanese girl.<br>Safadieh L, Dabbagh O.<br>J Child Neurol. 2013 Oct;28(10):1222-5.
Sakamoto - Nakamura, 2013	Details of treatment-related difficulties in men with anti-N-methyl-D-aspartate receptor encephalitis.<br>Sakamoto H, Hirano M, Samukawa M, Ueno S, Maekura S, Fujimura H, Kuwahara M, Hamada Y, Isono C, Tanaka K, Kusunoki S, Nakamura Y.<br>Eur Neurol. 2013;69(1):21-6.
Sakpichaisakul - Suwannachote, 2018	Heterogenous treatment for anti-NMDAR encephalitis in children leads to different outcomes 6-12 months after diagnosis.<br>Sakpichaisakul K, Patibat L, Wechapinan T, Sri-Udomkajrorn S, Apiwattanakul M, Suwannachote S.<br>J Neuroimmunol. 2018 Nov 15;324:119-125.
Salazar – Abdelhak, 2012	Profuse sialorrhea in a case of anti N-methyl-D-aspartate receptor (NMDAR) encephalitis.<br>Salazar R, James E, Elsayed M, Varelas P, Bartscher J, Corry J, Abdelhak T.<br>Clin Neurol Neurosurg. 2012 Sep;114(7):1066-9.
Salehi - Klein, 2018	A Case of Severe Anti-N-Methyl D-Aspartate (Anti-NMDA) Receptor Encephalitis with Refractory Autonomic Instability and Elevated Intracranial Pressure.<br>Salehi N, Yuan AK, Stevens G, Koshy R, Klein WF.<br>Am J Case Rep. 2018 Oct 12;19:1216-1221.
Salvucci - Sheth, 2014	Pediatric anti-NMDA (N-methyl D-aspartate) receptor encephalitis.<br>Salvucci A, Devine IM, Hammond D, Sheth RD.<br>Pediatr Neurol. 2014 May;50(5):507-10
Sameshima – Saito, 2011	Anti-N-methyl-D-aspartate receptor encephalitis associated with ovarian immature teratoma.<br>Sameshima A, Hidaka T, Shima T, Nakashima A, Hasegawa T, Saito S.<br>J Obstet Gynaecol Res. 2011 Dec;37(12):1883-6.
Sands - Sullivan, 2015	Focal seizures in children with anti-NMDA receptor antibody encephalitis.<br>Sands TT, Nash K, Tong S, Sullivan J.<br>Epilepsy Res. 2015 May;112:31-6.
Sanmaneechai – Overby, 2013	Anti-N-methyl-d-aspartate encephalitis with ovarian cystadenofibroma.<br>Sanmaneechai O, Song JL, Nevadunsky N, Moshé SL, Overby PJ.<br>Pediatr Neurol. 2013 Mar;48(3):232-5.
Sansing - Dalmau, 2007	A patient with encephalitis associated with NMDA receptor antibodies.<br>Sansing LH, Tüzün E, Ko MW, Baccon J, Lynch DR, Dalmau J.<br>Nat Clin Pract Neurol. 2007 May;3(5):291-6.
Sarigecili - Okuyaz, 2018	A rare concurrence: Antibodies against Myelin Oligodendrocyte Glycoprotein and N-methyl-d-aspartate receptor in a child.<br>Sarigecili E, Cobanogullari MD, Komur M, Okuyaz C.<br>Mult Scler Relat Disord. 2018

© 2021 Nosadini M et al. *JAMA Neurology*.

Sarkis - Chemali, 2014	Neuropsychiatric and seizure outcomes in nonparaneoplastic autoimmune limbic encephalitis.<br>Sarkis RA, Nehme R, Chemali ZN.<br>Epilepsy Behav. 2014 Oct;39:21-5
Sato - Arai, 2018	General anesthesia with propofol for ovarian teratoma excision associated with anti-N-methyl-D-aspartate receptor encephalitis.<br>Sato M, Yasumoto H, Arai T.<br>JA Clin Rep. 2018;4(1):14.
Sawamura - Tsuji, 2014	Anti-NMDA receptor encephalitis associated with transient cerebral dyschromatopsia, prosopagnosia, and lack of stereopsis.<br>Sawamura H, Yamamoto T, Ohtomo R, Bannai T, Wakakura M, Tsuji S.<br>J Neuroophthalmol. 2014 Jun;34(2):144-8.
Schankin - Straube, 2016	New-Onset Headache in Patients With Autoimmune Encephalitis Is Associated With anti-NMDA-Receptor Antibodies.<br>Schankin CJ, Kästele F, Gerdes LA, Winkler T, Csanadi E, Högen T, Pellkofer H, Paulus W, Kümpfel T, Straube A.<br>Headache. 2016 Jun;56(6):995-1003
Scheibe - Meise, 2017	Bortezomib for treatment of therapy-refractory anti-NMDA receptor encephalitis.<br>Scheibe F, Prüss H, Mengel AM, Kohler S, Nümann A, Köhnlein M, Ruprecht K, Alexander T, Hiepe F, Meisel A.<br>Neurology. 2017 Jan 24;88(4):366-370.
Schimmel – Penzien, 2009	Successful treatment of anti-N-methyl-D-aspartate receptor encephalitis presenting with catatonia.<br>Schimmel M, Bien CG, Vincent A, Schenk W, Penzien J.<br>Arch Dis Child. 2009 Apr;94(4):314-6.
Schmiedeskamp - Ranta, 2010	Anti-NMDA-receptor autoimmune encephalitis without neoplasm: a rare condition?<br>Schmiedeskamp M, Cariga P, Ranta A.<br>N Z Med J. 2010 Sep 10;123(1322):67-71.
Schroeder - Haghikia, 2018	Breakthrough treatment with bortezomib for a patient with anti-NMDAR encephalitis.<br>Schroeder C, Back C, Koc Ü, Strassburger-Krogias K, Reinacher-Schick A, Gold R, Haghikia A.<br>Clin Neurol Neurosurg. 2018 Sep;172:24-26.
Schumacher - MacKenzie, 2016	Agitation Management in Pediatric Males with Anti-N-Methyl-D-Aspartate Receptor Encephalitis.<br>Schumacher LT, Mann AP, MacKenzie JG.<br>J Child Adolesc Psychopharmacol. 2016 Dec;26(10):939-943.
Scott – Goez, 2014	Anti-N-methyl-D-aspartate (NMDA) receptor encephalitis: an unusual cause of autistic regression in a toddler.<br>Scott O, Richer L, Forbes K, Sonnenberg L, Currie A, Eliyashevska M, Goez HR.<br>J Child Neurol. 2014 May;29(5):691-4.
See – Crawford, 2012	Acute encephalitis secondary to an ovarian teratoma.<br>See AT, Woo YL, Crawford R.<br>J Obstet Gynaecol. 2012 Aug;32(6):604-6.
Seifi - Felte, 2013	Thinking outside the box about young female patients with sudden-onset bizarre behavior: a case of anti-N-methyl-D-aspartate receptor encephalitis.<br>Seifi A, Xia BT, Felte RF.<br>Prim Care Companion CNS Disord. 2013;15(4). pii: PCC.13l01521
Seifi - Kitchen, 2016	Management of dyskinesia in anti-NMDAR encephalitis with tramadol.<br>Seifi A, Kitchen DL.<br>Clin Neurol Neurosurg. 2016 Aug;147:105-7
Seki – Dalmau, 2008	Neurological response to early removal of ovarian teratoma in anti-NMDAR encephalitis.<br>Seki M, Suzuki S, Iizuka T, Shimizu T, Nihei Y, Suzuki N, Dalmau J.<br>J Neurol Neurosurg Psychiatry. 2008 Mar;79(3):324-6. Epub 2007 Nov 21.
Senbruna - Lerman, 2015	Anesthesia Management for a Boy with Anti-N-Methyl-D-Aspartate Receptor Encephalitis.<br>Senbruna B, Lerman J.<br>A A Case Rep. 2015 Nov 15;5(10):182-4.
Servais - Hantson, 2017	Movement disorders in anti-NMDA receptor encephalitis.<br>Servais E, Fastre S, Hantson P.<br>Neurol India. 2017 May-Jun;65(3):632-633
Seward, 2018	Anti-N-methyl-D-aspartate receptor antibody encephalitis: An important cause of encephalitis in young adults. A report of two cases.<br>Seward S.<br>J Am Coll Health. 2018 Jan 31:1-3.
Shaaban – Sensakovic, 2012	Anti-NMDA-receptor encephalitis presenting as postpartum psychosis in a young woman, treated with rituximab.<br>Shaaban HS, Choo HF, Sensakovic JW.<br>Ann Saudi Med. 2012 Jul-Aug;32(4):421-3.
Shahani , 2015	Steroid unresponsive anti-NMDA receptor encephalitis during pregnancy successfully treated with plasmapheresis.<br>Shahani L.<br>BMJ Case Rep. 2015 Apr 29;2015. pii: bcr2014208823.
Shaikh - Shaikh, 2015	Anaesthetic management of a patient with anti-NMDA receptor encephalitis.<br>Shaikh MA, Dhansura T, Gandhi S, Shaikh T.<br>Indian J Anaesth. 2015 Apr;59(4):248-50
Sharma - Gupta, 2014	Anti-NMDA receptor encephalitis: a neurological disease in psychiatric disguise.<br>Sharma B, Handa R, Prakash S, Nagpal K, Gupta P.<br>Asian J Psychiatr. 2014 Feb;7(1):92-4.

© 2021 Nosadini M et al. *JAMA Neurology*.

Sharma - Chakrabarty, 2016	Psychotic symptoms in anti-N-methyl-d-aspartate (NMDA) receptor encephalitis: A case report and challenges.<br>Sharma P, Sagar R, Patra B, Saini L, Gulati S, Chakrabarty B.<br>Asian J Psychiatr. 2016 Aug;22:135-7.
Shi, 2017	Serial EEG Monitoring in a Patient With Anti-NMDA Receptor Encephalitis.<br>Shi Y.<br>Clin EEG Neurosci. 2017 Jul;48(4):301-303.
Shimazaki – Dalmau, 2008	Inverse ocular bobbing in a patient with encephalitis associated with antibodies to the N-methyl-D-aspartate receptor.<br>Shimazaki H, Morita M, Nakano I, Dalmau J.<br>Arch Neurol. 2008 Sep;65(9):1251.
Shimoyama - Minami, 2016	Anti-NMDA receptor encephalitis presenting as an acute psychotic episode misdiagnosed as dissociative disorder: a case report.<br>Shimoyama Y, Umegaki O, Agui T, Kadono N, Minami T.<br>JA Clin Rep. 2016;2(1):22.
Shin - Chu, 2018	Bortezomib treatment for severe refractory anti-NMDA receptor encephalitis.<br>Shin YW, Lee ST, Kim TJ, Jun JS, Chu K.<br>Ann Clin Transl Neurol. 2018 Mar 23;5(5):598-605.
Shindo - Kuzuhara, 2009	Anti-N-methyl-D-aspartate receptor-related grave but reversible encephalitis with ovarian teratoma in 2 Japanese women presenting with excellent recovery without tumor resection.<br>Shindo A, Kagawa K, Ii Y, Sasaki R, Kokubo Y, Kuzuhara S.<br>Eur Neurol. 2009;61(1):50-1.
Shruthi - Chitrambalam, 2014	Anti-NMDA receptor encephalitis in an adolescent.<br>Shruthi TK, Shuba S, Rajakumar PS, Chitrambalam S.<br>Indian Pediatr. 2014 May;51(5):405-6.
Sieg - VanHaerents, 2018	Neuropsychological assessment as an objective tool to monitor treatment response in anti-N-methyl-D-aspartate receptor encephalitis.<br>Sieg E, Brook M, Linnoila J, VanHaerents S.<br>BMJ Case Rep. 2018 Jun 8;2018.
Simabukuro - Anghinah, 2014	A successful case of anti-NMDAR encephalitis without tumor treated with a prolonged regimen of plasmapheresis.<br>Simabukuro MM, Watanabe RGS, Pinto LF, Guariglia C, Gonçalves DCME, Anghinah R.<br>Dement Neuropsychol. 2014 Jan-Mar;8(1):87-89.
Simabukuro - Castro, 2015	A patient with a long history of relapsing psychosis and mania presenting with anti-NMDA receptor encephalitis ten years after first episode.<br>Simabukuro MM, Freitas CHA, Castro LHM.<br>Dement Neuropsychol. 2015 Jul-Sep;9(3):311-314.
Simon, 2014	Anesthetic management and implications of pediatric patients with a diagnosis of anti-N-methyl-D-aspartate receptor encephalitis: two case reports.<br>Simon RW.<br>AANA J. 2014 Dec;82(6):431-6.
Singh - Prabhakar, 2016	Neuropsychiatric Presentation of Anti-N-Methyl-D-Aspartate Receptor Encephalitis With Comorbid Sinus Venous Thrombosis.<br>Singh G, Prabhakar D.<br>Prim Care Companion CNS Disord. 2016 Dec 1;18(6).
Slettedal – Strømme, 2012	Young girl with psychosis, cognitive failure and seizures.<br>Slettedal IÖ, Dahl HM, Sandvig I, Dalmau J, Strømme P.<br>Tidsskr Nor Laegeforen. 2012 Oct 2;132(18):2073-6.
Smith – Kantarci, 2011	N-methyl-D-aspartate receptor autoimmune encephalitis presenting with opsoclonus-myoclonus: treatment response to plasmapheresis.<br>Smith JH, Dhamija R, Moseley BD, Sandroni P, Lucchinetti CF, Lennon VA, Kantarci OH.<br>Arch Neurol. 2011 Aug;68(8):1069-72.
Soares – Brucki, 2013	Anti-NMDA-R encephalitis: follow-up of 24 months.<br>Soares EMV, Kauark RBG, Rocha MSG, Brucki SMD.<br>Dement Neuropsychol. 2013 Jul-Sep;7(3):304-307.
Soares - Poretti, 2017	MR Imaging of Acute Cerebellar Involvement in Pediatric Anti-N-Methyl-D-Aspartate Receptor Encephalitis.<br>Soares BP, Calloni SF, Shrot S, Poretti A.<br>Neuropediatrics. 2017 Oct;48(5):398-399.
Solís - Hasbun, 2016	Anti-NMDA Receptor antibody encephalitis with concomitant detection of Varicella zoster virus.<br>Solís N, Salazar L, Hasbun R.<br>J Clin Virol. 2016 Oct;83:26-8.
Sommeling - Santens, 2014	Anti-N-methyl-D-aspartate (anti-NMDA) receptor antibody encephalitis in a male adolescent with a large mediastinal teratoma.<br>Sommeling C, Santens P.<br>J Child Neurol. 2014 May;29(5):688-90.
Sonn – Merritt, 2010	Anti-NMDA-receptor encephalitis: an adolescent with an ovarian teratoma.<br>Sonn TS, Merritt DF.<br>J Pediatr Adolesc Gynecol. 2010 Oct;23(5):e141-4.
Söylemez - Dayan, 2015	Extreme delta brush EEG pattern in a case with anti-NMDA receptor encephalitis.<br>Söylemez E, Güveli BT, Atakli D, Yatmazoğlu M, Atay T, Dayan C.<br>Ideggyogy Sz. 2015 Sep 30;68(9-10):357-60.

© 2021 Nosadini M et al. *JAMA Neurology*.

Spatola - Dalmau, 2017	Investigations in GABAA receptor antibody-associated encephalitis.<br>Spatola M, Petit-Pedrol M, Simabukuro MM, Armangue T, Castro FJ, Barcelo Artigues MI, Julià Benique MR, Benson L, Gorman M, Felipe A, Caparó Oblitas RL, Rosenfeld MR, Graus F, Dalmau J.<br>Neurology. 2017 Mar 14;88(11):1012-1020.
Splendiani - Massimo, 2016	Magnetic resonance imaging and magnetic resonance spectroscopy in a young male patient with anti-N-methyl-D-aspartate receptor encephalitis and uncommon cerebellar involvement: A case report with review of the literature.<br>Splendiani A, Felli V, Di Sibio A, Gennarelli A, Patriarca L, Stratta P, Di Cesare E, Rossi A, Massimo G.<br>Neuroradiol J. 2016 Feb;29(1):30-5.
Splinter - Eipe, 2009	Anti-NMDA receptor antibodies encephalitis.<br>Splinter WM, Eipe N.<br>Paediatr Anaesth. 2009 Sep;19(9):911-3.
Steriade - Rae-Grant, 2018	Extreme delta - With or without brushes: A potential surrogate marker of disease activity in anti-NMDA-receptor encephalitis.<br>Steriade C, Hantus S, Moosa ANV, Rae-Grant AD.<br>Clin Neurophysiol. 2018 Oct;129(10):2197-2204.
Stover – Johnson, 2010	Anti-N-methyl-D-aspartate receptor encephalitis in a young woman with a mature mediastinal teratoma.<br>Stover DG, Eisenberg R, Johnson DH.<br>J Thorac Oncol. 2010 Nov;5(11):1872-3.
Suárez - Casado, 2016	Brain perfusion SPECT with 99mTc-HMPAO in the diagnosis and follow-up of patients with anti-NMDA receptor encephalitis.<br>Suárez JP, Domínguez ML, Gómez MA, Portilla JC, Gómez M, Casado I.<br>Neurologia. 2016 Jul 21.
Sudan - Patil, 2016	Clinical Characteristics and Follow-up of South Indian Children with Autoimmune Encephalopathy.<br>Sudan YS, Vinayan KP, Roy AG, Wagh A, Kannoth S, Patil S.<br>Indian J Pediatr. 2016 Nov;83(12-13):1367-1373.
Sühs - Stangel, 2015	Heterogeneity of clinical features and corresponding antibodies in seven patients with anti-NMDA receptor encephalitis.<br>Sühs KW, Wegner F, Skripuletz T, Trebst C, Tayeb SB, Raab P, Stangel M.<br>Exp Ther Med. 2015 Oct;10(4):1283-1292.
Suleman - Javed, 2018	NMDAR (N-methyl-D-aspartate receptor) encephalitis in a patient with MS (multiple sclerosis): a rare and challenging case.<br>Suleman S, Javed Q.<br>BMJ Case Rep. 2018 Feb 17;2018.
Sulentic – Nankovic, 2018	The effect of delayed anti-NMDAR encephalitis recognition on disease outcome.<br>Sulentic V, Petelin Gadze Z, Derke F, Santini M, Bazadona D, Nankovic S.<br>J Neurovirol. 2018 May 21.
Sunwoo - Chu, 2016	Successful Treatment of Refractory Dyskinesia Secondary to Anti-N-Methyl-D-Aspartate Receptor Encephalitis With Electroconvulsive Therapy.<br>Sunwoo JS, Jung DC, Choi JY, Kang UG, Lee ST, Lee SK, Chu K.<br>J ECT. 2016 Sep;32(3):e13-4.
Suri-Jadhao – Jadhao, 2013	Pediatric anti-N methyl D aspartate receptor encephalitis.<br>Suri V, Sharma S, Gupta R, Sogani SK, Mediratta S, Jadhao N.<br>J Pediatr Neurosci. 2013 May;8(2):120-2.
Suri – Suri, 2013	Young girl with abnormal behavior: Anti-N-Methyl-D-Aspartate receptor immune encephalitis.<br>Suri V, Sharma S, Gupta R, Jadhao N, Suri K.<br>Ann Indian Acad Neurol. 2013 Apr;16(2):169-71.
Suthar - Singhi, 2016	Hyperkinetic Movement Disorder in a Girl with Anti-NMDA Receptor Encephalitis.<br>Suthar R, Sankhyan N, Singhi P.<br>Indian Pediatr. 2016 Jan;53(1):81.
Suzuki – Koyama, 2009	[Case report of anti-NMDA receptor encephalitis suspected of schizophrenia].<br>Suzuki Y, Kurita T, Sakurai K, Takeda Y, Koyama T.<br>Seishin Shinkeigaku Zasshi. 2009;111(12):1479-84.
Suzuki – Kusunoki, 2013	Anti-NMDAR encephalitis preceded by dura mater lesions.<br>Suzuki H, Kitada M, Ueno S, Tanaka K, Kusunoki S.<br>Neurol Sci. 2013 Jun;34(6):1021-2.
Suzuki – Kusunoki, 2011	A case of anti-N-methyl-D-aspartate receptor encephalitis with systemic sclerosis.<br>Suzuki H, Samukawa M, Kitada M, Ichihashi J, Mistui Y, Tanaka K, Kusunoki S.<br>Eur J Neurol. 2011 Nov;18(11):e145-6.
Sveinsson - Piehl, 2017	Successful combined targeting of B- and plasma cells in treatment refractory anti-NMDAR encephalitis.<br>Sveinsson O, Granqvist M, Forslin Y, Blennow K, Zetterberg H, Piehl F.<br>J Neuroimmunol. 2017 Nov 15;312:15-18. doi: 10.1016/j.jneuroim.2017.08.011.
Tabata - Hara, 2014	Immunopathological significance of ovarian teratoma in patients with anti-N-methyl-d-aspartate receptor encephalitis.<br>Tabata E, Masuda M, Eriguchi M, Yokoyama M, Takahashi Y, Tanaka K, Yukitake M, Horikawa E, Hara H.<br>Eur Neurol. 2014;71(1-2):42-8.
Tachibana – Ikeda, 2010	Expression of various glutamate receptors including N-methyl-D-aspartate receptor (NMDAR) in an ovarian teratoma removed from a young woman with anti-NMDAR encephalitis.<br>Tachibana N, Shirakawa T, Ishii K, Takahashi Y, Tanaka K, Arima K, Yoshida T, Ikeda S.<br>Intern Med. 2010;49(19):2167-73.

© 2021 Nosadini M et al. *JAMA Neurology*.

Taguchi - Tanaka, 2011	[A case of anti-N-methyl-D-aspartate receptor encephalitis with ovarian teratoma showing excellent recovery with decreasing of anti-N-methyl-D-aspartate receptor antibody].<br>Taguchi Y, Takashima S, Takano S, Mori H, Tanaka K.<br>Rinsho Shinkeigaku. 2011 Jul;51(7):499-504. Japanese.
Taguchi - Tanaka, 2011	Reversible "brain atrophy" in anti-NMDA receptor encephalitis.<br>Taguchi Y, Takashima S, Nukui T, Tanaka K.<br>Intern Med. 2011;50(21):2697. Epub 2011 Nov 1. No abstract available.
Taguchi - Tanaka, 2010	Hypersalivation in a patient with anti-NMDAR encephalitis with ovarian teratoma.<br>Taguchi Y, Takashima S, Nukui T, Tanaka K.<br>Intern Med. 2010;49(8):803-4. Epub 2010 Apr 15.
Takeda - Miki, 2014	A case of anti-N-methyl-d-aspartate receptor encephalitis with multiple sclerosis-like demyelinated lesions.<br>Takeda A, Shimada H, Tamura A, Yasui M, Yamamoto K, Itoh K, Ataka S, Tanaka S, Ohsawa M, Hatsuta H, Hirano M, Sakamoto H, Ueno S, Nakamura Y, Tsutada T, Miki T.<br>Mult Scler Relat Disord. 2014 May;3(3):391-7.
Takeshita – Takeshita, 2011	A case of paraneoplastic limbic encephalitis associated with ovarian teratoma and N-Methyl-d-Aspartate receptor antibodies.<br>Takeshita L, Domnitei D, Takeshita J.<br>Prim Care Companion CNS Disord. 2011;13(5)
Tamma – Hartman, 2011	Behavior outbursts, orofacial dyskinesias, and CSF pleocytosis in a healthy child.<br>Tamma PD, Agwu AL, Hartman AL.<br>Pediatrics. 2011 Jul;128(1):e242-5.
Tan, 2010	A modern perspective on the differential diagnosis between encephalitis lethargica or anti-NMDA-receptor encephalitis.<br>Tan A, Shuey N, Bladin C.<br>J Clin Neurosci. 2010 Sep;17(9):1204-6.
Tang - Tan, 2009	A multimodality approach to reversible paraneoplastic encephalitis associated with ovarian teratomas.<br>Tang T, Tay KY, Chai J, Agarwal A, Low J, Lee EL, Chuah KL, Sittampalan KS, Wong CF, Dalmau J, Ngeow J, Soh LT, Tan MH.<br>Acta Oncol. 2009;48(7):1079-82.
Tantipalakorn - Tongsong, 2016	Rapid recovery from catastrophic paraneoplastic anti-NMDAR encephalitis secondary to an ovarian teratoma following ovarian cystectomy.<br>Tantipalakorn C, Soontornpun A, Pongsuvareeyakul T, Tongsong T.<br>BMJ Case Rep. 2016 Aug 10;2016.
Tanyi - Chu, 2012	Reversible paraneoplastic encephalitis in three patients with ovarian neoplasms.<br>Tanyi JL, Marsh EB, Dalmau J, Chu CS.<br>Acta Obstet Gynecol Scand. 2012 May;91(5):630-4.
Tapin - Merlin, 2013	[Anti-NMDAR paraneoplastic encephalitis].<br>Tapin M, Faucher R, Poirier V, Curinier S, Merlin E.<br>Arch Pediatr. 2013 Nov;20(11):1255-6.
Taraschenko - McKee, 2014	Anti-NMDA receptor encephalitis associated with atrial fibrillation and hearing loss.<br>Taraschenko O, Zimmerman EA, Bunch ME, McKee MA.<br>Neurol Neuroimmunol Neuroinflamm. 2014 Aug 21;1(3):e24.
Tarula - Sheen, 2012	Progressive mental status changes and seizures with fluid attenuated inversion recovery (FLAIR) hyperintensity on brain MRI.<br>Tarula E, Ramkissoon S, Pittock S, Sheen VL.<br>J Clin Neurosci. 2012 Jun;19(6):873, 924.
Tatencloux - Deiva, 2015	Intrathecal treatment of anti-N-Methyl-D-aspartate receptor encephalitis in children.<br>Tatencloux S, Chretien P, Rogemond V, Honnorat J, Tardieu M, Deiva K.<br>Dev Med Child Neurol. 2015 Jan;57(1):95-9.
Terada - Murakami, 2017	Two cases of acute limbic encephalitis in which symptoms improved as a result of laparoscopic salpingo-oophorectomy.<br>Terada A, Tasaki S, Tachibana T, Sakamoto Y, Yokomine M, Shimomura T, Murakami F.<br>Gynecol Minim Invasive Ther. 2017 Jan-Mar;6(1):34-37.
Thilagavathi - Cheong , 2013	Anti NMDA Receptor Encephalitis: A Differential Diagnosis in a Young Patient Presenting with Neuropsychiatric Symptoms.<br>Thilagavathi TV, Cheong B.<br>Med J Malaysia. 2013 Oct;68(5):437-8.
Thomas – Fadul, 2013	Anti-N-methyl-D-aspartate receptor encephalitis: a patient with refractory illness after 25 months of intensive immunotherapy.<br>Thomas A, Rauschkolb P, Gresa-Arribas N, Schned A, Dalmau JO, Fadul CE.<br>JAMA Neurol. 2013 Dec;70(12):1566-8.
Thomas - Honnorat, 2014	Autoimmune N-methyl-D-aspartate receptor encephalitis is a differential diagnosis of infectious encephalitis.<br>Thomas L, Mailles A, Desestret V, Ducray F, Mathias E, Rogemond V, Didelot A, Marignier S, Stahl JP, Honnorat J; Steering Committee and Investigators Group.<br>J Infect. 2014 May;68(5):419-25.
Tidswell – Galletly, 2013	Early recognition of anti-N-methyl D-aspartate (NMDA) receptor encephalitis presenting as acute psychosis.<br>Tidswell J, Kleinig T, Ash D, Thompson P, Galletly C.<br>Australas Psychiatry. 2013 Dec;21(6):596-9.
Titulaer, McCracken - Dalmau, 2013	Late-onset anti-NMDA receptor encephalitis.<br>Titulaer MJ, McCracken L, Gabilondo I, Iizuka T, Kawachi I, Bataller L, Torrents A, Rosenfeld MR, Balice-

© 2021 Nosadini M et al. *JAMA Neurology*.

	Gordon R, Graus F, Dalmau J.<br>Neurology. 2013 Sep 17;81(12):1058-63.
Tituler, Höftberger - Dalmau, 2014	Overlapping demyelinating syndromes and anti–N-methyl-D-aspartate receptor encephalitis.<br>Titulaer MJ, Höftberger R, Iizuka T, Leypoldt F, McCracken L, Cellucci T, Benson LA, Shu H, Irioka T, Hirano M, Singh G, Cobo Calvo A, Kaida K, Morales PS, Wirtz PW, Yamamoto T, Reindl M, Rosenfeld MR, Graus F, Saiz A, Dalmau J.<br>Ann Neurol. 2014 Mar;75(3):411-28.
Tobin - Pittock, 2014	NMDA receptor encephalitis causing reversible caudate changes on MRI and PET imaging.<br>Tobin WO, Strand EA, Clark HM, Lowe VJ, Robertson CE, Pittock SJ.<br>Neurol Clin Pract. 2014 Dec;4(6):470-473.
Tojo – Ikeda, 2011	A Young Man with Anti-NMDAR Encephalitis following Guillain-Barré Syndrome.<br>Tojo K, Nitta K, Ishii W, Sekijima Y, Morita H, Takahashi Y, Tanaka K, Ikeda S.<br>Case Rep Neurol. 2011 Jan 11;3(1):7-13.
Tokunaga - Kato, 2018	Transient extreme spindles in a young child with anti-NMDAR encephalitis: A case report.<br>Tokunaga S, Ide M, Ishihara T, Matsumoto T, Maihara T, Kato T.<br>Brain Dev. 2018 Sep 29. pii: S0387-7604(18)30093-7. doi: 10.1016/j.braindev.2018.09.001. [Epub ahead of print]
Tonomura, Kataoka – Ueno, 2007	Clinical analysis of paraneoplastic encephalitis associated with ovarian teratoma.<br>Tonomura Y, Kataoka H, Hara Y, Takamure M, Naba I, Kitauti T, Saito K, Ueno S.<br>J Neurooncol. 2007 Sep;84(3):287-92. Epub 2007 Apr 13.<br><br>Paraneoplastic encephalitis associated with ovarian teratoma and N-methyl-D-aspartate receptor antibodies.<br>Kataoka H, Dalmau J, Ueno S.<br>Eur J Neurol. 2008 Jan;15(1):e5-6. Epub 2007 Nov 27.<br><br>Reduced N-acetylaspartate in the basal ganglia of a patient with anti-NMDA receptor encephalitis.<br>Kataoka H, Dalmau J, Taoka T, Ueno S.<br>Mov Disord. 2009 Apr 15;24(5):784-6.
Toral - Dumas, 2018	Valproate delays diagnosis of anti-NMDA-receptor-encephalitis in a patient with psychiatric presentation.<br>Toral D, Belzeaux R, Dumas R.<br>Aust N Z J Psychiatry. 2018 Dec 19:4867418818752.
Tsutsui - Nishino, 2012	Anti-NMDA-receptor antibody detected in encephalitis, schizophrenia, and narcolepsy with psychotic features.<br>Tsutsui K, Kanbayashi T, Tanaka K, Boku S, Ito W, Tokunaga J, Mori A, Hishikawa Y, Shimizu T, Nishino S.<br>BMC Psychiatry. 2012 May 8;12:37.
Tsutsui - Shimizu, 2017	N-Methyl-D-aspartate receptor antibody could be a cause of catatonic symptoms in psychiatric patients: case reports and methods for detection.<br>Tsutsui K, Kanbayashi T, Takaki M, Omori Y, Imai Y, Nishino S, Tanaka K, Shimizu T.<br>Neuropsychiatr Dis Treat. 2017 Feb 8;13:339-345.
Tsuyusaki – Yoshida, 2014	Downbeat nystagmus as the initial manifestation of anti-NMDAR encephalitis.<br>Tsuyusaki Y, Sakakibara R, Kishi M, Tateno F, Yoshida T.<br>Neurol Sci. 2014 Jan;35(1):125-6.
Turkdoğan -Ekinci, 2014	Anti-N-methyl-d-aspartate (Anti-NMDA) receptor encephalitis: rapid and sustained clinical improvement with steroid therapy starting in the late phase.<br>Turkdoğan D, Orengul AC, Zaimoğlu S, Ekinci G.<br>J Child Neurol. 2014 May;29(5):684-7.
Tüzün - Akman-Demir, 2013	Anti-N-methyl-D-aspartate receptor encephalitis with minimal cortical impairment.<br>Tüzün E, Türkoğlu R, Yumerhodzha SM, Erdağ E, Eraksoy M, Akman-Demir G.<br>Neurol Sci. 2013 Jan;34(1):111-3.
Tzang – Chen, 2018	Immune modulating therapy: An effective add-on intervention for psychosis of anti-N-methyl-d-aspartate receptor encephalitis in Taiwan.<br>Tzang RF, Chang CH, Chang YC, Hsu K, Chen CC.<br>Psychiatry Clin Neurosci. 2018 May 24.
Uchida - Matsukawa, 2017	Combination of ketogenic diet and stiripentol for super-refractory status epilepticus: A case report.<br>Uchida Y, Kato D, Toyoda T, Oomura M, Ueki Y, Ohkita K, Matsukawa N.<br>J Neurol Sci. 2017 Feb 15;373:35-37.
Uchida - Matsukawa, 2018	Failure to improve after ovarian resection could be a marker of recurrent ovarian teratoma in anti-NMDAR encephalitis: a case report.<br>Uchida Y, Kato D, Yamashita Y, Ozaki Y, Matsukawa N.<br>Neuropsychiatr Dis Treat. 2018 Jan 22;14:339-342.
Uchino – Mochizuki, 2011	Pseudo-piano playing motions and nocturnal hypoventilation in anti-NMDA receptor encephalitis: response to prompt tumor removal and immunotherapy.<br>Uchino A, Iizuka T, Urano Y, Arai M, Hara A, Hamada J, Hirose R, Dalmau J, Mochizuki H.<br>Intern Med. 2011;50(6):627-30.
Ueda - Kohara, 2017	Serial EEG findings in anti-NMDA receptor encephalitis: correlation between clinical course and EEG.<br>Ueda J, Kawamoto M, Hikiami R, Ishii J, Yoshimura H, Matsumoto R, Kohara N.<br>Epileptic Disord. 2017 Dec 1;19(4):465-470.
Ueda - Mutoh, 2017	Absence of serum anti-NMDAR antibodies in anti-NMDAR encephalitis mother predicts having healthy newborn.<br>Ueda A, Nagao R, Maeda T, Kikuchi K, Murate K, Niimi Y, Shima S, Mutoh T.<br>Clin Neurol Neurosurg. 2017 Oct;161:14-16.

© 2021 Nosadini M et al. *JAMA Neurology*.

Urriola - Halmágyi, 2018	NMDA receptor antibody in teratoma-related opsoclonus-myoclonus syndrome.<br>Urriola NX, Helou J, Maamary J, Pogson J, Lee F, Parratt K, Gillis D, Fulham MJ, Halmágyi GM.<br>J Clin Neurosci. 2018 Oct 16. pii: S0967-5868(18)30739-2.
Uruha – Koide, 2012	Anti-NMDAR encephalitis in small-cell lung cancer: a case report.<br>Uruha A, Kitazawa Y, Kuroda M, Tanaka K, Koide R.<br>Clin Neurol Neurosurg. 2012 Apr;114(3):260-1.
Uzawa - Kuwabara, 2012	Anti-N-methyl D-aspartate-type glutamate receptor antibody-positive limbic encephalitis in a patient with multiple sclerosis.<br>Uzawa A, Mori M, Takahashi Y, Ogawa Y, Uchiyama T, Kuwabara S.<br>Clin Neurol Neurosurg. 2012 May;114(4):402-4.
Vahter - Gross-Paju, 2014	Cognitive dysfunction during anti-NMDA-receptor encephalitis is present in early phase of the disease.<br>Vahter L, Kannel K, Sorro U, Jaakmees H, Talvik T, Gross-Paju K.<br>Oxf Med Case Reports. 2014 Jul 12;2014(4):74-6.
van de Riet - Schieveld, 2013	Anti-NMDAR encephalitis: a new, severe and challenging enduring entity.<br>van de Riet EH, Esseveld MM, Cuypers L, Schieveld JN.<br>Eur Child Adolesc Psychiatry. 2013 May;22(5):319-23
van der Meulen - Elting, 2017	Extreme delta brushes in anti NMDA receptor encephalitis - Muscle artefact or an EEG phenomenon? A case report.<br>van der Meulen AAE, van der Hoeven JH, de Jong BM, Elting JWJ.<br>Clin Neurophysiol. 2017 Oct;128(10):1835-1836.
Van Putten – Huyghens, 2012	Uncommon cause of psychotic behavior in a 9-year-old girl: a case report.<br>Van Putten WK, Hachimi-Idrissi S, Jansen A, Van Gorp V, Huyghens L.<br>Case Rep Med. 2012;2012:358520.
van Vliet – Mulleners, 2012	[Autoimmune limbic encephalitis: importance of early diagnosis and treatment].<br>van Vliet J, Meulstee J, Hartong EG, Mulleners WM.<br>Ned Tijdschr Geneeskd. 2012;156(16):A4455. Dutch.
VanHaerents - Herman, 2014	Early and persistent 'extreme delta brush' in a patient with anti-NMDA receptor encephalitis.<br>VanHaerents S, Stillman A, Inoa V, Searls DE, Herman ST.<br>Epilepsy Behav Case Rep. 2014 Feb 12;2:67-70.
Vanya - Bártfai, 2016	NMDA-receptor associated encephalitis in a woman with mature cystic ovarian teratoma.<br>Vanya M, Füvesi J, Kovács ZA, Gorgoraptis N, Salek-Haddadi A, Kovács L, Bártfai G.<br>Ideggyogy Sz. 2016 Nov 30;69(11-12):427-432.
Vargas - Bromley, 2016	Ovarian Teratomas and Anti-N-Methyl-d-Aspartate Receptor Encephalitis: Why Sonography First?<br>Vargas RJ, Farid H, Goldenson RP, Fairchild AH, Dorton BJ, Bromley BS.<br>J Ultrasound Med. 2016 Apr;35(4):852-4.
Venâncio - Vieira, 2014	Anti-N-methyl-D-aspartate receptor encephalitis with positive serum antithyroid antibodies, IgM antibodies against mycoplasma pneumoniae and human herpesvirus 7 PCR in the CSF.<br>Venâncio P, Brito MJ, Pereira G, Vieira JP.<br>Pediatr Infect Dis J. 2014 Aug;33(8):882-3.
Verfaille - Spapen, 2013	An unusual case of acute psychosis in an adolescent.<br>Verfaillie L, Bissay V, Vanderbruggen N, Van Eetvelde E, Honoré PM, Spapen H.<br>Acta Clin Belg. 2013 Mar-Apr;68(2):138-9. Review.
Verhelst – Van Coster, 2011	Anti-NMDA-receptor encephalitis in a 3 year old patient with chromosome 6p21.32 microdeletion including the HLA cluster.<br>Verhelst H, Verloo P, Dhondt K, De Paepe B, Menten B, Dalmau J, Van Coster R.<br>Eur J Paediatr Neurol. 2011 Mar;15(2):163-6.
Viaccoz - Honnorat, 2014	Clinical specificities of adult male patients with NMDA receptor antibodies encephalitis.<br>Viaccoz A, Desestret V, Ducray F, Picard G, Cavillon G, Rogemond V, Antoine JC, Delattre JY, Honnorat J.<br>Neurology. 2014 Feb 18;82(7):556-63.
Voice - Lakhi, 2017	Psychosis secondary to an incidental teratoma: a "heads-up" for psychiatrists and gynecologists.<br>Voice J, Ponterio JM, Lakhi N.<br>Arch Womens Ment Health. 2017 Oct;20(5):703-707.
Volz – Prüss, 2016	Altered paired associative stimulation-induced plasticity in NMDAR encephalitis.<br>Volz MS, Finke C, Harms L, Jurek B, Paul F, Flöel A, Prüss H.<br>Ann Clin Transl Neurol. 2016 Jan 16;3(2):101-13.
Vural – Topcuoglu, 2012	Central neurogenic hyperventilation in anti-NMDA receptor encephalitis.<br>Vural A, Arsava EM, Dericioglu N, Topcuoglu MA.<br>Intern Med. 2012;51(19):2789-92.
Waas – Storm, 2012	[Anti- NMDA- receptor encephalitis; a neuropsychiatric illness requiring further study].<br>Waas JA, Storm AH.<br>Tijdschr Psychiatr. 2012;54(3):279-83. Dutch.
Wada - Yorozu, 2018	Anesthesia for patient with anti-N-methyl-D-aspartate receptor encephalitis: A case report with a brief review of the literature.<br>Wada N, Tashima K, Motoyasu A, Nakazawa H, Tokumine J, Chinzei M, Yorozu T.<br>Medicine (Baltimore). 2018 Dec;97(50):e13651.
Wali – Clough, 2011	Appearance of anti-NMDAR antibodies after plasma exchange and total removal of malignant ovarian teratoma in a patient with paraneoplastic limbic encephalopathy.<br>Wali SM, Cai A, Rossor AM, Clough C.<br>BMJ Case Rep. 2011 Mar 24;2011

© 2021 Nosadini M et al. *JAMA Neurology*.

Wang - Guo, 2017	Lower dosages of rituximab used successfully in the treatment of anti-NMDA receptor encephalitis without tumour.<br>Wang BJ, Wang CJ, Zeng ZL, Yang Y, Guo SG.<br>J Neurol Sci. 2017 Jun 15;377:127-132.
Wang - Luo, 2015	Extreme delta brush guides to the diagnosis of anti-NMDAR encephalitis.<br>Wang J, Wang K, Wu D, Liang H, Zheng X, Luo B.<br>J Neurol Sci. 2015;353(1-2):81-3.
Wang - Qi, 2015	Anti-N-methyl-D-aspartate receptor encephalitis concomitant with multifocal subcortical white matter lesions on magnetic resonance imaging: a case report and review of the literature.<br>Wang RJ, Chen BD, Qi D.<br>BMC Neurol. 2015 Jul 8;15:107.
Wang - Wang, 2016	Heterotopic ossification following anti-NMDA receptor encephalitis: a case report.<br>Wang D, Wang S, Huang X, Wang Q.<br>BMC Neurol. 2016 Nov 21;16(1):232.
Wang , Li - Liu, 2017	Anti-N-methyl-D-aspartate receptor encephalitis that aggravates after acinetobacter baumannii pneumonia: A case report.<br>Wang CC, Li DJ, Xia YQ, Liu K.<br>World J Clin Cases. 2017 Sep 16;5(9):368-372.
Warren - Blum, 2017	Atypical N-methyl-D-aspartate receptor encephalitis and a hippocampal tumour.<br>Warren N, Theodoros T, Blum S.<br>Aust N Z J Psychiatry. 2017 Apr;51(4):414-415.
Watanabe - Matsumoto, 2014	Antibodies to neural and non-neural autoantigens in Japanese patients with CNS demyelinating disorders.<br>Watanabe M, Kondo T, Murakata K, Kageyama T, Shibata Y, Takahashi T, Nomura K, Matsumoto S.<br>J Neuroimmunol. 2014 Sep 15;274(1-2):155-60.
Weaver - Griffey, 2016	Anti-N-Methyl-d-Aspartate Receptor Encephalitis as an Unusual Cause of Altered Mental Status in the Emergency Department.<br>Weaver M, Griffey RT.<br>J Emerg Med. 2016 Aug;51(2):136-9.
Wegner - Nabavi, 2014	Anti-leucine rich glioma inactivated 1 protein and anti-N-methyl-D-aspartate receptor encephalitis show distinct patterns of brain glucose metabolism in 18F-fluoro-2-deoxy-d-glucose positron emission tomography.<br>Wegner F, Wilke F, Raab P, Tayeb SB, Boeck AL, Haense C, Trebst C, Voss E, Schrader C, Logemann F, Ahrens J, Leffler A, Rodriguez-Raecke R, Dengler R, Geworski L, Bengel FM, Berding G, Stangel M, Nabavi E.<br>BMC Neurol. 2014 Jun 20;14:136.
Wilson – Fuchs, 2013	Anti-NMDA receptor encephalitis in a 14-year-old female presenting as malignant catatonia: medical and psychiatric approach to treatment.<br>Wilson JE, Shuster J, Fuchs C.<br>Psychosomatics. 2013 Nov-Dec;54(6):585-9.
Wójtowicz – orlicz, 2018	Autoimmune anti-N-methyl-D-aspartate receptor encephalitis - the current state of knowledge based on a clinical case.<br>Wójtowicz R, Krawiec M, Orlicz P.<br>Anaesthesiol Intensive Ther. 2018;50(1):34-39.
Wong - Fries, 2014	Anti-NMDAR encephalitis, a mimicker of acute infectious encephalitis and a review of the literature.<br>Wong D, Fries B.<br>IDCases. 2014 Sep 6;1(4):66-7.
Wong-Kisiel – Mack, 2010	Response to immunotherapy in a 20-month-old boy with anti-NMDA receptor encephalitis.<br>Wong-Kisiel LC, Ji T, Renaud DL, Kotagal S, Patterson MC, Dalmau J, Mack KJ.<br>Neurology. 2010 May 11;74(19):1550-1.
Wright - Vincent, 2016	Neuronal antibodies in pediatric epilepsy: Clinical features and long-term outcomes of a historical cohort not treated with immunotherapy.<br>Wright S, Geerts AT, Jol-van der Zijde CM, Jacobson L, Lang B, Waters P, van Tol MJ, Stroink H, Neuteboom RF, Brouwer OF, Vincent A.<br>Epilepsia. 2016 May;57(5):823-31.
Wright, Hacohen - Vincent, 2015	N-methyl-D-aspartate receptor antibody-mediated neurological disease: results of a UK-based surveillance study in children.<br>Wright S, Hacohen Y, Jacobson L, Agrawal S, Gupta R, Philip S, Smith M, Lim M, Wassmer E, Vincent A.<br>Arch Dis Child. 2015 Jun;100(6):521-6.
Wu - Zhang, 2016	Anti-N-methyl-D-aspartate receptor encephalitis with lung adenocarcinoma.<br>Wu YY, He XJ, Zhang ML, Shi YY, Zhang JW.<br>Neurol Sci. 2016 Sep;37(9):1573-5.
Wu, Feng - Zhang, 2016	Anti-N-Methyl-D-Aspartate Receptor Encephalitis in a Patient with Systemic Lupus Erythematosus.<br>Wu YY, Feng Y, Huang Y, Zhang JW.<br>J Clin Neurol. 2016 Oct;12(4):502-504.
Xiao, Gui - Zhou, 2017	Anti-NMDA-receptor encephalitis during pregnancy: A case report and literature review.<br>Xiao X, Gui S, Bai P, Bai Y, Shan D, Hu Y, Bui-Nguyen TM, Zhou R.<br>J Obstet Gynaecol Res. 2017 Apr;43(4):768-774.
Xiao, Lin - Lü, 2017	Anti-NMDAR encephalitis combined with a subependymoma.<br>Xiao D, Lin Y, Wang X, Yang C, Huang X, Fu B, Wei Q, Lü T.<br>Neurol India. 2017 Mar-Apr;65(2):398-400.
Xia – Dubeau, 2011	Teaching Video NeuroImages: dystonic posturing in anti-NMDA receptor encephalitis.<br>Xia C, Dubeau F.<br>Neurology. 2011 Apr 19;76(16):e80.

© 2021 Nosadini M et al. *JAMA Neurology*.

Xu – Wang, 2011	Anti-N-methyl-D-aspartate receptor encephalitis with serum anti-thyroid antibodies and IgM antibodies against Epstein-Barr virus viral capsid antigen: a case report and one year follow-up.<br>Xu CL, Liu L, Zhao WQ, Li JM, Wang RJ, Wang SH, Wang DX, Liu MY, Qiao SS, Wang JW.<br>BMC Neurol. 2011 Nov 29;11:149.
Xu - Dai, 2016	A Woman with Psychogenic Non-epileptic Seizures and Pelvic Mass.<br>Xu TD, Xu SY, Dai JY.<br>Chin Med Sci J. 2016 Sep 20;31(3):203-206.
Yamanaka - Yokoyama, 2016	Successful management of dexmedetomidine for postoperative intensive care sedation in a patient with anti-NMDA receptor encephalitis: a case report and animal experiment.<br>Yamanaka D, Kawano T, Tateiwa H, Iwata H, Locatelli FM, Yokoyama M.<br>Springerplus. 2016 Aug 22;5(1):1380.
Yang - Guan, 2015	Anti-NMDAR encephalitis after resection of melanocytic nevi: report of two cases.<br>Yang XZ, Cui LY, Ren HT, Qu T, Guan HZ.<br>BMC Neurol. 2015 Sep 14;15:165.
Yang , Zhu - Guan, 2018	Utility and Safety of Intrathecal Methotrexate Treatment in Severe Anti-N-methyl-D-aspartate Receptor Encephalitis: A Pilot Study.<br>Yang XZ, Zhu HD, Ren HT, Zhu YC, Peng B, Cui LY, Guan HZ.<br>Chin Med J (Engl). 2018 Jan 20;131(2):156-160.
Yau – Fung, 2013	Early consideration of anti-NMDAR encephalitis in unexplained encephalopathy.<br>Yau ML, Fung EL.<br>Hong Kong Med J. 2013 Aug;19(4):362-4
Yen – Xiong, 2011	The horse with stripes: a case of anti-NMDA receptor encephalitis.<br>Yen L, Leung M, Kellaher DC, Kukoyi O, Xiong G.<br>Prim Care Companion CNS Disord. 2011;13(4).
Yeshokumar - Pardo, 2016	Gait Disturbance as the Presenting Symptom in Young Children With Anti-NMDA Receptor Encephalitis.<br>Yeshokumar AK, Sun LR, Klein JL, Baranano KW, Pardo CA.<br>Pediatrics. 2016 Sep;138(3). pii: e20160901.
Yilmaz - Tuzun, 2014	Anti NMDA receptor encephalitis associated with thymic hyperplasia: a case report.<br>Yilmaz B, Mastanzade T, Ozkara C, Erkol G, Tuzun E.<br>Neurol India. 2014 May-Jun;62(3):331-2.
Yin, Zhu - Guan, 2018	Resection of melanocytic nevi as a potential treatment of anti-NMDAR encephalitis patients without tumor: report of three cases.<br>Yin H, Zhu C, Ren H, Yang X, Peng B, Cui L, Qu T, Guan H.<br>Neurol Sci. 2018 Jan;39(1):165-167.
Yoga - Ahmed, 2014	A case of non-paraneoplastic anti-N-methyl d-aspartate receptor encephalitis presenting as a neuropsychiatric disorder.<br>Yoga B, Kunc M, Ahmed F.<br>SAGE Open Med Case Rep.
Yokoyama - Yoshida, 2016	Anti-myelin oligodendrocyte glycoprotein antibody neuritis optica following anti-NMDA receptor encephalitis.<br>Yokoyama K, Hori M, Yoshida A.<br>Pediatr Int. 2016 Sep;58(9):953-4.
Yoshimura - Kishi, 201	Anti-N-Methyl-D-Aspartate Receptor Encephalitis Presenting With Intermittent Catatonia.<br>Yoshimura B, Yada Y, Horigome T, Kishi Y.<br>Psychosomatics. 2015 May-Jun;56(3):313-5.
Yu – Moore, 2011	Paraneoplastic encephalitis presenting as postpartum psychosis.<br>Yu AY, Moore FG.<br>Psychosomatics. 2011 Nov-Dec;52(6):568-70.
Yuan - Glezer, 2013	A young woman presenting with psychotic and mood symptoms from anti-N-methyl-D-aspartate receptor (NMDA-R) encephalitis: an emerging diagnosis.<br>Yuan N, Glezer A.<br>Int J Psychiatry Med. 2013;46(4):407-15.
Zandi – Vincent, 2009	Limbic encephalitis associated with antibodies to the NMDA receptor in Hodgkin lymphoma.<br>Zandi MS, Irani SR, Follows G, Moody AM, Molyneux P, Vincent A.<br>Neurology. 2009 Dec 8;73(23):2039-40.
Zhang - Jiao, 2017	Repeated misdiagnosis of a relapsed atypical anti-NMDA receptor encephalitis without an associated ovarian teratoma.<br>Zhang W, Yan L, Jiao J.<br>Neurosci Lett. 2017 Jan 18;638:135-138
Zhang - Jiao, 2018	Early identification of anti-NMDA receptor encephalitis presenting cerebral lesions in unconventional locations on magnetic resonance imaging.<br>Zhang W, Cui L, Wang W, Jiao Y, Zhang Y, Jiao J.<br>J Neuroimmunol. 2018 Jul 15;320:101-106.
Zhang - Zhou, 2018	Late-onset anti-N-methyl-d-aspartate receptor encephalitis in China.<br>Zhang L, Liu X, Jiang XY, Wang YH, Li JM, Zhou D.<br>Epilepsy Behav. 2018 Jul;84:22-28.
Zhang, Li - Wang, 2017	Anesthetic Management of Patients with Anti-N-methyl-D-aspartate Receptor Encephalitis: A Report of Two Cases.<br>Zhang X, Li J, Wang D.<br>Chin Med Sci J. 2017 Apr 10;32(1):62-4.

© 2021 Nosadini M et al. *JAMA Neurology*.

Zhang, Wang, Wang - Guo, 2018	The short-term efficacy of combined treatments targeting B cell and plasma cell in severe and refractory Anti-N-methyl-D-aspartate receptor encephalitis: Two case reports.<br>Zhang XT, Wang CJ, Wang BJ, Guo SG.<br>CNS Neurosci Ther. 2018 Oct 21. doi: 10.1111/cns.13078. [Epub ahead of print] No abstract available.
Zheng, Ye - Lin, 2018	Management of Refractory Orofacial Dyskinesia Caused by Anti-N-methyl-d-aspartate Receptor Encephalitis Using Botulinum Toxin.<br>Zheng F, Ye X, Shi X, Poonit ND, Lin Z.<br>Front Neurol. 2018 Feb 22;9:81.
Zhou – Wang, 2018	An unusual case of anti-MOG CNS demyelination with concomitant mild anti-NMDAR encephalitis.<br>Zhou J, Tan W, Tan SE, Hu J, Chen Z, Wang K.<br>J Neuroimmunol. 2018 Jul 15;320:107-110.
Zhou - Yang, 2015	Anti-N-methyl-D-aspartate receptor encephalitis with occult ovarian teratoma: a case report.<br>Zhou SX, Yang YM.<br>Int J Clin Exp Pathol. 2015 Nov 1;8(11):15474-8.
Ziaeian - Shamsa, 2015	Dazed, confused, and asystolic: possible signs of anti-N-methyl-D-aspartate receptor encephalitis.<br>Ziaeian B, Shamsa K.<br>Tex Heart Inst J. 2015 Apr 1;42(2):175-7.
Zoccarato - Zuliani, 2013	Aquaporin-4 antibody neuromyelitis optica following anti-NMDA receptor encephalitis.<br>Zoccarato M, Saddi MV, Serra G, Pelizza MF, Rosellini I, Peddone L, Ticca A, Giometto B, Zuliani L.<br>J Neurol. 2013 Dec;260(12):3185-7.
Zubair - Majid, 2018	Anti-NMDA Receptor Encephalitis in a Young Girl with Altered Behaviour and Abnormal Movements.<br>Zubair UB, Majid H.<br>J Coll Physicians Surg Pak. 2018 Aug;28(8):643-644.
Zubkov - Kothare, 2015	Teaching NeuroImages: NMDA encephalomyelitis with MRI abnormalities isolated to ventral spinal cord gray matter.<br>Zubkov S, Aggarwal Joshi P, Shepherd TM, Kothare SV.<br>Neurology. 2015 Aug 11;85(6):e55-6.

© 2021 Nosadini M et al. *JAMA Neurology*.
